# Substituent‐Controlled Ring Opening of 1‐Substituted Benzocyclobutenes: Electronic Structure and Diradical Character of *Ortho*‐Quinodimethane Intermediates

**DOI:** 10.1002/chem.202503431

**Published:** 2026-04-21

**Authors:** Magali Dallegre, Imen Abid, Roselyne Rosas, Maxime Dousset, Gaëlle Chouraqui, Laurent Commeiras, Didier Siri, Didier Gigmes, Anthony Kermagoret

**Affiliations:** ^1^ Aix Marseille Univ, CNRS, ICR AMUtech Marseille France; ^2^ Aix Marseille Univ, CNRS, Centrale Med FSCM Marseille France; ^3^ Aix Marseille Univ, CNRS, Centrale Med, ISm2 AMUtech Marseille France; ^4^ Aix Marseille Univ, CNRS, CINaM AMUtech Marseille France

**Keywords:** benzocyclobutene, diels‐alder, *ortho*‐quinodimethane, radical, ring‐opening, TEMPO

## Abstract

Benzocyclobutenes (BCBs) are valuable precursors for the synthesis of bioactive molecules such as steroids, alkaloids, and terpenoids. However, both the ring‐opening temperature (ROT) of BCBs and the precise electronic nature of the resulting reactive intermediate, *ortho*‐quinodimethane (*o*‐QDM) with varying degrees of diradical character, is crucial for controlling reaction outcomes. In this study, we prepared and investigated the ring‐opening behavior of a series of 1‐substituted BCBs bearing ether, amine, amide, or carbamate groups. ROT values, determined by differential scanning calorimetry (DSC), were found to be significantly influenced by the electronic nature of the substituent. The natural bond orbital (NBO) method was employed to assess the orbital contribution of the substituent lone pair, confirming its strong impact on ROT. Thermal activation of these compounds in the presence of either a dienophile or a spin trap (TEMPO) led to the formation of the corresponding Diels–Alder adducts or dialkoxyamines, respectively, consistent with the involvement of a reactive *o*‐QDM intermediate exhibiting partial diradical character. Furthermore, the diradical character of the ring‐opened species was estimated to be 15%–19% using the Natural Orbital Occupation Number (NOON) computational method. The combination of experimental and computational data provides significant insight into the electronic structure governing the ring‐opening process.

## Graphical abstract

Experimental and computational studies elucidate how the electronic nature of substituents controls the ring‐opening temperature and diradical character of 1‐substituted benzocyclobutenes, enabling the rational design of *o*‐quinodimethane‐based synthetic transformations.

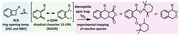

## 1 Introduction

**SCHEME 1:**
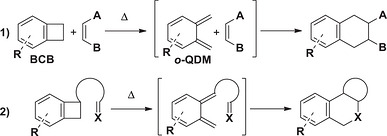
Ring opening of BCB leading to *o*‐QDM species, enabling inter‐ (pathway 1) or intramolecular (pathway 2) DA reactions.

The ring opening of small strained organic rings is a key strategy in the construction of complex molecule frameworks [1, 2, 3, 4, 5, 6, 7, 8]. In this context, benzocyclobutenes (BCBs) stand out for their ability to generate highly reactive intermediates upon ring opening, enabling a wide range of inter‐ and intramolecular cycloaddition [9, 10, 11]. The thermal ring opening of BCB typically proceeds through a conrotatory electrocyclic mechanism [1, 12, 13, 14], generating a transient *ortho*‐quinodimethane (*o*‐QDM) intermediate via *σ*_C─C_ bond cleavage. This transformation forms two new π orbitals at the expense of aromaticity (Scheme 1) [1, 15]. Owing to its reactivity and inherent instability, *o*‐QDM is transient and typically exists only under carefully controlled conditions [16].

The high reactivity of *o*‐QDM has been extensively explored in diels‐alder (DA) reactions with a wide variety of dienophile compounds (Scheme 1) [10, 17, 18, 19, 20, 21]. First developed by Oppolzer et al. [22], intramolecular DA reactions of *o*‐QDM species have been used in the synthesis of alkaloids, steroids, terpenoids, and anthracycline derivatives (Scheme 1) [3, 23, 24]. Beyond Diels Alder reactions, the ring opening of BCB derivatives plays a role in numerous organic transformations [2, 10, 25, 26], including ring expansion reactions [7, 27], electrophilic aromatic substitution reactions [28], heterocyclic compounds synthesis [20, 29, 30], and serves as a platform for building high performance polymeric materials used in electronics and aerospace applications, and other advanced technologies [31, 32, 33, 34, 35].

The ring‐opening behavior of BCB is highly dependent on factors such as ring strain, activation energy [33], and substituent effects [14]. For instance, the unsubstituted BCB requires temperature of approximately 200°C for ring opening [1]. However, electron donating groups on the cyclobutene ring significantly reduce the activation energy for C─C bond cleavage [33], enabling fast ring opening to occur at lower temperatures [36].

The stereochemical outcome of the ring‐opening reaction, referred to as torquoselectivity [14], has been investigated in BCB systems using orbital interaction models [37], which predict the preferred rotational pathways based on substituent orientation and electronic effects [38, 39, 40, 41]. Moreover, theoretical models have shown that favorable orbital interactions during the ring‐opening process can significantly lower the bond dissociation energy of the cyclobutene ring [42, 43].

**SCHEME 2:**
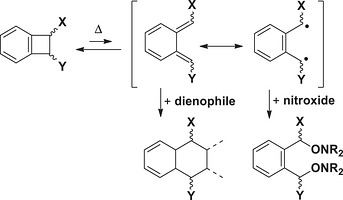
Ring opening of BCB into *o*‐QDM or diradical species, enabling DA or radical coupling pathways.

Interestingly, Korth, Sustmann et al. reported an unusual slow isomerization of a tetrasubstituted BCB at 20°C [44], which contrasted with the expected conrotatory ring‐opening mechanism. This observation suggests the involvement of significant diradical character in the intermediate, rather than a purely concerted electrocyclic process, which may enable isomerization prior to ring closure. Such isomerization has been mentioned in the literature and supports the significant role of the diradical character of intermediates in reactions involving the thermal BCB ring opening reactions (Scheme 2) [17, 45, 46, 47, 48, 49, 50, 51]. Supporting this hypothesis, more recently, Coote, Sherburn et al. demonstrated the formation of di‐alkoxyamines via trapping of diradical intermediates with TEMPO, confirming the diradical character of the intermediates during the BCB ring opening (Scheme 2) [52, 53].

In this contribution, a series of 1‐monosubtituted BCB molecules bearing ethers, amines, amides, or carbamates were synthesized. Their ring‐opening temperatures (ROTs), using differential scanning calorimetry (DSC), were investigated and the electronic effects of the substituents using natural bond orbital (NBO) [54] method were examined. The reactivity of the ring‐opened intermediates was explored using dienophiles and spin traps (Scheme 2). Finally, we assessed the diradical character of the open species through natural orbital occupation number (NOON) method calculations.

## 2 Results and Discussion

### 2.1 BCB Synthesis

**FIGURE 1:**
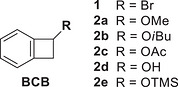
1‐substituted BCB **1** and **2a–e**, prepared according to literature procedures.

**FIGURE 2:**
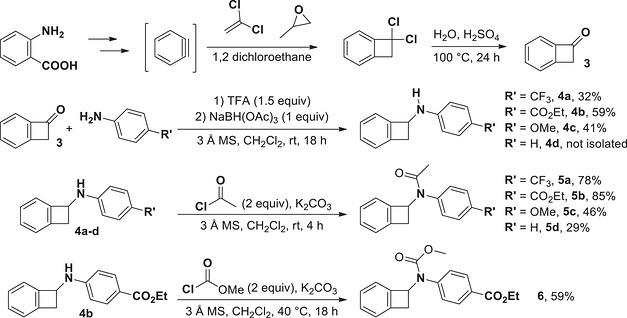
Synthetic Approaches to 1‐Substituted BCB.

To gain deeper insight into these reactivity patterns, we synthesized a representative series of 1‐substituted BCB derivatives. Compounds **1** [55, 56], **2a** [57], **2c** [57], **2d** [57], and **2e** [58] (Figure 1) were synthesized according to procedures reported in the literature (see Supporting Information) by [2+2] cycloaddition of vinylic compounds with in‐situ generated benzyne (Figure 2) [59, 60, 61]. This procedure was adapted to prepare the new BCB **2b**.

Aminobenzocyclobutene derivatives **4a–d** were synthesized from ketone **3** [62] via a reductive amination, following an adapted procedure of Zheng et al. [63] with the corresponding aniline (Figure 2). Compounds **4a–c** were isolated in moderate to good yields (32%–59%, Figure 2). Compound **4d,** however exhibited notable air‐sensitivity and decomposed at low temperature (5°C), even in the absence of light, preventing its isolation. Next, the amine groups of compounds **4a–d** were converted into amide using acetyl chloride under anhydrous conditions, yielding BCBs **5a–d**, respectively, (Figure 2). For compound **5d**, the reaction was carried out directly on crude **4d**. Similarly, the reaction of **4b** with methyl chloroformate afforded BCB **6**. Isolated in moderate to good yields (Figure 2), compounds **5a–d** and **6** proved to be air‐stable at room temperature.

The newly synthesized 1‐substituted BCBs **2b**, **4b**, **4c**, **5a–d**, and **6** were characterized by ^1^H NMR and ^13^C NMR and high‐resolution mass spectrometry (HRMS).

### 2.2 Ring Opening Temperature

With the synthesized BCB derivatives in hand, we next focused on quantifying their ring‐opening temperatures (ROTs) using DSC to better understand the impact of substituent effects on thermal stability and reactivity. Oppolzer defined the ROT as the temperature required to achieve complete conversion of BCB derivatives into their corresponding DA adducts within 18 h [64, 65]. However, in the absence of dienophile molecules, the ring‐opened BCB species tend to undergo side reactions, leading to the formation of various dimers, oligomers and polymers [34]. While the ring opening of BCB is expected to be endothermic, the subsequent reactions of the open species are exothermic. This makes DSC a suitable method for detecting the onset of reactivity through thermal transitions in the thermograms [32, 34, 66].

We assumed that the onset of the exothermic peak in the DSC thermogram could serve as a reliable proxy for the ROT, a hypothesis supported by our observation that unsubstituted BCB (1,2‐dihydrobenzocyclobutene) exhibits a ROT of 200°C (Figure S1) [1]. As shown in Table 1, our DSC results (Figures S2–S14) for BCBs **1**, **2a**, **2d**, and **2e** (entries 1–3, Table 1), aligned well with existing data [48, 57, 67].

**TABLE 1:** ROTs of BCB determined by DSC, and internal orbital interaction energies (*E*_int_) between the ring C─C bond and the lone pair of the heteroatom in substituent **R**.

Entries	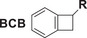	ROT (°C)^a^	σ* Orbital population	*E*_int_ (kcal/mol)
1	**1**, R = Br	180^b^	0.01611	4.35
2	**2a**, R = OMe	110^b^	0.03019	11.99
3	**2b**, R = O*i*Bu	110	0.03114	12.35
4	**2c**, R = OAc	150	0.01995	9.54
5	**2d**, R = OH	85^b^	0.03310	12.87
6	**2e**, R = OSiMe_3_	110^b^	0.02859	12.14
7	**4a**, R = NHPhCF_3_	85	0.03152	12.70
8	**4b**, R = NHPhCO_2_Et	115	0.03136	12.75
9	**4c**, R = NHPhOMe	70	0.03495	13.13
10	**5a**, R = N(COMe)PhCF_3_	135	0.02803	12.74
11	**5b**, R = N(COMe)PhCO_2_Et	140	0.02705	12.17
12	**5c**, R = N(COMe)PhOMe	155	0.02247	8.98
13	**5d**, R = N(COMe)Ph	160	0.01924	8.46
14	**6**, R = N(CO_2_Me)PhCO_2_Et	130	0.02292	11.19

^a^ Temperature error of ± 5°C.

^b^ ROT reported in the literature are 180°C for **1** [67]; 110°C for **2a** [48]; 80°C for **2d** [57]; 100°C for **2e** [67].

As expected [64, 68] the presence of substituent significantly impacted the ROT. Electron‐donating groups such as ethers **2a**, **2b**, and **2e** (entries 2–4, Table 1), hydroxyl **2d** (entry 5, Table 1), or amine **4a–c** (entries 7‐9, Table 1) lowered the ROT to a moderate range (70°C to 115°C). In contrast, less donating groups like acetate **2c** (entry 6, Table 1), amides **5a–d** (entries 10–13, Table 1), and carbamate **6** (entry 14, Table 1) resulted in higher ROTs (130°C to 160°C). These trends confirm the pivotal role of substituent electronic effects in governing the thermal stability of the BCB ring. Building on the DSC results, we sought to rationalize the observed ROT trends by examining the electronic interactions between substituent lone pairs and the key orbitals of the strained C─C bond in the BCB ring [69].

### 2.3 NBO Computations

Previous computational studies have highlighted that substituents can significantly affect the population of the bonding and antibonding orbitals in strained rings [68], thereby altering ring‐opening energetics and torquoselectivity [38, 70, 71, 72, 73].

In this work, we used NBO method to evaluate the electronic population of the *σ* and σ* orbitals of the cyclobutene C─C bond, as well as the internal orbital interaction energy *E*_int_ between the lone pair of the heteroatom and the antibonding σ* orbital [38, 42, 74].

These data provide insight into the substituent‐induced destabilization or stabilization of the BCB ring. High σ* orbital populations and large interaction energies correspond to greater lone pair donation and are associated with lower ROTs.

BCB **2a**,**b** and **2d,e,** which bear electro‐donating ether groups, exhibited relatively high *σ*_c_ *_─_*_c_* electronic populations (ranging from 0.02859 to 0.03110, Table 1) and substantial interaction energies (11.99–12.87 kcal/mol, Table 1), in good agreement with their low ROT values (85°C–110°C, Table 1). In contrast, compound **2c** displayed a lower *σ*_c_ *_─_*_c_* electronic population, consistent with its higher ROT of 150°C (Table 1). Similarly, the amino‐substituted derivatives **4a–c**, showed strong orbital interactions (0.03136–0.03495; 12.70–13.13 kcal/mol; entry 7–9, Table 1), consistent with their low ROT values (70°C–115°C, Table 1). Remarkably, *para*‐substituent on the aniline ring influenced the degree of interaction. The methoxy‐substituted **4c** exhibited the highest internal energy and lowest ROT, while electron‐withdrawing groups in **4a** and **4b** reduced lone pair donation and raised the ROT.

Replacing the amine with an amide group, as in BCB**s 5a–d** led to reduced *σ*_c_ *_─_*_c_* electronic populations (0.02803 and 0.02705, respectively, Table 1) and lower internal energies, with ROTs correspondingly increased to 135°C–160°C (Table 1). BCB **6**, featuring a carbamate group, showed similarly low *σ*_c_ *_─_*_c_* orbital population (0.02292, Table 1), and a moderate internal energy (11.19 kcal/mol), resulting in an intermediate ROT of 130°C (Table 1).

Altogether the NBO results highlight a clear relationship between the electronic nature of the substituent, orbital interactions, and thermal ring opening behavior. The data reinforce the predictive value of computational descriptors in guiding the design of BCB‐based reactive intermediate.

### 2.4 Opening Modes

Encouraged by the strong correlation between electronic structure and ROT, we next sought to explore the nature of the reactive intermediates generated during the thermal ring opening. Specifically, we aimed to evaluate the extent to which diradical character contributes to the ring‐opening process of *o*‐QDM by combining experimental trapping techniques with theoretical analysis.

The ring‐opening of BCB has long been associated with the formation of *o*‐QDM intermediates, inspiring numerous synthetic routes based on the concerted DA reactions [75]. However, emerging experimental evidence has prompted alternative interpretations, suggesting that the ring‐opening process may not be fully concerted and may instead involve a significant degree of diradical character [44, 52].

To probe the electronic nature of the ring‐opened intermediate, we investigated the reactivity of selected BCB derivatives in the presence of either a dienophile (maleic anhydride) or a radical scavenger (TEMPO), in order to evaluate the extent of its diradical character (Scheme 2).

**FIGURE 3:**
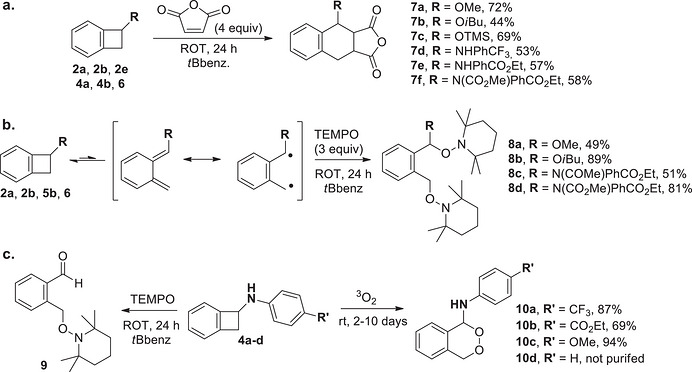
Reactivity of BCB toward (a) dienophile, (b) spin trap, or (c) dioxygen.

DA reactions were conducted at the specific ring‐opening temperature of each compound (Table 1), in *tert*‐butylbenzene (*t*Bbenz), for 24 h in the presence of 4 equiv of maleic anhydride (Figure 3). The DA adducts **7a–f** were successfully obtained from BCBs bearing ether (**2a**,**b**, and **2e**), amino (**4a**,**b)**, or carbamate **6** substituents, respectively, (Figure 3). Although stereochemical assignment is complex, coupling constants observed in ^1^H NMR spectra of **7a–c** (∼3.5 Hz for H_7_,H_8_) are consistent with *cis*‐configurations [76, 77]. NOESY spectra further confirmed the relative configurations, particularly for **7d** and **7e** (Figures S78 and S83, respectively). In contrast, the reaction of maleic anhydride with amide‐substituted BCB **5a–c** failed to yield any DA adducts, even under harsh conditions (150°C, 72 h, 15 equiv of maleic anhydride). Instead, unidentified oligomeric products were observed, likely resulting from *o*‐QDM self‐additions [1, 57, 78]. Attempts to use more electron‐rich dienophiles such as *iso*butyl vinyl ether, allylic alcohol, or diphenylacetylene, were also unsuccessful.

We next evaluated the diradical reactivity of the ring‐opened intermediates via spin trapping experiments using TEMPO, a well‐established strategy for the capture of transient radical species [50, 52, 53].

A kinetic study of the ring opening of BCB **2a** at 110°C in *t*Bbenz with three equiv of TEMPO (Figure 2) showed a gradual disappearance of the characteristic cyclobutene ^1^H NMR signals, accompanied by new peaks, consistent with product formation (Figure S89). After 24 h, full conversion of **2a** was achieved. HRMS analysis confirmed the presence of the di‐TEMPO‐alkoxyamine adduct **8a**. The same strategy applied to **2b**,**c**, **4a–c**, **5b**, and **6** yielded similar results (Table 1). In the case of **2b**, the di‐alkoxyamine adduct **8b** was isolated. However, compound **2c** degraded under reaction conditions, as previously reported in the literature [57], thus preventing product isolation.

Amino‐substituted derivatives **4a–c** gave rise to an unexpected benzaldehyde‐TEMPO adduct **9** rather than the expected di‐TEMPO adduct (Figure 3). This is likely due to the hydrolytic instability of the initially formed di‐alkoxyamines, leading to N─O bond cleavage (Scheme S1). The oxidation of the resulting amine into an imine would be catalyzed by TEMPO [79, 80] and the presence of moisture hydrolyzed the product to **9**, as the only isolated product of the reaction.

In contrast, the amide‐containing **5b** gave the expected di‐TEMPO‐adduct **8c** in good yield. Compound **6** also afforded the corresponding di‐alkoxyamine **8d** in 24 h at 105°C, with minimal byproduct formation, in 81% yield.

**FIGURE 4:**
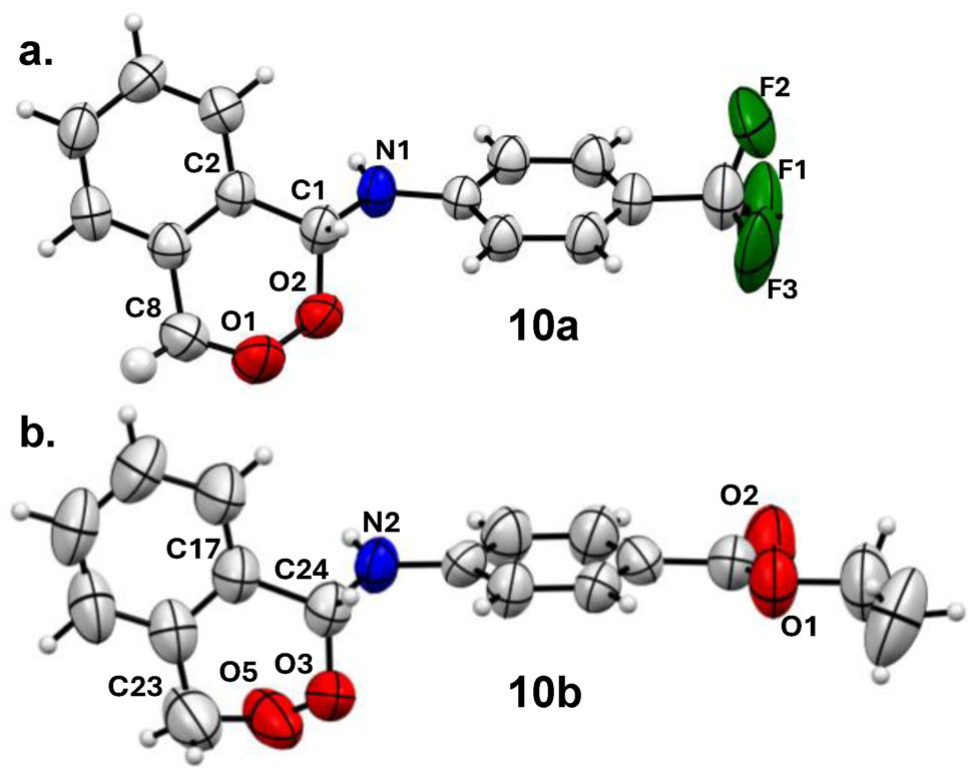
Molecular structure of (a) **10a** (R enantiomer of one conformer of the solid solution, see Supporting Information) and (b) **10b** (R enantiomer of one conformer of the enantiomeric solid solution, see ESI) showing 50% probability ellipsoids. O─O bond lengths (Å) for **10a**: O1‐O2 = 1.438(3), O3‐O4 = 1.460(5); for **10b**: O3‐O5 = 1.479(10), O6‐O7 = 1.431(9).

Interestingly, we observed O_2_ sensitivity of **4c** and **4d**, with rapid formation (less than 24 h) of peroxide species **10c** and **10d** upon standing in air. On the other hand, **4a** and **4b** required longer exposure (2 and 10 days, respectively, under oxygen atmosphere, Figures S110 and S115) to undergo similar oxidation to peroxides **10a** and **10b**, respectively, (Figure 3). as confirmed by their respective x‐ray crystal structures [81, 82] (Figure 4, detailed in Supporting Information: interestingly, the crystallization of peroxide **10a** afforded a solid solution, while peroxide **10b** yielded an enantiomeric solid solution [83, 84]). These results suggest that the rate of oxidation correlates with the ease of ring opening, consistent with the ROT trends.

The exact mechanism of peroxide formation remains a subject of debate. Two plausible pathways: (1) a direct reaction of triplet molecular oxygen with the *o*‐QDM intermediate, or (2) spin trapping of carbon‐centered diradicals by molecular oxygen. Given the well‐documented efficiency of triplet oxygen toward radical species, the latter pathway is particularly compelling. Notably, the formation of such peroxide species from BCBs has been previously reported by Roth and coworkers [45, 85], by Korth, Sustmann et al. [44] and by Carissan, Commeiras, Parrain, and collaborators [43] supporting the idea that ring‐opening intermediates, particularly diradicals, play a key role in aerobic oxidation. These findings also contribute to a broader mechanistic discussion. For instance, to rationalize the racemization of BCB derivatives an outcome that appears incompatible with a conrotatory ring‐opening/closing mechanism governed by Houk's torquoselectivity rules [14], Korth and Sustmann proposed that ring opening could proceed via radical intermediates [44]. These species would bypass the torquoselectivity constraints [86].

This issue fuelled an ongoing debate as to whether triplet molecular oxygen reacts preferentially with *o*‐QDM double bonds, or with the transient carbon‐centered diradicals generated upon ring opening [87, 88]. Given the established radical reactivity of triplet O_2_ [89, 90], the latter pathway appears most plausible and consistent with peroxide products. The very recent results of Wahl et al. on photoactivation of acetophenone derivatives support a mechanism involving the reaction of ^3^O_2_ with diradicals leading to peroxides [91]. As a general note, we emphasize the high reactivity of amino‐substituted BCBs and their sensitivity toward O_2_, which should be taken into account during storage and handling.

Since the thermal activation of **2a** led to both the DA adduct **7a** and the di‐alkoxyamine **8a** in good yields (Figure 3), we designed a competition experiment to probe the relative reactivity of the *o*‐QDM and diradical pathways. BCB **2a** was heated at 110°C for 24 h in *tert‐*butylbenzene in the presence of maleic anhydride (4 equiv) and TEMPO (3 equiv). Surprisingly, only the characteristic signals of the DA adduct **7a** were observed in the ^1^H NMR spectrum of the crude reaction mixture. No signal corresponding to the di‐alkoxyamine **8a** was detected. These results suggest that under these thermal conditions, the reaction proceeds predominantly through the non‐radical pathway rather than via diradical intermediates. The preference for the non‐radical *o*‐QDM mechanism is consistent with previous findings. For instance, Coote, Sherburn, and coworkers reported the formation of spirodimers upon activation of 1,2‐dihydrobenzocyclobutene, resulting from [4+2] cycloaddition rather than radical coupling process [52].

### 2.5 NOON and Diradical Character

To gain a deeper understanding of the nature of the ring‐opened‐intermediates, we applied NOONs to evaluate their diradical character [92]. In a purely closed‐shell electronic configuration, where all electrons are paired, these orbitals typically exhibit occupation numbers close to 2 and 0, respectively. However, in diradical systems these values shift toward 1, reflecting the presence of two unpaired electrons. Thus, the extent of deviation from the closed‐shell ideal provides a direct estimate of the diradical character. This theoretical approach has been successfully employed by Gaudel‐Siri et al. to confirm the diradical nature of intermediates involved in a cascade enyne‐allene rearrangement mechanism [93]. Michl et al. observed partial singlet diradical character in species generated from BCB activation using electronic spectroscopy [94]. Later, Koutecky et al. demonstrated the partial radical nature of the ring‐opened BCB species using the NOON method, reporting values of 1.7651/0.2349. These results indicated that while the diamagnetic character associated with *o*‐QDM formation was predominant a significant diradical contribution must also be considered [92].

**FIGURE 5:**
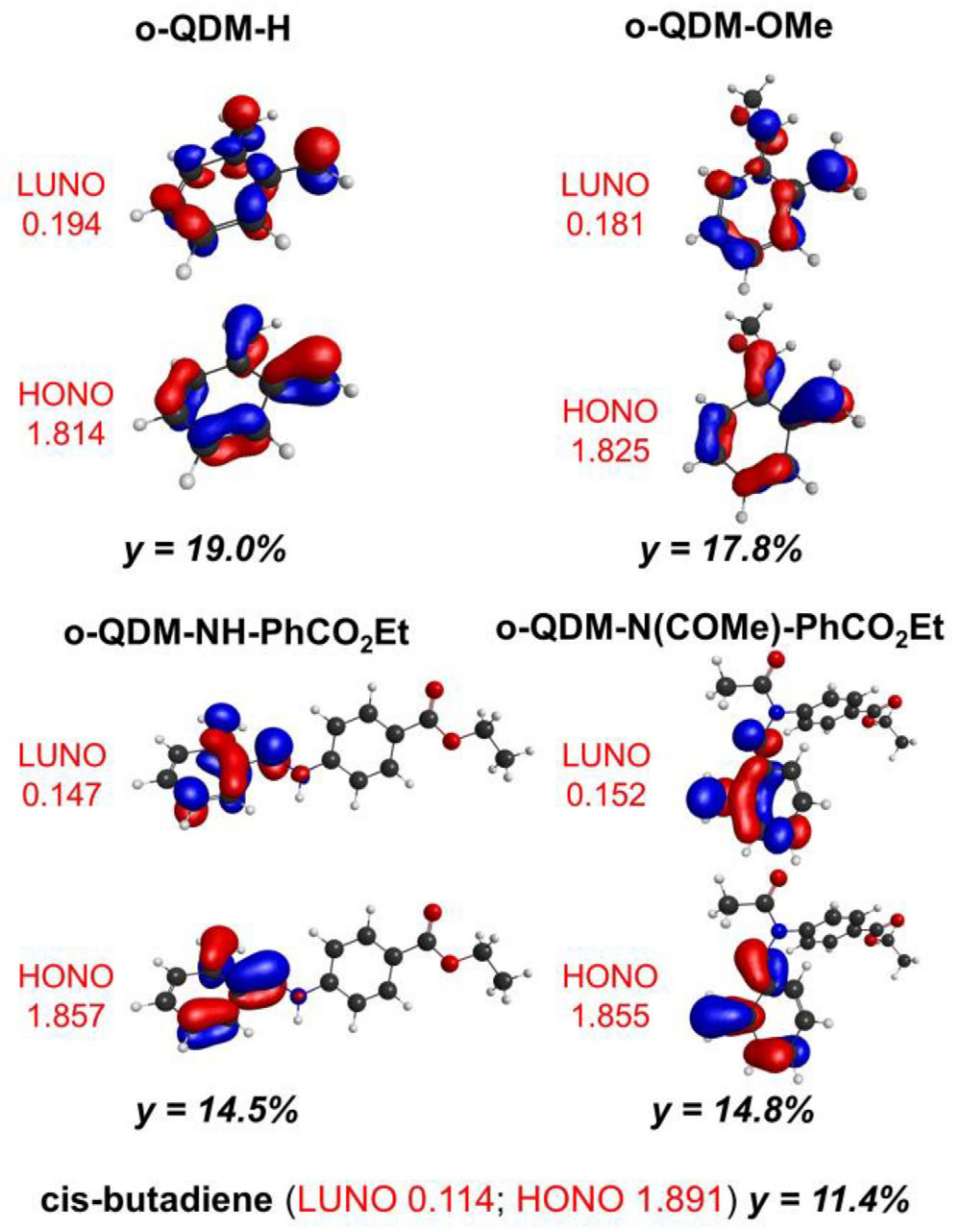
Representation of the natural orbitals for *o*‐QDM‐H, *o*‐QDM‐OMe, *o*‐QDM‐NHPhCO_2_Et, and *o*‐QDM‐N(COMe)PhCO_2_Et and their respective occupation numbers in red (LUNO and HONO) to calculate the *y* number. The *y* number of *cis*‐butadiene is shown below.

In the present work, NOON calculations were performed at the CAS(8,8)/6‐31G(d) level using GAMESS [95] focusing on *o*‐QDM‐H, *o*‐QDM‐OMe, *o*‐QDM‐NHPhCO_2_Et, and *o*‐QDM‐N(COMe)PhCO_2_Et (Scheme S2). The two molecules were fully optimized at the CAS(8,8)/6‐31G(d) level followed by frequency calculations to ensure that the obtained geometries correspond to true minima. The eight frontier orbitals used in the calculation, along with their respective occupation numbers are shown in Figure 5. The diradical character ***y*** was estimated directly from the occupation number of the LUNO (lowest unoccupied natural orbital) obtained from CAS calculations (simple model of 2 electrons in 2 orbitals) [96, 97], resulting in a diradical character of approximatively 19% and 18% for *o*‐QDM‐H and *o*‐QDM‐OMe, respectively, (Table S2). These values are consistent with Valence Bond calculations reported by Pei et al. [53], who reported a value of 30% for *o*‐QDM‐H. The LUNO occupation number for o‐QDM‐NHPhCO_2_Et and *o*‐QDM‐N(COMe)PhCO_2_Et is approximately 15%, indicating that the ring opening of amino‐substituted BCBs exhibits diradical reactivity similar to that of amido‐substituted derivatives.

Overall, the NOON results support the view that the ring‐opening process leads to intermediates exhibiting a hybrid character‐predominantly closed‐shell (*o*‐QDM‐like), but with a detectable diradical contribution. This dual nature likely underpins the divergent reactivity patterns observed experimentally and offers a framework for the rational design of new synthetic strategies leveraging either or both reactive species.

## 3 Conclusions

A series of 1‐substituted BCB, bearing bromide, ether, hydroxy, acetate, amine, amide, or carbamate functional groups was synthesized. Depending on the nature of the substituent, their ROTs, determined by DSC, ranged from 70°C to 180°C, with electron‐donating groups significantly lowering the activation threshold.

NBO calculations revealed a strong correlation between the lone pair (of the BCB substituent) interactions and the *σ*_C─C_* (bond of the cyclobutene ring) orbital population, providing a rationale for the observed thermal reactivity. These calculations confirmed that electron‐donating groups such as ethers or amines favor thermal ring opening at low temperatures, whereas amide and carbamate substituents increase the ROT.

Furthermore, experimental trapping reactions using dienophiles (DA reaction), spin traps (di‐alkoxyamine synthesis), and triplet oxygen (peroxide synthesis) uncovered a dual reactivity mode, confirming the generation of *o*‐QDMs with a significant diradical character upon BCB thermal ring opening.

NOONs method quantitatively confirmed that these intermediates possess a non‐negligible diradical character of 19%–15%, supporting a hybrid mechanism in which closed‐shell and open‐shell reactivity pathways coexist. Substituents were found to influence the diradical character only moderately; increasing this contribution while maintaining moderate ROT values could enable paramagnetic characterization and shift the balance between DA and radical pathways.

Overall, these findings highlight the importance of combining thermal analysis, reactivity assays, and theoretical modeling to control the balance between low‐temperature reactivity and storage stability, especially with respect to O_2_ sensitivity. The present study clarifies the role of substituent on the electronic effects in BCB ring opening and provides a unified picture of the electronic structure and reactivity of the resulting intermediates. This improved mechanistic understanding should facilitate the rational design of new synthetic and polymerization strategies based on BCB activation.

## Conflicts of Interest

The authors declare no conflicts of interest.

## Supporting information




**Supporting file**: The detailed syntheses and characterization of the compounds are described in the Supporting Information with additional references [98, 99, 100, 101]. DSC thermograms (Figures S1–S14), NMR spectra (Figures S15–S126), and x‐ray molecular structures of **10a** and **10b**. DFT calculations are provided in the Supporting Information with additional references [102].

## Data Availability

The data that support the findings of this study are available from the corresponding author upon reasonable request.
