# Supplementary material for: Substituent‐Controlled Ring Opening of 1‐Substituted Benzocyclobutenes: Electronic Structure and Diradical Character of Ortho‐Quinodimethane Intermediates
Source: Chemistry. 2026 Apr 21;32(25):e03431. doi: 10.1002/chem.202503431 (PMC13331574; doi:10.1002/chem.202503431)
Supplement: Supplementary file 1 — Supporting file: The detailed syntheses and characterization of the compounds are described in the Supporting Information with additional references [98, 99, 100, 101]. DSC thermograms (Figures S1–S14), NMR spectra (Figures S15–S126), and x‐ray molecular structures of 10a and 10b. DFT calculations are provided in the Supporting Information with additional references [102]. [file CHEM-32-e03431-s001.docx]

Supporting information

For

Substituent-Controlled Ring Opening of 1-Substituted Benzocyclobutenes: Electronic Structure and Diradical Character of ortho-Quinodimethane Intermediates

Magali Dallegre,^a^ Didier Siri,^a^ Imen Abid,^a^ Roselyne Rosas,^a^ Maxime Dousset,^a^ Gaelle Chouraqui,^b^ Laurent Commeiras,^b^ Didier Gigmes^a^* and Anthony Kermagoret^c^*

| ***1/ Chemical compounds*** | ***2*** |
| --- | --- |
| ***2/* NMR Measurements** | ***2*** |
| ***3/* Mass spectrometry** | ***2*** |
| ***4/ Single crystal X-ray diffraction*** | ***2*** |
| ***5/ Differential scanning calorimetry (DSC)*** | ***3*** |
| ***6/ DSC thermograms of BCBs*** | ***3*** |
| ***7/ BCB synthesis*** | ***7*** |
| ***8/ NMR spectra*** | ***19*** |
| ***9/ X-ray molecular structures of 10a and 10b*** | ***95*** |
| ***10/ Computational part*** | ***97*** |
| ***11/ References*** | ***100*** |

***1/ Chemical compounds***

Chemicals: 1,2-dihydrobenzocyclobutene, cycloheptatriene, CHBr_3_, *N*-bromo-succinimide (NBS), benzoyl peroxide (BPO), sodium hydride (NaH), anthranilic acid, isoamyl nitrite, trifluoroacetic acid (TFA), K_2_CO_3_, sodium triacetoxyborohydride, isobutyl vinyl ether, vinyl acetate, *N*,O-bistrimethylsilyl acetamide (bTMS-EA), aniline, 4-(trifluoromethyl)aniline, 4-(ethylacetate)aniline, 4-(methoxy)aniline, acetyl chloride, methyl chloroformate, (2,2,6,6-tétraméthylpipéridin-1-yl)oxy (TEMPO), maleic anhydride, isobutylvinyl ether, allylic alcohol, diphenylacetylene, methanol, ethanol, diethylether, pentane, ethylacetate, dichloroethane (DCE), CH_3_CN, and acetone, were purchased from commercial suppliers (Aldrich, Acros, TCI or ABCR) and used without further purification. Molecular sieves 3 Å (3 Å MS) were purchased from Aldrich and dry *under vacuum* at 120 °C for 2 h before utilization. Commercial anhydrous-grade solvents, such as *tert*butybenzene (*t*Bbenz), CH_2_Cl_2_, benzene, and toluene were purchased from Aldrich, dried over 3Å MS, and degassed under argon before use. Benzocyclobutanone **3** was synthesized according to the literature (Caution: the zwitterionic salt can spontaneously explode; to ensure safe handling it must be transferred before residual solvent is fully removed).^1^

***2/* NMR Measurements**

NMR spectra were recorded on Bruker AVL 300, 400 and 500 spectrometers (^1^H-NMR 300.13, 400.13 and 500.13 MHz and ^13^C-NMR 100.60, and 125.75 MHz). Chemical shifts (δ) are reported in ppm relative to residual solvent signals. *Signal multiplicities* are reported as follows: s, singlet; d, doublet; dd, doublet of doublets; t, triplet; q, quartet; sep, septet; m, multiplet (br, broad; app, apparent). 2D NMR spectra (COSY and NOESY) were acquired using standard Bruker pulse sequences. *All spectra are provided in the ESI. Deuterated solvents (CDCl_3_, C_6_D_6_) were purchased from Aldrich and dry over 3 Å MS, and degassed under argon when necessary.*

***3/* Mass spectrometry**

High-resolution mass spectra (HRMS) were recorded on a Synapt G2 HDMS mass spectrometer (Waters, Manchester, UK) equipped with a pneumatically assisted electrospray ionization (ESI) source operated at 35 °C in positive ion mode (electrospray voltage: -2.27 or +2.8 kV; declustering potential: +20 V; nebulizing gas flow: N_2_, 100 L.h^–1^). Mass spectra were acquired using an orthogonal acceleration time-of-flight (oa-TOF) mass analyzer. Sample solutions in CH_2_Cl_2_ were diluted (1:10) with methanol prior to injection in the ESI source at a flow rate of 10 μL.min^–1^ flow rate using a syringe pump. Accurate mass measurements were performed in triplicate using an external calibration for the oa-TOF mass analyzer. Instrument control, data acquisition and data processing were performed with the MassLynx 4.1 programs provided by Waters.

**4/ Single crystal X-ray diffraction**

Suitable crystals were selected and mounted on a Rigaku Oxford Diffraction SuperNova Atlas S2 diffractometer. Crystals were kept at 295 K during data collection. Using Olex2,^2^ the structure was solved with the SHELXT^3,4^ structure solution program using Intrinsic Phasing and refined with the SHELXL refinement package using Least Squares minimization. All H-atoms were found experimentally and refined as riding atoms with their Uiso parameters constrained to 1.2 Ueq (parent atoms). CCDC 2492651 and 2494057 contain the supplementary crystallographic data for **10a** and **10b**, respectively. These data are provided free of charge by the joint Cambridge Crystallographic Data Centre and Fachinformationszentrum Karlsruhe <url href="[http://www.ccdc.cam.ac.uk/structures](http://track.editorialmanager.com/CL0/http:%2F%2Fwww.ccdc.cam.ac.uk%2Fstructures/1/010f019ce619e90f-27f3613d-a097-4647-af1a-8ffc014f893d-000000/tMoW-y4-TCtxjEIJ2RHsncgQxcky80pUbRbSbASmWJo=254)">Access Structures service</url>.

***5/ Differential scanning calorimetry (DSC)***

Ring-opening temperatures (ROT) of benzocyclobutene (BCB) derivatives were determined using a DSC Q20 instrument TA Instruments. The measurement sequence consisted of an isothermal hold at 0 °C for 5 min, followed by a heating ramp of 10 °C min^−1^ up to 250 °C. Sample masses ranged from 8 to 15 mg. ROT values were extracted from the DSC thermograms using TA Instruments Universal Analysis V4.5A software, and correspond to the onset of the exothermic signal (see Figures S1-S14). An experimental error of ± 5 °C was considered for the ring-opening temperatures of BCBs.

***6/ DSC thermograms of BCBs***

***7/ BCB synthesis***

**1-bromo-benzocyclobutene BCB-Br 1 (CAS 21120-91-2)**

Method A (reported procedure^5^) : In a 500 mL round bottom flask, cycloheptriene (55 g, 0.60 mol), bromoform (52 g, 0.20 mol), K_2_CO_3_ (30 g, 0.22 mol), 18-crown-6 (1.5 g, 0.006 mol) were stirred at 120 °C for 24 hours. After completion, 100 mL of acetone were added, and the mixture was filtered and washed with acetone. The filtrate was evaporated under reduced pressure. The dark residue was transferred to hot pentane and filtered to remove solid. The volatiles were removed under reduced pressure and the residual oil was distilled under vacuum using a liquid nitrogen trap at 41-45 °C. Most of BCB-Br was recovered in the nitrogen trap.

Method B (reported procedure^6^): Another strategy consisted of the grafting of a bromide group onto benzocyclobutane using the *N*-bromosuccinimide/benzylperoxide system and led to **1** in good yield (73%).

In a 250 mL round bottom flask, *N*-bromosuccinimide (12 g, 67 mmol), benzoyl peroxide (0.12 g, 0.5 mmol), benzocyclobutene BCB-H (5 g, 48 mmol), and 60 mL of chlorobenzene (ClBn) were stirred at 85 °C for 3 days. After completion, 40 mL of pentane were added and the mixture was filtered and washed with pentane. All volatiles were removed under reduced pressure, and the crude oil was distilled under vacuum (2 mbar) at 70-74 °C to afford BCB-Br as an pale yellow oil. (6.3 g, 73%).

^1^H NMR (300MHz, CDCl_3_) : δ (ppm) = 7.00-7.35 (overlapped signals with CHCl_3_, m, 4H, H_2-5_), 5.39 (br s, 1H, H_8_), 3.85 (d, *J* = 15 Hz, 1H, H_7_), 3.43 (d, *J* = 15 Hz, 1H, H_7’_). (Figure S15)

**1-Methoxybenzocyclobutene BCB-OMe 2a (CAS 36101-23-2)^7^**

To 90 mL of a methanol solution containing 9.3 g of sodium methoxide (172 mmol, 5 equiv.) was added 6.30 g of 1-bromobenzocyclobutene **1** at 0 °C. The mixture was stirred at 68 °C for 24 h. After cooling to room temperature, methanol was removed under reduced pressure, and 100 mL of cold water was carefully added at 0 °C. The product was extracted three times with diethylether, and the combined organic layers were dried over MgSO_4_. Filtration and evaporation of the solvent under reduced pressure gave a crude residue, which was purified by vacuum distillation (0.5 mbar) to afford **2a** as a colourless oil (1.27 g, 28%).

^1^H NMR (300MHz, CDCl_3_) : δ (ppm) = 7.20-7.35 (overlapped signals with CHCl_3,_ m, 3H, H_ar_), 7.15 (d, *J* = 6 Hz, 1H, H_ar_), 5.00 (d, *J* = 6 Hz, 1H, H_8_), 3.49 (s, 3H, H_9_), 3.44 (dd, *J* = 8, 12 Hz, 1H, H_7_), 3.12 (dd, *J* = 3, 15 Hz, 1H, H_7’_). (Figure S16)

**1-isobutoxybenzocyclobutene BCB-OiBu 2b and 1-acetatebenzocyclobutene BCB-OAc 2c**^7^

***1-(isobutyl)benzocyclobutene* 2b**

To a solution of anthranilic acid (6.2 g, 45 mmol) in ethanol (60 mL) at 0 °C, were added a catalytic amount of TFA (100 μL) and 2 equiv of isoamyl nitrite (12 mL, 10.4 g, 89 mmol) dropwise. The mixture was stirred for 1 h at 0 °C and 1 h at room temperature (25 °C). The produced zwitterionic salt was filtered, carefully washed with diethylether (3 x 50mL) and rapidly transferred onto the flask containing 2 equiv isobutylvinyl ether (13 mL, 10.0 g, 100 mmol) dissolved in 40 mL of DCE (**Caution : the zwitterionic salt is highly unstable and may detonate spontaneously. The solid must not be dried completely before transfer**). The mixture was stirred at room temperature for 1 h and then heated at 80 °C for 24 h. liberation of CO_2_ and N_2_ indicated the formation of benzyne. After reaction, all volatiles were removed under reduced pressure at room temperature. The benzocyclobutene **2b** was isolated by distillation under reduced pressure (0.9 mbar) at 100-110 °C and purified by silica gel chromatography using pentane-CH_2_Cl_2_ (4:1) to yield a colorless oil (2.1 g, 27%).

^1^H NMR (300 MHz, CDCl_3_) : δ (ppm) = 7.36-7.24(m, 3H, overlapped signals of *H*_ar_), 5.03 (dd, *J* = 1.8, 2.1 Hz, 1H, H_8_), 3.49 (ddd, *J* = 16.5, 4.8, 0.9 Hz, 1H, H_7_), 3.41 (ddd, *J* = 15.5, 9.0, 6.7 Hz, 2H, H_9_), 3,15 (ddd, *J* = 16.5, 2.4, 0.9 Hz, 1H, H_7’_), 1.92 (app sept, *j* = 6.7 Hz, 1H, H_10_), 0,96 (d, *J* = 6,3 Hz, 6H, H_11_).

^13^C NMR (75 MHz, CDCl_3_) : δ (ppm) = 146.6 (s, *C*_ar6_), 142.6 (s, *C*_ar1_), 129.2 (s, *C*H_ar_), 126.9 (s, *C*H_ar_), 123.5 (s, *C*H_ar_), 122.8 (s, *C*H_ar_), 76.8 (s, *C*H_8_), 75.6 (s, *C*H_9_), 38.6 (s, *C*H_7_), 28.6 (s, *C*H_10_), 19.5 (app d, *J* = 1.7 Hz, *C*H_11_).

HRMS (ESI) calcd for [M+H]^+^ [C_12_H_17_O]^+^: *m/z* 177.1276; found 177.1272

NMR spectra of **2b**: Figures S17-19

***1-(acetoxy)benzocyclobutene* 2c (CAS 3469-03-2)**^7^

The BCB **2c**^7^ was prepared according to the literature and obtained as a crude product and used without further purification.

^1^H NMR (400 MHz, CDCl_3_) δ 7.31 (m, 1H, H_ar_), 7.23 (m, 2H, H_ar_), 7.14 (d, *J* = 7.3 Hz, 1H, H_ar_), 5.90 (dd, *J* = 1.9, 4.0 Hz, 1H, H_8_), 3.64 (dd, *J* = 4.8, 14.4 Hz, 1H, H_7_), 3.21 (dd, *J* = 2.0, 14.5 Hz, 1H, H_7’_), 2.10 (s, 3H, H_10_).

^13^C NMR (101 MHz, CDCl_3_) δ 171.2 (s, C_9_), 144.3 (s, C_1_ or C_6_), 142.9 (s, C_1_ or C_6_), 130.1 (s, C_ar_), 127.7 (s, C_ar_), 123.7 (s, C_ar_), 123.35 (s, C_ar_), 71.8 (s, *C*H_8_), 39.05 (s, *C*H_7_), 21.1 (s, *C*H_10_). NMR spectra of **2c** : Figure S20

**1-hydroxybenzocyclobutene BCB-OH 2d (CAS 35447-99-5)^7^**


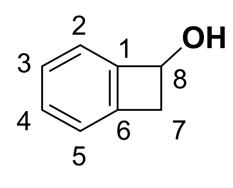
 Compound BCB-OAc **2c** (2.9 g, 18 mmol, 1 equiv.) was dissolved in 40 mL of methanol and 2 mL of water. To this solution was added 2.5 g of K_2_CO_3_ (18 mmol, 1 equiv.) and the mixture was stirred at room temperature (25 °C) for 24 h. After completion, the reaction mixture was extracted with diether ether (2 x 50mL). The combined organic layers were washed with water, dried over MgSO_4_, and concentrated under reduced pressure. The residue was recrystallized from pentane to afford the product as a white solid (63% yield). Melting point 62 °C (DSC, Fig S5)

^1^H NMR (300 MHz, CDCl_3_) : δ (ppm) = 7.14-7.30 (overlapped signals with CHCl_3_, m, 4H, H_2-5_), 5.31 (dd, *J=* 4.5, 4.5 Hz, 1H, H_8_), 3.62 (dd, *J*= 1.8, 12,3 Hz, 1H, H_7_), 3.07 (dd, *J*= 2.1, 12.3 Hz, 1H, H_7_), 1.55 (s, 1H, OH). NMR was in agreement with the literature.^7^ (Figures S21-22)

**1-trimethylsiloxybenzocyclobutene BCB-OTMS 2e (CAS 81522-30-7)^8^**

Compounds BCB-OH **2d** (1.01 g, 8.5 mmol, 1 equiv.) and *N,O*-bis(trimethylsilyl)acetamide (bTMS-EA, 2.0 mL, 8.5 mmol, 1 equiv.) were dissolved in 43 mL of acetonitrile and stirred at room temperature (25 °C) for 1.5 h. After completion, all volatiles were removed under reduced pressure, and the residue was purified by silica gel chromatography using pentane-diethyl ether (98:2) to afford the product as a colorless oil (56% yield).


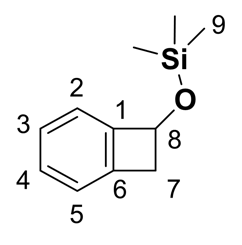


NMR ^1^H (300 MHz, CDCl_3_) : δ (ppm) = 7.1-7.3 (overlapped signals with CHCl_3_,m, 4H, H_2-5_), 5.27 (dd, *J* = 4.5, 4.5 Hz, 1H, H_8_), 3.56 (dd ; *J* = 4.5, 14.1 Hz, 1H, H_7_), 3.1 (dd ; *J* = 2.1, 13.8 Hz; 1H ; H_7_), 0.28 (s, 9H, H_9_).

NMR ^13^C (75 MHz, CDCl_3_) δ 147.9 (s, *C*_ar_), 141.9 (s, *C*_ar_), 128.9 (s, *C*H_ar_), 126.8 (s, *C*H_ar_), 123.3 (s, *C*H_ar_), 122.1 (s, *C*H_ar_), 70.0 (s, *C*_8_), 41.95 (s, *C*_7_), 0.0 (s, *C*_9_).

NMR spectra of **2e**: Figures S23-26

**Benzocyclobutanone 3 (CAS 3469-06-5)^1^**

To a solution of anthranilic acid (21.5 g, 1 equiv.) in ethanol (300 mL) at 0 °C, were added a catalytic amount of TFA (200 μL) and 2 equiv. of isoamyl nitrite dropwise (50 mL). The mixture was stirred for 1 h at 0 °C, then for 2 h at room temperature (25 °C). 300 mL of diethylether were then added, and the resulting zwitterionic salt was collected by filtration, carefully washed with diethylether and immediately transferred to a reaction flask.

Caution: the zwitterionic salt is highly unstable and may detonate spontaneously ~~explode~~. The solid must not be dried completely before transfer.

The wet solid was added to a mixture of 110 mL of 1,1-dichloroethylene and 400 mL of 1,2-dichloroethane. The mixture was stirred at room temperature for 1 h and then heated at reflux for 18 h. Liberation of CO_2_ and N_2_ indicated the formation of benzyne. After completion, the mixture was filtered, and the filtrate was concentrated under reduced pressure to 100mL. A 3% aqueous solution of H_2_SO_4_ (100 mL) was added, and the mixture was heated at reflux for 24 h.

After completion and cooling to room temperature, the mixture was extracted with diethylether (4 × 50 mL). The combined organic extracts were washed with aqueous NaHCO_3_, dried over MgSO_4_, and concentrated under reduced pressure. The crude product was purified by vacuum distillation (55-60 °C, 2 mbar) to afford **3** as a colorless oil.

^1^H NMR (300 MHz, CDCl_3_) : δ (ppm) = 7.54-7.33 (m, 4H, H_ar_), 3.99 (s, *2*H, -C*H_2_*). (Figure S27)

***General procedure for synthesis of amino-BCBs 4a-d.***

Benzocyclobutanone **3** (1 equiv.) and the corresponding aniline (1 to 2 equiv.) were dissolved in 100 mL of anhydrous dichloromethane under argon in the presence of 3Å MS (2 g per g of aniline). At 0 °C, TFA (5 equiv.) was added dropwise, and the solution was stirred at room temperature (25 °C) for 30 minutes. Compound NaBH(OAc)_3_ (2 equiv.) was slowly added portion wise under argon, and the mixture was stirred at reflux for 18 h under argon. After completion, 50 mL of saturated aqueous NaHCO_3_ solution were added, and the organic phase was extracted with dichloromethane. The combined organic extracts were washed with an aqueous saturated solution of NaHCO_3_ (3x 50mL), then with brine (1x 50mL), dried over MgSO_4_, and concentrated under reduced pressure. The crude amino-BCB products were purified with suitable methods (see compound-specific procedures). Caution : amino-BCBs are sensitive to O_2_ in solution, their solutions have to be prepared under inert atmosphere (for ex: NMR solutions).

***1-N-[p-(trifluoromethyl)phenyl]benzocyclobutene 4a (CAS 2097692-28-7)^9^***

Synthesis details: **3** (2.0 g, 17 mmol), 4-(trifluoromethyl)aniline (5.7 g, 35 mmol) and NaBH(OAc)_3_ (7.3 g, 35 mmol) were reacted following the general procedure. BCB **4a** was purified by column chromatography using pentane and EtOAC gradient (0 to 10%). Yield: 32%. White solid, melting point 70 °C (DSC, Figure S7).

^1^H NMR (400 MHz, ) δ 7.45 (d, *J* = 8.6 Hz, 2H, *H*_10_), 7.33 (t, *J* = 7.2 Hz, 1H, *H*_4_), 7.27 (t, *J* = 7.2 Hz, 1H, *H*_3_), 7.25 (m, overlapped signals of *H*_5_ and C*H*Cl_3_), 7.18 (d, *J* = 7.2 Hz, 1H, *H*_2_), 6.71 (d, *J* = 8.6 Hz, 2H, *H*_11_), 5.10 (dd, *J* = 4.3, 2.0 Hz, 1H, *H*_8_), 4.61 (br, s, 1H, N*H*), 3.72 (dd, *J* = 14.1, 4.4 Hz, 1H, *H*_7_), 2.98 (d, *J* = 14.1 Hz, 1H, *H*_7’_).

^19^F NMR (376 MHz, CDCl_3_) : δ (ppm) = -62.6.

^13^C NMR (101 MHz, ) δ 149.85 (s, *C*_9_), 145.7 (s, *C*_1_), 143.3 (s, *C*_6_), 129.5 (s, *C*H_4_), 127.6 (s, *C*H_3_), 126.9 (s, *C*H_10_), 125.1 (q, *Jc-^19^_F_* = 263 Hz, *C*F_3-13_), 123.9 (s, *C*H_2_), 122.4 (s, *C*H_5_), 119.7 (q, *Jc-^19^_F_* = 33.2 Hz, *C*_12_), 112.5 (s, *C*H_11_), 54.0 (s, *C*H_8_), 40.3 (s, *C*H2_7-7’_).

NMR data are in perfect agreement with the literature.***^9^***

NMR spectra of **4a**: Figures S28-32.

HRMS of **4a** (ESI, negative mode) calcd for [M-H]^-^ [C_15_H_11_F_3_N]^-^ *m/z* 262.0849; found 262.0846

***1-N-[p-(ethylacetate)phenyl]benzocyclobutene 4b***

Synthesis details: **3** (0.5 g, 4.2 mmol), 4-(ethylacetate)aniline (1.4 g, 8.5 mmol) and NaBH(OAc)_3_ (1.8 g, 8.5 mmol) were reacted following the general procedure. The product **4b** was isolated as a white solid after recrystallisation from pentane/ethylacetate (98:2) to give 0.67 g (59%). White solid, melting point 110 °C (DSC, Figure S8).

^1^H NMR (400 MHz, CDCl_3_) : δ (ppm) = 7.93 (d, *J* = 8.5 Hz, 2H, *H_11_*), 7.33 (t, *J* = 7.2 Hz, 1H, *H_ar_*), 7.26 (m, *H_ar_*), 7.17 (d, *J* = 7.2 Hz, 1H, *H_ar_*), 6.71 (d, *J* = 8.1 Hz, 2H, *H_10_*), 5.12 (dd, *J* = 4.5, 2.0 Hz, 1H, *H_8_*), 4.33 (q, *J* = 7.1 Hz, 2H, *H_14_*), 3.72 (dd, *J* = 14.2, 4.5 Hz, 1H, *H_7_*), 3.02 (d, *J* = 14.3 Hz, 1H, *H_7’_*), 1.37 (t, *J* = 7.1 Hz, 3H, *H_15_*).

^13^C NMR (101 MHz, CDCl_3_) : δ (ppm) = 166.8 (*C_9_*), 150.35 (*C_13_*), 145.2 (*C*_ar_), 143.2 (*C*_ar_), 131.65 (*C_12_*), 129.5 (*C*H_ar_), 127.6 (*C*H_ar_), 123.82 (*C*H_ar_), 122.5 (*C*H_ar_), 120.0 (*C*_ar_), 112.6 (*C_11_*), 60.4 (*C_14_*), 54.1 (*C_8_*), 40.1 (*C_7_*), 14.5 (*C_15_*).NMR spectra of **4b**: Figures S33-37.

HRMS (ESI) calcd for [M+H]^+^ [C_17_H_18_NO_2_]^+^: *m/z* 268.1333; found 268.1332

***1-N-[p-(methoxy)phenyl]benzocyclobutene 4c***

Synthesis details: **3** (1.5 g, 12.6 mmol), 4-(methoxy)aniline (1.7 g, 13.9 mmol) and NaBH(OAc)_3_ (5.3 g, 25 mmol) were reacted following the general procedure. The product **4c** was isolated as a pale yellow solid after silica gel chromatography purification using pentane/ethylacetate 98:2 (1.16 g, 41%). Pale yellow solid, melting point 70 °C (DSC, Figure S9).

^1^H NMR (400 MHz, CDCl3) δ 7.29 (m, 1H), 7.23 (m, 2H), 7.15 (d, *J* = 7.2 Hz, 1H), 6.83 (d, *J* = 8.9 Hz, 2H), 6.71 (d, *J* = 8.9 Hz, 2H), 5.04 (dd, *J* = 4.3, 2.0 Hz, 1H), 3.77 (s, 3H), 3.66 (dd, *J* = 14.2, 4.4 Hz, 1H), 2.95 (d, *J* = 14.1 Hz, 1H).

^13^C NMR (101 MHz, CDCl_3_) δ 152.8 (s), 146.65 (s), 143.4 (s), 141.1 (s), 129.0 (s), 127.2 (s), 123.7 (s), 122.4 (s), 115.0 (s), 55. 8 (s), 55.4 (s), 40.1 (s).

HRMS (ESI) calcd for [M+H]^+^ [C_15_H_16_NO]^+^: *m/z* 226.1229; found 226.1232

NMR spectra of **4c**: Figures S38-41.

***1-N-[phenyl]benzocyclobutene 4d***

Synthesis details: **3** (0.67 g, 5.7 mmol), aniline (0.53 mL, 0.53 g, 5.7 mmol) and NaBH(OAc)_3_ (2.4 g, 11.3 mmol) were reacted following the general procedure. The crude product was purified by silica gel chromatography purification using pentane/ethyl acetate (95:5) as eluent, affording **4d** as a pale yellow oil. **Note:** The product was initially characterized by ^1^H NMR. However, upon exposure to air for 48 h, the oil darkened and was oxidized to compound **10d**. Therefore, due to its high air sensitivity, **4d** could not be isolated pure.

^1^H NMR (300 MHz, CDCl_3_) : δ (ppm) = 7.34 – 7.11 (m), 6.83 – 6.67 (m), 5.10 (s, 1H), 3.70 (dd, *J* = 14.1, 4.4 Hz, 1H), 2.98 (dd, *J* = 14.1, 2.1 Hz, 1H); (*Note:* Unidentified minor impurities are indicated with an asterisk).

^13^C NMR (75 MHz, CDCl_3_) : δ (ppm) = 147.3 (*C*_ar_), 146.5 (*C*_ar_), 143.4 (*C*_ar_), 129.4 (*C*H_ar_), 129.0 (*C*H_ar_), 127.3 (*C*H_ar_), 123.7 (*C*H_ar_), 122.3 (*C*H_ar_), 118.1 (*C*H_ar_), 113.35 (*C*H_ar_), 54.4 (*C*_8_), 40.3 (*C*_7_). (*Note*: The ^13^C NMR spectrum showed additional unidentified signals attributed to partial degradation of compound **4d** during analysis).

NMR Spectra of **4d**: Figures S42-43

***General procedure for synthesis of amido-BCBs 5a-d.***

The corresponding amino-BCB derivative was dissolved in dry CH_2_Cl_2_ containing 3 Å molecular sieves (2g/g of amine). Acetyl chloride (3-5 equiv.) and K_2_CO_3_ (3-6 equiv.) were added under argon (caution : amino-BCBs are highly sensitive to O_2_ in solution), and the reaction mixture was stirred at 40 °C for 18 h. After completion, the mixture was filtered, and the filtrate was concentrated under reduced pressure. The crude residue was purified by silica gel chromatography using pentane/ethyl acetate (80:20) to afford the acetylated amino-BCB product.

***1-[p-(trifluoromethyl)phenylacetamide]benzocyclobutene 5a***

Synthesis details: Compound **4a** (0.2 g, 0.76 mmol), acetyl chloride (0.26 mL, 0.28 g, 3.7 mmol), and K_2_CO_3_ (0.42 g) were reacted following the general procedure. The crude mixture was purified by silica gel chromatography to afford **5a** as a white solid (0.18 g, 78%). Melting point 90 °C (DSC, Figure S10).

^1^H NMR (300 MHz, CDCl_3_) : δ (ppm) = 7.57 (d, J = 8.2 Hz, 2H, *H_13_*), 7.23 (d, J = 8.2 Hz, 2H, *H_12_*), 7.12 (m, 2H, H*_ar_*), 6.97 (d, *J* = 7.1 Hz, 1H, *H_ar_*), 6.97 (d, *J* = 7.1 Hz, 1H, *H_ar_*), 6.17 (br s, 1H, *H_8_*), 3.63 (dd, J = 14.5, 5.1 Hz, 1H, *H_7_*), 2.99 (dd, J = 14.5, 2.5 Hz, 1H, *H_7_’*), 1.90 (s, 3H, *H_10_*).

^13^C NMR (75 MHz, CDCl_3_) : δ (ppm) = 170.1 (*C_9_*), 144.3 (*C*_ar_), 143.3 (*C*_ar_)_,_  143.2 (*C*_ar_), 130.9 (q, *J_C-F_* = 37 Hz, *C*_14_), 130.3 (*C*_ar_), 129.8 (*C_12_*), 129.0 (*C*H_ar_), 127.3 (*C*H_ar_), 126.4 (q, *J_C-F_* = 3 Hz, *C_13_*), 130.9 (q, *J_C-F_* = 271 Hz, *C*_15_), 123.3 (*C*H_ar_), 123.1 (*C*H_ar_), 55.6 (*C_8_*), 38.0 (*C_7_*), 23.4 (*C_10_*).

^19^F NMR (376 MHz, CDCl_3_) : δ (ppm) = 62.6.

NMR spectra of **5a**: Figures S44-47.

MS (ESI) calcd for [M+H]^+^ [C_17_H_15_NOF_3_]^+^: *m/z* 306.1, found 306.3; calcd for [M+Na]^+^ [C_17_H_14_NOF_3_Na]^+^: *m/z* 328.1, found 328.2; calcd for [M+K]^+^ [C_17_H_14_NOF_3_K]^+^: *m/z* 344.1, found 344.2.

***1-[p-(ethylacetate)phenylacetamide]benzocyclobutene 5b***

Synthesis details: Compound **4b** (0.93 g, 3.5 mmol), acetyl chloride (1.2 mL, 1.3 g, 17 mmol), and K_2_CO_3_ (2.6 g) were reacted following the general procedure. After purification, the product **5b** was obtained as a white solid (0.92 g, 85%).

^1^H NMR (300 MHz, CDCl_3_) : δ (ppm) = 7.57 (d, *J* = 8.2 Hz, 2H, *H*_12_), 7.23 (d, *J* = 8.2 Hz, 2H, *H*_11_) 7.22 – 7.08 (m, 2H, *H*_ar_), 6.94 (dd, *J* = 23.2, 7.1 Hz, 2H, *H*_ar_), 6.17 (m, 1H, *H_8_*), 3.63 (dd, *J* = 14.5, 5.1 Hz, 1H, *H_7_*), 2.99 (dd, *J* = 14.5, 2.5 Hz, 1H, *H_7’_*), 1.90 (s, 3H, *H_10_*).

^13^C NMR (75 MHz, CDCl_3_) : δ (ppm) = 170.1 (*C_9_*), 166.2 (*C_15_*), 144.3 (*C*_ar_), 143.2 (*C*_ar_), 142.2 (*C*_ar_), 130.6 (*C_13_*), 130.4 (*C*_ar_), 129.3 (*C_12_*), 128.9 (*C*H_ar_), 127.2 (*C*H_ar_), 123.4 (*C*H_ar_), 123.0 (*C*H_ar_), 61.25 (*C_16_*), 55.6 (*C_8_*), 37.9 (*C_7_*), 23.3 (*C_10_*), 14.3 (*C_17_*).

NMR spectra of **5b**: Figures S48-51.

MS (ESI) calcd for [M+H]^+^ [C_19_H_20_NO_3_]^+^: *m/z* 310.1, found 310.3; calcd for [M+Na]^+^ [C_19_H_19_NO_3_Na]^+^: *m/z* 332.1; found 332.2; calcd for [M+K]^+^ [C_19_H_19_NO_3_K]^+^: *m/z* 348.1; found 348.2.

***1-[p-(methoxy)phenylacetamide]benzocyclobutene 5c***

Synthesis details: Compound **4c** (0.50 g, 2.2 mmol), acetyl chloride (0.48 mL, 0.5 g, 6.4 mmol), and K_2_CO_3_ (0.9 g) were reacted according to the general procedure. After purification, the product **5c** was obtained as a white solid (0.27 g, 46%). Melting point 85 °C (DSC, Figure S12).

^1^H NMR (400 MHz, CDCl_3_) δ 7.15 (t, *J* = 7.2 Hz, 1H, *H_4_*), 7.10 (t, *J* = 7.2 Hz, 1H, *H_3_*), 6.99 (d, *J* = 9 Hz, 2H, *H_13_*), 6.95 (d, *J* = 7.2 Hz, 1H, *H_2_*), 6.93 (d, *J* = 7.2 Hz, 1H, *H_5_*), 6.78 (d, *J* = 8.8 Hz, 2H, *H_12_*), 6.16 (dd, *J* = 4.9, 2.6 Hz, 1H, *H_8_*), 3.75 (s, 3H, *H_15_*), 3.58 (dd, *J* = 14.3, 5.1 Hz, 1H, *H_7_*), 3.01 (dd, *J* = 14.4, 2.4 Hz, 1H, *H_7’_*), 1.86 (s, 3H, *H_10_*).

^13^C NMR (101 MHz, CDCl_3_) δ 171.3 (*C_9_*), 159.2 (*C_14_*), 145.1 (s, *C_1_*), 142.5 (s, *C_6_*), 132.7 (s, *C_11_*), 130.4 (s, *C_13_*), 128.7 (s, *C_4_*), 127.1 (s, *C_3_*), 123.5 (s, *C_2_*), 122.9 (s, *C_5_*), 114.4 (s, *C_12_*), 55.5 (s, *C_8_*), 55.4 (s, *C_15_*), 37.8 (s, *C_7_*), 23.4 (s, *C_10_*).

NMR spectra of **5c**: Figures S52-55.

***1-[p-(ethylacetate)phenylacetamide]benzocyclobutene 5d***

Synthesis details: Crude **4d** (used immediately after synthesis, 0.5 g, 2.5 mmol), acetyl chloride (1.0 mL, 1.1 g, 14 mmol), and K_2_CO_3_ (1.4 g) were reacted according to the general procedure. After purification, the product **5d** was isolated as a white solid (0.17 g, 29%). Melting point 60 °C (DSC, Figure S13).

^1^H NMR (300 MHz, CDCl_3_) : δ (ppm) = 7.29 (m, 3H, *H*_ar_), 7.12 – 7.04 (m, 3H, *H*_ar_), 6.96 (d, *J* = 7.1 Hz, 1H, *H*_ar_), 6.89 (d, *J* = 7.1 Hz, 1H, *H*_ar_), 6.16 (m, 1H, *H_8_*), 3.61 (dd, *J* = 14.5, 5.1 Hz, 1H, *H_7_*), 3.03 (dd, *J* = 14.7, 2.6 Hz, 1H, *H_7_*_’_), 1.87 (s, 3H, *H_10_*).

^13^C NMR (75 MHz, CDCl_3_) : δ (ppm) = 170.75 (*C_9_*), 144.8 (*C*_ar_), 142.4 (*C*_ar_), 140.0 (*C*_ar_), 129.3 (*C*H_ar_), 129.2 (*C*H_ar_), 128.6 (*C*H_ar_), 128.25 (*C*H_ar_), 127.0 (*C*H_ar_), 123.4 (*C*H_ar_), 122.8 (*C*H_ar_), 55.6 (*C_8_*), 37.77 (*C_7_*), 23.3 (*C_10_*).

NMR spectra of 5d: Figures S56-59.

HRMS (ESI) calcd for [M+H]^+^ [C_16_H_16_NO]^+^: *m/z* 238.1226; found 238.1226

**Synthesis of BCB-N(C(O)OMe)-Ph-CO_2_Et 6**

Amino-BCB **4b** (0.21 g, 0.77 mmol) was dissolved in dry CH_2_Cl_2_ containing 3 Å molecular sieves (0.4 g). Methyl chloroformate (6 equiv., 0.35 mL, 0.42 g, 4.5 mmol) and K_2_CO_3_ (5.5 equiv., 0.53 g) were added under argon (caution : amino-BCBs are highly sensitive to O_2_ in solution). The reaction mixture was stirred at 40 °C for 18 h. After completion, the mixture was filtered, and the filtrate was concentrated under reduced pressure. The crude residue was purified by silica gel chromatography using pentane-ethyl acetate (85:15) to afford **6** isolated as a white solid (0.15 g, 59%). Melting point 90 °C (DSC, Figure S14).

NMR spectra of **6** : Figures S60-63

^1^H NMR (300 MHz, CDCl_3_) : δ (ppm) = 7.94 (d, *J* = 8.7 Hz, 2H, *H_13_*), 7.23-7.09 (m, 2H, *H*_ar_), 7.14 (d, *J* = 8.7 Hz, 2H, *H_12_*), 7.03-6.93 (m, 2H, *H*_ar_), 5.86 (dd, *J* = 5.0, 2.6 Hz, 1H, *H_8_*), 4.34 (q, *J* = 7.1 Hz, 2H, *H_16_*), 3.72 (s, 3H, *H_10_*), 3.59 (dd, *J* = 14.4, 5.2 Hz, 1H, *H_7_*), 3.08 (dd, *J* = 14.3, 2.7 Hz, 1H, *H_7_*_’_), 1.36 (t, *J* = 7.1 Hz, 3H, *H_17_*). ^13^C NMR (75 MHz, CDCl_3_) : δ (ppm) = 165.95 (*C_15_*), 155.8 (*C_9_*), 144.6 (*C*_ar_), 143.1 (*C*_ar_), 141.9 (*C*_ar_), 130.05 (*C_13_*), 129.4 (*C*_ar_), 129.0 (*C*H_ar_), 128.7 (*C_12_*), 127.2 (*C*H_ar_), 123.3 (*C*H_ar_), 123.1 (*C*H_ar_), 61.1 (*C_16_*), 57.3 (*C_8_*), 53.1 (*C_10_*), 38.0 (C_7_), 14.3 (*C_17_*).

HRMS (ESI) calcd for [M+H]^+^ [C_19_H_20_NO_4_]^+^: *m/z* 326.1387; found 326.1402

***General procedure for synthesis of Diels-Alder adducts 7a-f***

In a sealed vial equipped with a septum, 0.050 g of BCB and 4 equiv. of maleic anhydride were dissolved in 1 mL of dry *tert*-butylbenzene and degassed under argon (caution : amino-BCBs are highly sensitive to O_2_ in solution). The mixture was stirred at the corresponding ROT for 24 h. After cooling to room temperature, toluene was added to precipitate the excess maleic anhydride, and the mixture was filtered. The filtrate was concentrated under reduced pressure, and the crude product was purified by silica gel chromatography to afford the corresponding adduct.

***4-methoxy-3a,4,9,9a-tetrahydronaphtho[2,3-c]furan-1,3-dione 7a***

The reaction was performed according to the general procedure. The product **7a** was obtained as a white solid (0.062 g, 72%), melting point 180 °C.

^1^H NMR (300 MHz, CDCl_3_) : δ (ppm) = 7.38-7.29 (m, 2H, *H*_ar_), 7.25-7.21 (m, 2H, *H*_ar_), 4.70 (d, *J* = 3.7 Hz, 1H, *H_7_*), 3.45 (ddd, *J* = 10.6, 9.9, 9.0 Hz, 1H, *H_11_*) 3.36-3.14 (m, 2H, *H_8_* and *H_12_*), 3.10 (s, 3H, *H_13_*).

^13^C NMR (75 MHz, CDCl_3_) : δ (ppm) = 172.8 (*C_9_* or *C_10_*), 169.9 (*C_9_* or *C_10_*), 134.4 (*C_5_* or *C_6_*), 132.4 (*C_5_* or *C_6_*), 128.75 (*C*H_ar_), 127.8 (*C*H_ar_), 127.7 (*C*H_ar_), 125.8 (*C*H_ar_), 76.4 (*C_7_*), 55.0 (*C_13_*), 46.5 (*C_8_*), 37.1 (*C_11_*), 26.0 (*C_12_*).

NMR spectra of **7a**: Figures S64-67.

HRMS (ESI) m/z [M+Na]^+^ [C_13_H_12_NaO_4_]^+^: *m/z* 255.0628; found 255.0631.

***4-isobutoxy-3a,4,9,9a-tetrahydronaphtho[2,3-c]furan-1,3-dione 7b***

The reaction was performed according to the general procedure. The product **7b** was obtained as a white solid (0.034 g, 44%), melting point 190 °C.

^1^H NMR (300 MHz, CDCl_3_) : δ (ppm) = 7.39-7.32 (m, 1H, *H*_ar_), 7.30-7.22 (m, 3H, *H*_ar_), 4.80 (d, *J* = 3.5 Hz, 1H, *H_7_*), 3.47 (ddd, *J* = 10.4, 9.5, 8.5 Hz, 1H, *H_11_*), 3.30 (ddd, *J* = 15.0, 8.4, 0.9 Hz, 1H, *H_12_*), 3.26 (dd, *J* = 10.5, 3.6 Hz, *H_12’_*), 3.21 (dd, *J* = 15.0, 9.6 Hz, 1H, *H_8_*), 1.70 (app sep, *J* = 6.6 Hz, 1H, *H_14_*), 0.77 (d, *J* = 6.7 Hz, 3H, *H_15_*), 0.74 (d, *J* = 6.7 Hz, 3H, *H_15’_*).

^13^C NMR (75 MHz, CDCl_3_) : δ (ppm) 174.1 (*C_9_* or *C_10_*), 171.2 (*C_9_* or *C_10_*), 135.5 (s, *C*_ar_), 134.1 (s, *C*_ar_), 129.8 (s, *C*H_ar_), 128.9 (*C*H_ar_), 128.6 (s, *C*H_ar_), 126.9 (s, *C*H_ar_), 76.3 (s, *C_7_*), 75.3 (s, *C_13_*), 47.8 (s, *C_8_*), 38.2 (s, *C_11_*), 28.35 (s, *C_14_*), 26.8 (s, *C_12_*), 19.22 (s, *H_15_*)19.20 (s, *C_15’_*).

NMR spectra of 7b: Figures S68-71.

HRMS (ESI) m/z [M+H]^+^ [C_16_H_19_O_4_]^+^: *m/z* 275.1278; found 275.1280.

***4-((trimethylsilyl)oxy)-3a,4,9,9a-tetrahydronaphtho[2,3-c]furan-1,3- dione 7c***

The reaction was performed according to the general procedure. The product **7c** was obtained as a white solid (0.052 g, 69%), melting point 200 °C.

^1^H NMR (400 MHz, CDCl_3_) δ 7.42-7.29 (m, 1H, *H_ar_*), 7.26-7.20 (m, 3H, *H_ar_*), 5.22 (d, *J* = 3.2 Hz, 1H, *H_7_*), 3.52-3.33 (m, 2H, overlapped signals of *H_11_* and *H_12_*), 3.25-3.15 (m, 2H, overlapped signals of *H_8_* and *H_12’_*), -0.05 (s, 9H, *H_13_*).

^13^C NMR (101 MHz, CDCl_3_) δ 174.3 (s, *C_10_*), 171.4 (s, *C_9_*), 136.7 (s, C*_6_*), 135.5 (s, C*_5_*), 129.5 (s, *C*H_ar_), 128.9 (s, *C*H_ar_), 127.15 (s, *C*H_ar_), 127.1 (s, *C*H_ar_), 70.5 (s, *C_7_*), 49.4 (s, *C_8_*), 38.1 (s, *C_11_*), 26.9 (s, *C_12_*), -0.2 (s, *C_13_*).

NMR spectra of 7c: Figures S72-73.

HRMS (ESI) calcd for m/z [M+H]^+^ [ C_15_H_19_O_4_Si]^+^ 291.1053; found 291.1049.

***4-((4-(trifluoromethyl)phenyl)amino)-3a,4,9,9a-tetrahydronaphtho[2,3-c]furan-1,3-dione 7d***

The reaction was performed according to the general procedure. The product **7d** was obtained as a white solid (0.036 g, 53%). The DSC analysis of the compound does not show a melting point, but degradation occurs starting at 160 °C.

^1^H NMR (400 MHz, CDCl_3_ + 20vol%DMSO-D_6_, ref CHCl_3_ 7.26 ppm) : δ (ppm) = 7.32 (d, *J* = 8.5 Hz, 2H, *H*_14_), 7.10 (d, *J* = 8.5 Hz, 2H, *H*_15_), 6.91 (d, *J* = 7.5 Hz, 1H, *H*_1_), 6.81 (t, *J* = 7.5 Hz, 1H, *H*_3_), 6.74 (d, *J* = 7.4 Hz, 1H, *H*_4_), 6.69 (t, *J* = 7.3 Hz, 1H, *H*_2_), 4.89 (s, 1H, *H*_7_), 2.92 (br s, 1H, *H*_11_), 2.87 – 2.74 (m, 2H, *H*_12_), 2.70 (s, 1H, *H*_8_).

^13^C NMR (101 MHz, CDCl_3_ + 20vol%DMSO-D_6_, ref CDCl_3_ 77.16 ppm) : δ (ppm) = 174.45 (s, *C*_9_), 172.6 (s, *C*_10_), 167.3 (s, DMSO), 141.5 (s, *C*_13_), 136.7 (s, *C*_5_ or *C*_6_), 133.5 (s, *C*_5_ or *C*_6_), 130.4 (s, *C*_4_), 129.35 (s, *C*_3_), 127.3 (s, *C*_1_), 126.2 (m, overlapped signals of *C*_2_ and *C*_15_), 125.8 (q, *J* = 33 Hz, *C*_16_), 124.2 (q, *J* = 270 Hz, *C*_17_), 120.1 (s, *C*_14_), 62.7 (s, *C*_7_), 48.9 (s, *C*_8_), 43.95 (s, *C*_11_), 40.3 (dp, *J* = 42.0, 21.0 Hz, DMSO), 30.7 (s, *C*H_2-12_).

^19^F NMR (376 MHz, CDCl_3_ + 20vol%DMSO-D_6_) : δ (ppm) = 61.9.

NMR spectra of **7d**: Figures S74-78.

HRMS (ESI) calcd for m/z [M+H]^+^ [C_19_H_15_F_3_NO_3_]^+^ 362.1004; found 362.0997.

***Ethyl 4-((1,3-dioxo-1,3,3a,4,9,9a-hexahydronaphtho [2,3-c]furan-4-yl)amino)benzoate* 7e**

The reaction was performed according to the general procedure. The product **7e** was obtained as a white solid (0.039 g, 57%). The DSC analysis of the compound does not show a melting point, but degradation occurs starting at 160 °C.

^1^H NMR (400 MHz, CDCl_3_) δ 7.98 (d, *J* = 8.9 Hz, 2H, *H_15_*), 7.62 (d, *J* = 8.9 Hz, 2H, *H_14_*), 7.28-7.24 (m, overlapped signals of C*H*_ar_ with CHCl_3_), 7.20 (d, *J* = 8 Hz, 1H, *H_4_*), 7.15 (t, *J* = 8 Hz, 1H, C*H*_ar_), 5.28 (s, 1H, *H_7_*), 4.34 (q, *J* = 7.1 Hz, 2H, *H_18_*), 3.49 (d, *J* = 5.1 Hz, 1H, *H_11_*), 3.34 (d, *J* = 16.9 Hz, 1H, *H_12_*), 3.27 (dd, dd, *J* = 16.9, 5.6 Hz, 1H, *H_12’_*), 3.22 (app s, 1H, *H_8_*), 1.36 (t, *J* = 7.1 Hz, 3H, *H_19_*).

^13^C NMR (101 MHz, CDCl_3_) δ 175.4 (*C_9_* or *C_10_*), 174.0 (*C_9_* or *C_10_*), 166.2 (*C_17_*), 141.8 (*C*_16_), 136.1 (*C*_ar_), 133.2 (*C*_ar_), 130.7 (*C_15_*), 130.4 (*C*H_ar_), 129.6 (*C*H_ar_), 127.2 (*C*H_ar_), 126.8 (*C*H_ar_), 126.3 (*C*H_ar_), 120.2 (*C_14_*), 63.0 (*C_7_*), 61.1 (*C_18_*), 49.0 (*C_11_*), 43.8 (*C_8_*), 30.6 (*C_12_*), 14.44 (*C_19_*).

NMR spectra of **7e**: Figures S79-83.

HRMS (ESI) m/z calcd for [M+H]^+^ [C_21_H_20_NO_4_]^+^ 366.1341; found 366.1333.

***Ethyl 4-((1,3-dioxo-1,3,3a,4,9,9a-hexahydronaphtho [2,3-c]furan-4-yl)(methoxycarbonyl)amino)benzoate* 7f**

The reaction was performed according to the general procedure. The product **7f** was obtained as a white solid (0.038 g, 58%). The DSC analysis of the compound does not show a melting point, but degradation occurs starting at 160 °C.

^1^H NMR (300 MHz, CDCl_3_) : δ (ppm) = 8.00 (d, *j* = 9.0 Hz, 2H, *H_15_*), 7.36-7.27 (m, 2H, *H*_ar_), 7.25 – 7.15 (m, 2H, *H*_ar_), 7.08 (d, *j* = 9.0 Hz, 2H, *H_15_*), 5.69 (d, *J* = 9.2 Hz, 1H, *H_7_*), 4.38 (q, J = 7.2 Hz, 2H, *H_18_*), 3.81 (dd, *J* = 8.9, 2.5 Hz, 1H, *H_8_*), 3.59 (s, 3H, *H_21_*), 3.57 – 3.48 (m, 1H, *H_11_*), 2.92 (dd, *J* = 15.8, 8.4 Hz, 1H, *H_12_*), 2.62 (dd, *J* = 15.8, 8.4 Hz, 1H, *H_12’_*), 1.40 (t, *J* = 7.1 Hz, 3H, *H_19_*).

^13^C NMR (75 MHz, CDCl_3_) : δ (ppm) = 172.75 (*C_9_* or *C_10_*), 170.2 (*C_9_* or *C_10_*), 165.7 (*C_20_*), 134.9 (*C*_ar_), 132.85 (*C*_ar_), 130.6 (*C_15_*), 129.9 (*C*_ar_), 129.2 (*C*H_ar_), 129.1 (*C*H_ar_), 128.75 (*C_14_*), 127.7 (*C*H_ar_), 61.3 (*C_18_*), 57.5 (*C_7_*), 53.4 (*C_21_*), 43.1 (*C_11_*), 40.3 (*C_8_*), 27.1 (*C_12_*), 14.3 (*C_19_*).

NMR spectra of **7f**: Figures S84-87.

HRMS (ESI) m/z [M+NH_4_]^+^ calcd for [C_23_H_25_N_2_O_7_]^+^ 441.1656, found 441.1660.

***Synthesis of TEMPO adducts 8a-d***

***1-((2-(methoxy((2,2,6,6-tetramethylpiperidin-1-yl)oxy)methyl)benzyl)oxy)-2,2,6,6-tetramethylpiperidine 8a***

Benzocyclobutene **2a** (0.032 g, 0.24 mmol), TEMPO (0.105 g, 0.67 mmol), and *tert*-butylperoxide (0.066 g, 0.45 mmol) were dissolved in dry *t*ert-butylbenzene (0.3 mL) in a sealed vial equipped with a septum. The mixture was stirred and heated at 110 °C. Aliquots were taken at 0.5, 1, 2, 4 and 8 h and analyzed by ^1^H analysis in C_6_D_6_ to monitor the reaction progress. After 24 h, all volatiles were removed under reduced pressure, and the crude mixture was purified by silica gel chromatography (pentane-diethylether 95:5), affording as a pale yellow wax (0.052 g, 49%).

^1^H NMR (300MHz, CDCl_3_) : δ (ppm) = 7.60-7.50 (m, 2H, overlapped signals of *H_3_* and *H_6_*), 7.40-7.30 (m, 2H, overlapped signals of *H_4_* and *H_5_*), 5.88 (s, 1H, *H_7_*), 5.04 (d, *J* = 12 Hz, 1H, *H_16_*), 4.90 (d, *J* = 12 Hz, 1H, *H_16’_*), 3.06 (s, 3H, *H_8_*), 1.64 – 1.20 (m, 24H, overlapped signals of C*H_2_*_(TEMPO)_ and C*H_3_*_(TEMPO)_), 1.09 (m, 12H, overlapped signals of C*H_2_*_(TEMPO)_ and C*H_3_*_(TEMPO)_).

^13^C NMR (101 MHz, CDCl_3_) : δ (ppm) = 136.9 (s, *C*_ar_), 135.3 (s, *C*_ar_), 128.0 (s, *C*H_ar_), 127.5 (s, *C*H_ar_), 127.4 (s, *C*H_ar_), 126.2 (s, *C*H_ar_), 106.2 (s, *C_7_*), 75.9 (s, *C_16_*), 60.7 (s, *C*_(TEMPO)_), 60.0 (*C*_(TEMPO)_), 59.5 (*C*_(TEMPO)_), 51.1 (s, *C_8_*), 40.7 (s, *C*H_2(TEMPO)_), 40.2 (s, *C*H_2(TEMPO)_), 40.0 (s, *C*H_2(TEMPO)_), 39.95 (s, *C*H_2(TEMPO)_), 33.9 (s, *C*H_3(TEMPO)_), 33.2 (s, *C*H_3(TEMPO)_), 33.1 (s, *C*H_3(TEMPO)_), 21.0 (s, *C*H_3(TEMPO)_), 20.55 s, *C*H_3(TEMPO)_), 20.5 (s, *C*H_3(TEMPO)_), 17.35 (s, C*H_2_*_(TEMPO)_), 17.3 (s, -C*H_2_*_(TEMPO)_).

NMR spectra of **8a**: Figures S88-92. The reaction of **2a** and TEMPO with time at 110 °C to afford **8a** is presented in Figure S89.

HRMS (ESI) m/z [M+H]^+^ calcd for [C_27_H_47_N_2_O_3_]^+^ 447.3581, found 447.3583.

***General procedure for the synthesis of TEMPO adducts 8b-d***

In a sealed vial equipped with a septum, 0.050 g of BCB (**2b**, **5b** or **6**) and 3 equiv. of TEMPO were dissolved in 1 mL of dry *tert*-butylbenzene (tBbenz) and degassed under argon. The mixture was stirred at the corresponding ROT for 24 h. After cooling to room temperature, all volatiles were removed under reduced pressure, and the crude product was purified by silica gel chromatography using pentane-diethylether 95:5 (8a, 49% and **8b**,89%) or pentane-ethylacetate 95:5 (**8c,** 51% and **8d,** 81%).

The thermal analysis of adducts **8a-d** shows the liberation of TEMPO via homoleptic cleavage.^10^

*Note: When the same procedure was applied to benzocyclobutene derivatives* ***4a-c*** *the reactions led to the formation of benzaldehyde alkoxyamine derivatives* ***9****, rather than the expected TEMPO-adduct.*

***1-((2-(isobutoxy((2,2,6,6-tetramethylpiperidin-1-yl)oxy) methyl) benzyl) oxy) -2,2,6,6-tetramethylpiperidine 8b***

The reaction was carried out according to the general procedure using benzocyclobutene **2b**. After purification, the product **8b** was obtained as a colorless wax (0.123 g, 89%).

^1^H NMR (300 MHz, CDCl_3_) : δ (ppm) = 7.61 (d, *J* = 6.9 Hz, 1H, *H*_ar_), 7.46 (dd, *J* = 7.5, 1.4 Hz, 1H, *H*_ar_), 7.31 (td, *J* = 7.5, 1.5 Hz, 1H, *H*_ar_), 7.22 (td, *J* = 7.5, 1.5 Hz, 1H, *H*_ar_), 5.82 (s, 1H, *H_7_*), 5.03 (dd, *J* = 45.7, 13.2 Hz, 2H, *H_18_*), 3.09 (dd, *J* = 8.8, 6.1 Hz, 1H, *H_8_*), 2.86 (dd, *J* = 8.8, 7.2 Hz, 1H, *H_8’_*), 1.69 (app sep, *J* = 6.7 Hz, 1H, *H_9_*), 1.55-1.39 (br m, 10H, -C*H_2_*_(TEMPO)_), 1.40 – 1.26 (br m, 5H), 1.24 – 1.10 (m, 15H, C*H_3_*_(TEMPO)_), 1.05 (s, 3H, C*H_3_*_(TEMPO)_), 0.90 (s, 3H, C*H_3_*_(TEMPO)_), 0.78 (s, 3H, *H_10_*), 0.76 (s, 3H, *H_10’_*).

^13^C NMR (75 MHz, CDCl_3_) : δ (ppm) = 136.85 (*C*_ar_), 135.9 (*C*_ar_) 127.7 (*C*H_ar_), 127.4 (*C*H_ar_), 127.1 (*C*H_ar_), 125.8 (*C*H_ar_), 106.8 (*C_7_*), 75.7 (*C_18_*), 70.8 (*C_8_*), 60.6 (*C*_(TEMPO)_), 59.9 (*C*H_2(TEMPO)_), 59.6 (*C*_(TEMPO)_), 40.7 (*C*H_2(TEMPO)_), 40.2 (*C*H_2(TEMPO)_), 40.0 (*C*H_2(TEMPO)_), 34.1 (*C*H_3(TEMPO)_), 30.3 (*C*H_3(TEMPO)_), 30.2 (*C*H_3(TEMPO)_), 30.1 (*C*H_3(TEMPO)_), 26.8 (*C_9_*), 21.0 (*C*H_3(TEMPO)_), 20.5 (br s, *C*H_3(TEMPO)_), 19.75 (*C_10_*), 19.7 (*C_10’_*), 17.4 (*C*H_2(TEMPO)_), 17.3 (*C*H_2(TEMPO)_).

NMR spectra of **8b**: Figures S93-95.

HRMS (ESI) m/z [M+H]^+^ calcd for [C_30_H_53_N_2_O_3_]^+^ 488.4051, found 489.4054.

***ethyl 4-((methoxycarbonyl)(((2,2,6,6-tetramethylpiperidin-1-yl)oxy) (2-(((2,2,6,6- tetramethylpiperidin-1-yl) oxy) methyl) phenyl) methyl) amino) benzoate 8c***

The reaction was performed according to the general procedure using benzocyclobutene **5b**. After purification, the product **8c** was obtained as a white solid (0.049 g, 49%).

^1^H NMR (300 MHz, CDCl_3_) : δ (ppm) = 7.86 (br s, *H_12_*), 7.54 (d, *J* = 7.7 Hz, 1H, *H_3_*), 7.49 (s, 1H, *H_7_*), 7.10 (t, *J* = 7.5 Hz, 1H, *H_4_*), 6.91 (d, *J* = 7.5 Hz, 1H, *H_6_* or *H_5_*), 6.82 (t, *J* = 7.3 Hz, 1H, *H_6_* or *H_5_*), 5.04 (d, *J* = 13.9 Hz, 1H, *H_24_*), 4.81 (d, *J* = 13.8 Hz, 1H, *H_24’_*), 4.34 (q, J = 7.1 Hz, 2H, *H_15_*), 2.04 (s, 3H, C*H_3_*_(TEMPO)_), 1.77 (s, 3H, C*H_3_*_(TEMPO)_), 1.66 (s, 3H, C*H_3_*_(TEMPO)_), 1.41 – 1.15 (m, 26H, C*H_2_*_(TEMPO),_ C*H_3_*_(TEMPO),_ *H_9_*, *H_16_*), 1.02 (s, 3H, C*H_3_*_(TEMPO)_), 0.84 (s, 3H, C*H_3_*_(TEMPO)_).

^13^C NMR (101 MHz, CDCl_3_) : δ (ppm) = 168.0 (*C_8_*), 164.9 (*C_14_*), 143.1 (*C*_ar_), 134.5 (*C*_ar_), 134.4 (*C*_ar_), 128.9 (*C_11_-C_12_*), 128.6 (*C*_ar_), 126.8 (*C*H_ar_), 126.2 (*C*H_ar_), 125.0 (*C*H_ar_), 124.7 (*C*H_ar_), 85.8 (*C_7_*), 74.0 (*C_24_*), 60.1 (*C_15_*), 39.3 (*C*_(TEMPO)_), 39.1 (*C*_(TEMPO)_), 38.7 (*C*H_2(TEMPO)_), 32.3 (*C*H_3(TEMPO)_), 31.9 (*C*H_3(TEMPO)_), 31.6 (*C*H_2(TEMPO)_), 22.3 (*C_9_*), 19.65 (*C*H_3(TEMPO)_), 19.45 (*C*H_3(TEMPO)_), 19.3 (*C*H_2(TEMPO)_), 16.2 (*C*H_2(TEMPO)_), 16.0 (*C*H_2(TEMPO)_), 13.25 (*C_15_*).

NMR spectra of **8c**: Figures S96-99.

HRMS (ESI) m/z [M+H]^+^ calcd for [C_37_H_56_N_3_O_5_]^+^ 622.4214, found 622.4219.

***Ethyl 4-(N-(((2,2,6,6-tetramethylpiperidin-1-yl) oxy) (2- (((2,2,6,6-tetramethylpiperidin-1-yl) oxy) methyl) phenyl) methyl) acetamido) benzoate 8d***

The reaction was carried out according to the general procedure using benzocyclobutene **6**. After purification, the product **8d** was obtained as a white solid (0.079 g, 81%).

^1^H NMR (300 MHz, C6D6) : δ (ppm) = 7.95 (d, J = 8.6 Hz, 2H, *H_12_*), 7.78 (d, *J* = 7.2 Hz, 1H, *H*_ar_), 7.65 (s, 1H, *H_7_*), 7.30 (d, *J* = 6.1 Hz, 3H, overlapped signals of *H_11_* and *H_ar_*), 6.97 (t, *J* = 7.4 Hz, 1H, *H_ar_*), 6.78 (t, *J* = 7.4 Hz, 1H, *H_ar_*), 5.46 (d, *J* = 13.4 Hz, 1H, *H_24_*), 5.26 (d, *J* = 13.4 Hz, 1H, *H_24’_*) 4.00 (q, J = 7.0 Hz, 2H, *H_15_*), 3.36 (s, 3H, *H_9_*), 1.65-1.15 (m, 12H, C*H_2_*_(TEMPO),_ C*H_3_*_(TEMPO)_), 1.07 (br s, C*H_3_*_(TEMPO)_), 1.00 (br s, C*H_3_*_(TEMPO)_), 0.94 (t, J = 7.5 Hz, 3H, *H_16_*).
^13^C NMR (75 MHz, C6D6) : δ (ppm) = 165.2 (*C_14_*), 155.3 (*C_8_*), 143.0 (*C*_ar_), 135.5 (*C*_ar_), 135.3 (*C*_ar_), 129.6 (*C_12_*), 129.0 (*C*_ar_), 128.5 (*C_11_*), 127.6 (*C*H_ar_), 127.5 (*C*H_ar_), 126.2 (*C*H_ar_), 125.8 (*C*H_ar_), 90.5 (*C_7_*), 75.7 (*C_24_*), 59.8 (*C_15_*), 52.5 (*C_9_*), 40.3 (*C_(_*_TEMPO)_), 39.9 (*C*_(TEMPO)_), 39.7 (*C*H_2(TEMPO)_), 33.1 (*C*H_3(TEMPO)_), 32.8 (*C*H_3(TEMPO)_), 32.5 (*C*H_3(TEMPO)_), 20.5 (*C*H_3(TEMPO)_), 20.3 (*C*H_3(TEMPO)_), 20.1 (*C*H_3(TEMPO)_), 17.1 (*C*H_2(TEMPO)_), 16.9 (*C*H_2(TEMPO)_), 13.8 (*C_16_*).

Spectra of **8d**: Figures S100-104.

Kinetic Experiment of the reaction of **6** and 3 equiv. of TEMPO at 105 °C: Aliquots were withdrawn at 0.5, 1, 2, 4 and 24 h and analyzed by ^1^H NMR spectroscopy in C_6_D_6_ to monitor the reaction progress. The corresponding spectra are shown in Figure S100.

HRMS (ESI) m/z [M+H]^+^ calcd for [C_37_H_56_N_3_O_6_]^+^ 638.4164, found 638.4180

**Synthesis of benzaldehyde-TEMPO alkoxyamine 9**

The thermal activation of amino-BCBs **4a-c** in presence of TEMPO did not lead to the expected di-TEMPO-alkoxyamine, while we used dried over NaH and freshly distilled tBenz, inert atmosphere and tightly sealed tubes. Instead, an unexpected benzaldehyde-TEMPO alkoxyamine **9** (Fig. 2) was isolated. We hypothesize that the di-TEMPO adduct is initially formed but undergoes rapid decomposition due to the intrinsic weakness of N-C-O bond. This instability is likely exacerbated by the strong accessibility of the N lone pair, which facilitates successive hydrolysis reactions in the presence of water traces, despite rigorous anhydrous conditions (molecular sieves, anhydrous solvents, argon atmosphere). These hydrolytic pathways would result in the elimination of an amine, ~~a~~ TEMPO-H (1-Hydroxy-2,2,6,6-tetramethylpiperidine) and ultimately formation of **9.**

NMR spectra of **9**: Figures S104-107

^1^H NMR (300 MHz, CDCl_3_) δ 10.27 (s, 1H, C*H*O), 7.87 (dd, *J* = 7.6, 1.3 Hz, 1H, *H*_ar_), 7.69 (d, *J* = 7.4 Hz, 1H, *H*_ar_), 7.60 (td, *J* = 7.5, 1.4 Hz, 1H, *H*_ar_), 7.45 (br t, *J* = 7.5 Hz, 1H, *H*_ar_), 5.24 (s, 2H, C*H_2_*-TEMPO), 1.50 (br m, 4H, C*H_2_*_(TEMPO)_), 1.37 (br m, C*H_2_*_(TEMPO)_), 1.21 (s, C*H_3_*_(TEMPO)_), 1.15 (s, C*H_3_*_(TEMPO)_).

^13^C NMR (75 MHz, CDCl_3_) δ 192.65 (s, *C*HO), 141.2 (s, *C*_ar_), 133.9 (s, *C*H_ar_), 133.45 (s, *C*_ar_), 131.4 (s, *C*H_ar_), 128.3 (s, *C*H_ar_), 127.5 (s, *C*H_ar_), 76.3 (s, *C*H_2_-TEMPO), 60.1 (s, *C*_(TEMPO)_), 39.8 (s, *C*H_2(TEMPO)_), 33.2 (s, *C*H_3(TEMPO)_), 20.5 (s, *C*H_3(TEMPO)_), 17.2 (s, *C*H_2(TEMPO)_).

NMR spectra of **9**: Figures S105-108.

Scheme S1. **Proposed mechanism for benzaldehyde-TEMPO adduct 9 synthesis.**

***General procedure for synthesis of O_2_ adducts* 10a-d**

Synthesis of **10a** and **10b**: in a sealed vial equipped with a septum, benzocyclobutene **4a** or **4b** (0.070 g) was dissolved in 3 mL of dry CH_2_Cl_2_. The vial was purged with O_2_ and the solution was stirred at room temperature for 2 or 10 days, respectively (Figures S110 and S115, respectively). After completion, the solvent was removed under reduced pressure, and the crude product was purified by silica gel chromatography using pentane-CH_2_Cl_2_ (4:1) for **10a** or pentane-ethylacetate (10:1) for **10b**, respectively.

Synthesis of **10c** and **10d**: the direct exposure to air of **4c** and **4d** in the bulk state (without solvent) at room temperature for 24 h led to the formation of O_2_ adducts **10c** and **10d**, respectively. Compound **10c** was purified by silica gel chromatography using pentane-ethylacetate (80:20).

**N-(4-(trifluoromethyl)phenyl)-1,4-dihydrobenzo[d][1,2]dioxin-1-amine 10a**

The reaction of **4a** with molecular oxygen in CH_2_Cl_2_ for 2 days under the general procedure afforded **10a** as a pale yellow wax after purification (0.069 g, 87%). Aliquots were withdrawn during the reaction and analyzed by ^1^H NMR spectroscopy in CDCl_3_ to monitor the reaction progress. The corresponding spectra are shown in Figure S110.

^1^H NMR (400 MHz, CDCl_3_) : δ (ppm) = 7.48 (d, *J* = 8.4 Hz, 2H, *H_10_*), 7.45 – 7.30 (m, 3H, *H*_ar_), 7.17 (dd, J = 8.2, 6.8 Hz, 1H, *H*_2_), 6.89 (d, *J* = 8.5 Hz, 2H, *H_11_*), 6.32 (d, *J* = 8.4 Hz, 1H, *H_8_*), 5.32 (d, *J* = 16 Hz, 1H, *H_7_*), 5.17 (d, *J* = 8.4 Hz, 1H, N*H*), 5.00 (d, *J* = 16 Hz, 1H, *H_7’_*).

^19^F NMR (376 MHz, CDCl_3_) : δ (ppm) = -61.3.

^13^C NMR (101 MHz, CDCl_3_) : δ (ppm) = 148.2 (*C*_9_), 134.4 (*C*_6_), 132.4 (*C*_1_), 129.4 (*C*H_3_ or *C*H_4_), 128.3 (*C*H_3_ or *C*H_4_), 127.5 (overlapped signals of *C*H_10_ and *C*H_ar_), 125.5 (q, *J_C-F_* = 270 Hz, *C*_13_), 125.3 (*C*H_2_), 122.4 (q, *J_C-F_* = 33 Hz, *C*_12_), 114.6 (*C*H_11_), 83.9 (*C*H_8_), 72.4 (*C*H_7_).

NMR spectra of **10a**: Figures S109-113.

HRMS (ESI) m/z [M+H]^+^ calcd for [C_15_H_13_F_3_NO_2_]^+^ 296.0892, found 296.0869

***N-(4-(ethylbenzoate)-1,4-dihydrobenzo[d][1,2]dioxin-1-amine 10b***

The reaction of **4b** with molecular oxygen in CH_2_Cl_2_ for 10 days under the general procedure afforded **10b** as a pale yellow wax after purification (0.054 g, 69%). Aliquots were withdrawn during the reaction and analyzed by ^1^H NMR spectroscopy in CDCl_3_ to monitor the reaction progress. The corresponding spectra are shown in Figure S115.

^1^H NMR (400 MHz, CDCl_3_) : δ (ppm) = 7.94 (d, *J* = 9.1 Hz, 2H, *H_10_*), 7.39 – 7.27 (m, 3H, overlapped signals of *H*_3_, *H*_4_ and *H*_5_), 7.16, (d, *J* = 9 Hz, 1H, *H_2_*) 6.84 (d, *J* = 8.2 Hz, 2H, *H_11_*), 6.34 (d, 1H, *J* = 7 Hz*, H_8_*), 5.33 (d, *J* = 15.3 Hz, 1H, *H_7_*), 5.18 (br d, 1H, *J* = 7 Hz*,* N*H*), 5.04 (d, *J* = 15.3 Hz, 1H, *H_7’_*), 4.34 (q, *J* = 6.5 Hz, 2H, *H_14_*), 1.38 (t, *J* = 6.5 Hz, 3H, *H_15_*).

^13^C NMR (101 MHz, CDCl_3_) : δ (ppm) = 166.7 (s, *C*_13_), 148.6 (s, *C*_9_), 133.7 (s, *C*_6_), 133.7 (s, *C*_1_), 131.6 (s, *C*H_10_), 128.7 (s, *C*H_ar_), 127.6 (s, *C*H_ar_), 126.8 (s, *C*H_ar_), 124.5 (s, *C*H_2_), 113.4 (s, *C*H*_10_*), 83.1 (s, *C*H*_8_*), 71.7 (s, *C*H*_7_*), 60.6 (s, *C*H_2_-_14_), 14.4 (s, *C*H_3-15_).

NMR spectra of **10b**: Figures S114-118.

HRMS (ESI) m/z [M+H]^+^ calcd for [C_17_H_18_NO_4_]^+^ 300.1230, found 300.1232.

***N-(4-(methoxyphenyl)-1,4-dihydrobenzo[d][1,2]dioxin-1-amine 10c***

Benzocyclobutene **4c** was exposed to air in bulk at room temperature for 24 h. The resulting oxidation product **10c** was purified, affording a pale yellow wax (0.258 g, 94%).

^1^H NMR(300 MHz, CDCl_3_) : δ (ppm) = 7.44 (m, 1H, *H*_ar_), 7.33 (m, 2H, *H*_ar_), 7.14 (m, 1H, *H*_ar_), 6.84 (app s, 4H, overlapped signals of *H_10_* and *H_11_*), 6.26 (br s, 1H, *H_8_*), 5.30 (d, *J* = 15.8 Hz, 1H, *H_7_*), 5.08 (d, *J* = 15.8 Hz, 1H, *H_7’_*), 4.60 (br s, 1H, N*H*), 3.77 (s, 3H, *H_13_*).

^13^C NMR (75 MHz, CDCl_3_) : δ (ppm) = 153.8 (s, *C_12_*), 138.5 (s, *C_9_*), 133.79 (s, *C*_ar_), 132.8 (s, *C*_ar_), 128.2 (s, *C*H_ar_), 127.3 (s, *C*H_ar_), 126.8 (s, *C*H_ar_), 124.2 (s, *C*H_ar_), 116.3 (*C_11_*), 114.9 (*C_10_*), 85.2 (*C_8_*), 71.7 (*C_7_*), 55.75 (*C_13_*).

NMR spectra of **10c**: Figures S119-122.

HRMS (ESI) m/z [M+Na]^+^ calcd for [C_15_H_15_NO_3_Na]^+^ 280.0945, found 280.0944

***N-(phenyl)-1,4-dihydrobenzo[d][1,2]dioxin-1-amine 10d***

Benzocyclobutene **4d** was exposed to air in bulk at room temperature for 24 h. The reaction afforded **10d** (not purified).

^1^H NMR (300 MHz, CDCl_3_) : δ (ppm) = 7.42 (m, 1H, *H*_ar_), 7.37 – 7.13 (m, *H*_ar_), 6.88 (m, 3H, *H*_ar_), 6.34 (d, *J* = 9.8 Hz, 1H, *H_8_*), 5.31 (d, *J* = 15.3 Hz, 1H, *H_7_*), 5.09 (d, *J* = 15.3 Hz, 1H, *H_7’_*), 4.80 (d, *J* = 9.8 Hz, 1H, N*H*).

^13^C NMR (75 MHz, CDCl_3_) : δ (ppm) = 144.7 (s, *C*_9_), 133.8 (s, *C*_1_ or *C*_6_), 132.6 (s, *C*_1_ or *C*_6_), 129.4 (s, *C*H_ar_), 128.3 (s, *C*H_ar_), 127.4 (s, *C*H_ar_), 126.7 (s, *C*H_ar_), 124.3 (s, *C*H_ar_), 119.9 (s, *C*H_ar_), 114.5 (s, *C*H_ar_), 84.0 (s, *C_8_*), 71.6 (s, *C_7_*).

NMR spectra of **10d**: Figures S123-126.

***8/ NMR spectra***

Figure S15. **^1^H NMR spectrum of BCB-Br 1**

*Figure S16.* ***^1^H NMR spectrum of BCB-OMe 2a***

Figure S17. ***^1^H (a) and COSY (b) NMR spectrum of BCB-OiBu 2b***

Figure S18. ***^13^C-NRM (a) and APT NMR (b) spectra of BCB-OiBu 2b.***

Figure S19. ***HMQC (a) and HMBC (b) NMR spectra of BCB-OiBu 2b***

Figure S20. ***^1^H (a), ^13^C-APT (b), HMQC (c)*** ***NMR spectra of BCB-OAc 2c***

Figure S21. **^1^H NMR spectrum of BCB-OH 2d**

Figure S22. **^13^C NMR spectrum of BCB-OH 2d**

Figure S23. **^1^H NMR spectrum of BCB-OTMS 2e**

Figure S24. **COSY spectrum of BCB-OTMS 2e**

Figure S25. **^13^C-APT spectrum of BCB-OTMS 2e**

Figure S26. **HMQC spectrum of BCB-OTMS 2e**

Figure S27. **^1^H NMR spectrum of BCB-ketone 3**

###

Figure S28. **^1^H NMR spectrum of BCB-NHPhCF_3_ 4a**


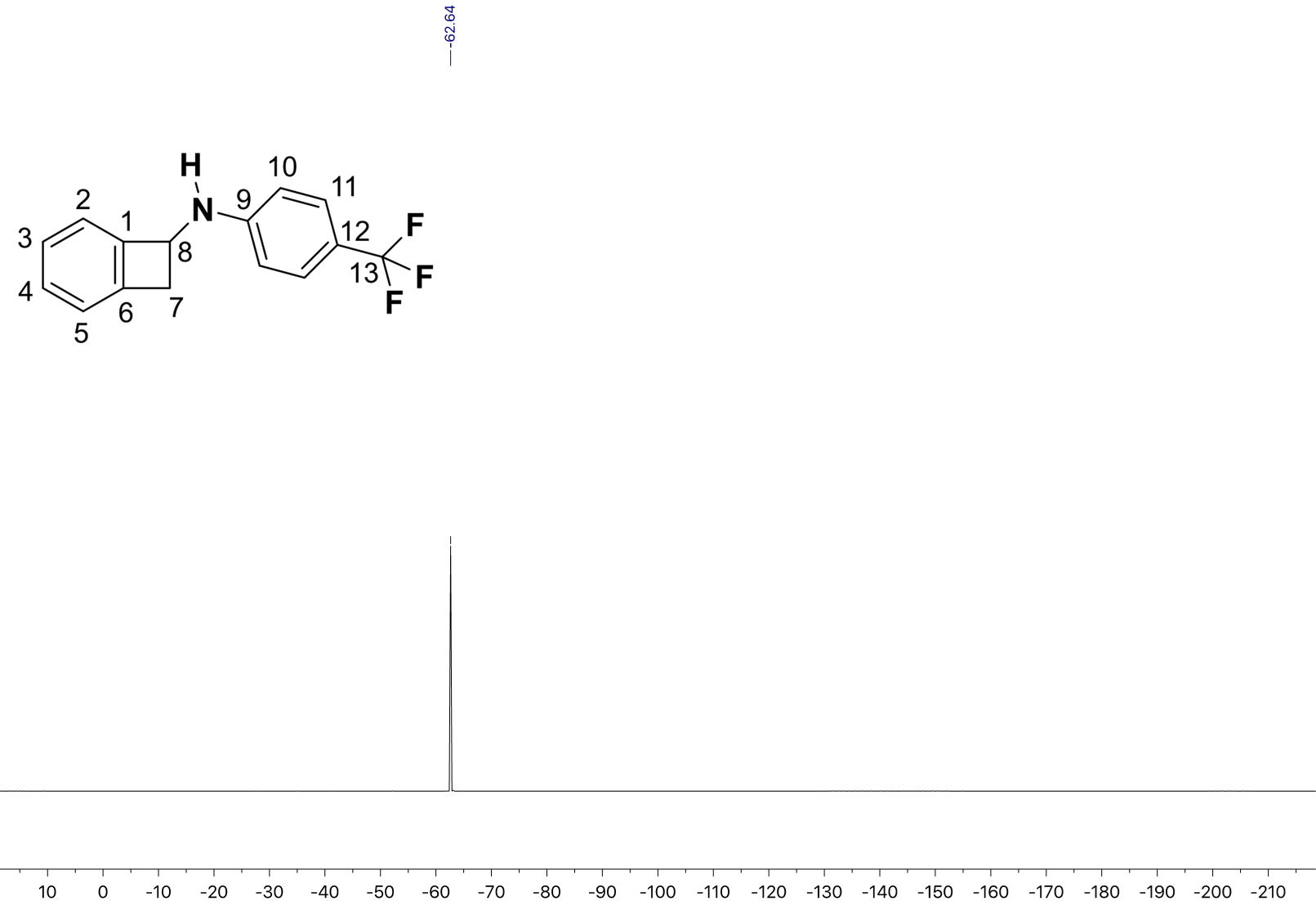


Figure S29. **^19^F NMR spectrum of BCB-NHPhCF_3_ 4a**

Figure S30. **COSY spectrum of BCB-NHPhCF_3_ 4a**

Figure S31. **^13^C-NMR spectrum of BCB-NHPhCF_3_ 4a**

Figure S32. **HMQC (a) and HMBC (b) NMR spectra of BCB-NHPhCF_3_ 4a**

Figure S33. **^1^H NMR spectrum of BCB-NHPhCO_2_Et 4b**


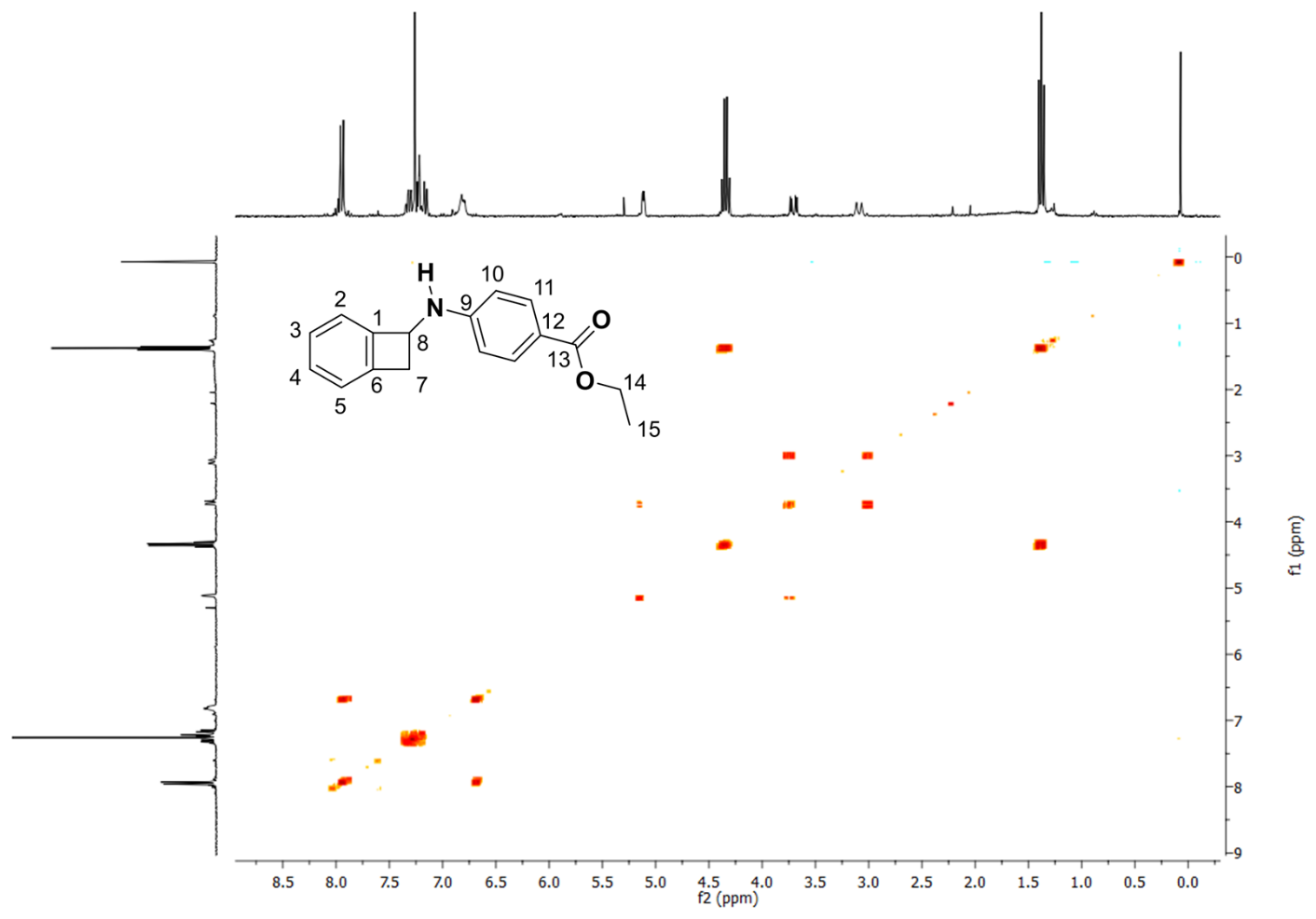


Figure S34. **COSY spectrum of BCB-NHPhCO_2_Et 4b**

Figure S35. **^13^C spectrum of BCB-NHPhCO_2_Et 4b**

Figure S36. **^13^C-APT spectrum of BCB-NHPhCO_2_Et 4b**


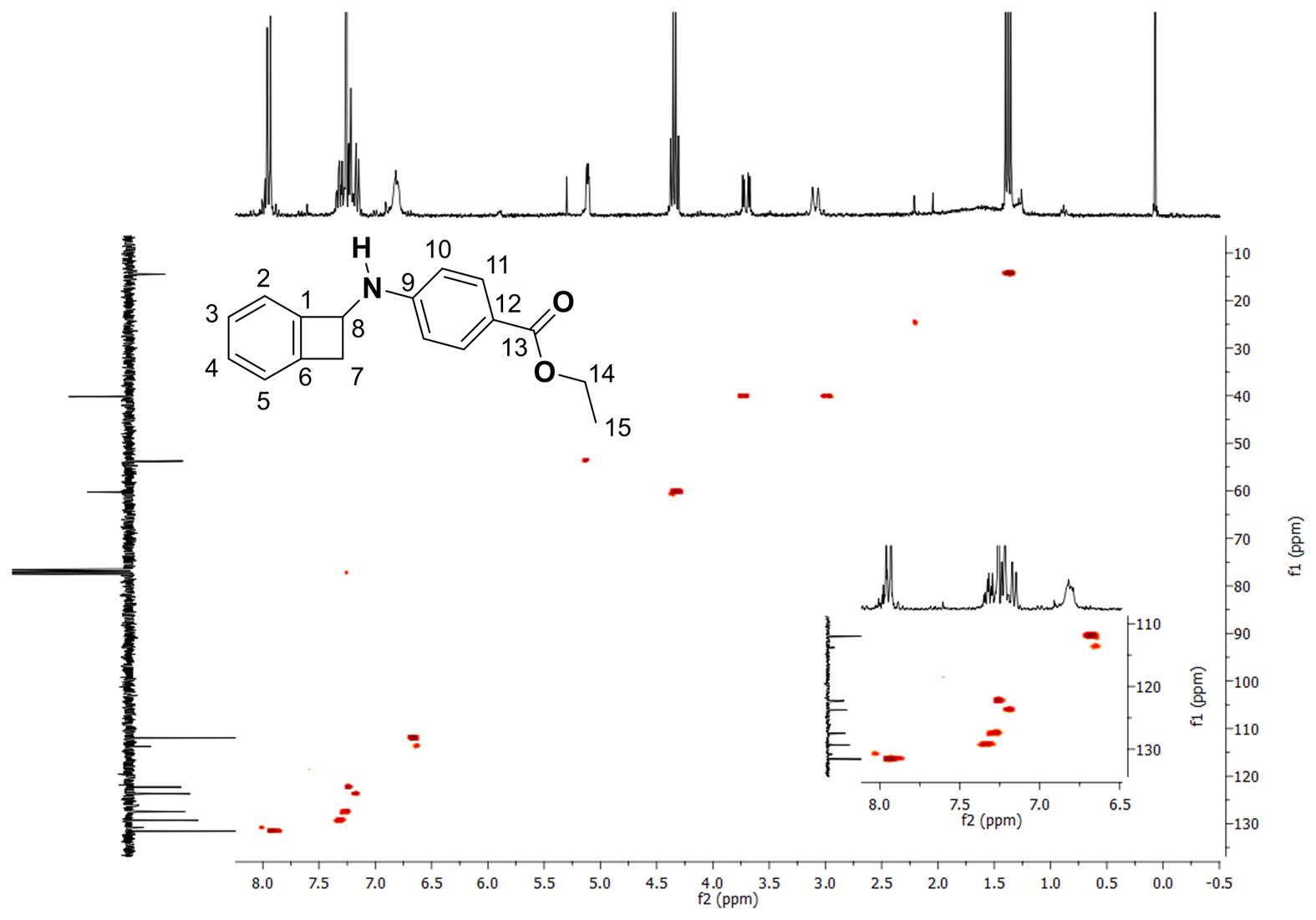


Figure S37. **HMQC spectrum of BCB-NHPhCO_2_Et 4b**

Figure S38. **^1^H NMR spectrum of BCB-NHPhOMe 4c**

Figure S39. **COSY NMR spectrum of BCB-NHPhOMe 4c**

Figure S40. **^13^C-APT NMR spectrum of BCB-NHPhOMe 4c**

Figure S41. **HMQC NMR spectrum of BCB-NHPhOMe 4c**

Figure S42. **^1^H NMR spectrum of BCB-NHPh 4d (* traces of EtOAc)**

Figure S43. **^13^C-APT-spectrum of BCB-NHPh 4d**

Figure S44. **^1^H NMR spectrum of BCB-N(COMe)PhCF_3_ 5a**


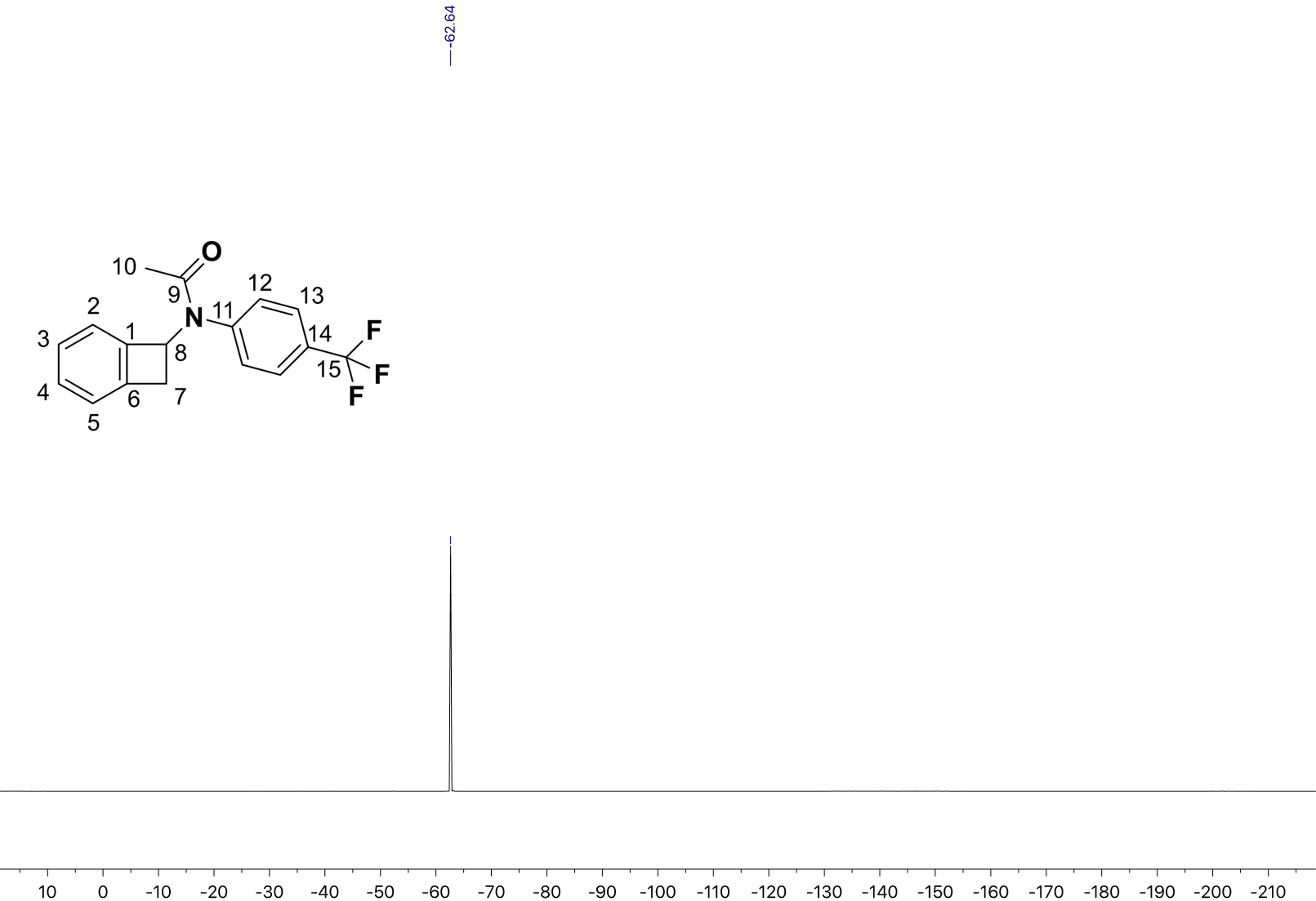


Figure S45. **^19^F NMR spectrum of BCB-N(COMe)PhCF_3_** **5a**


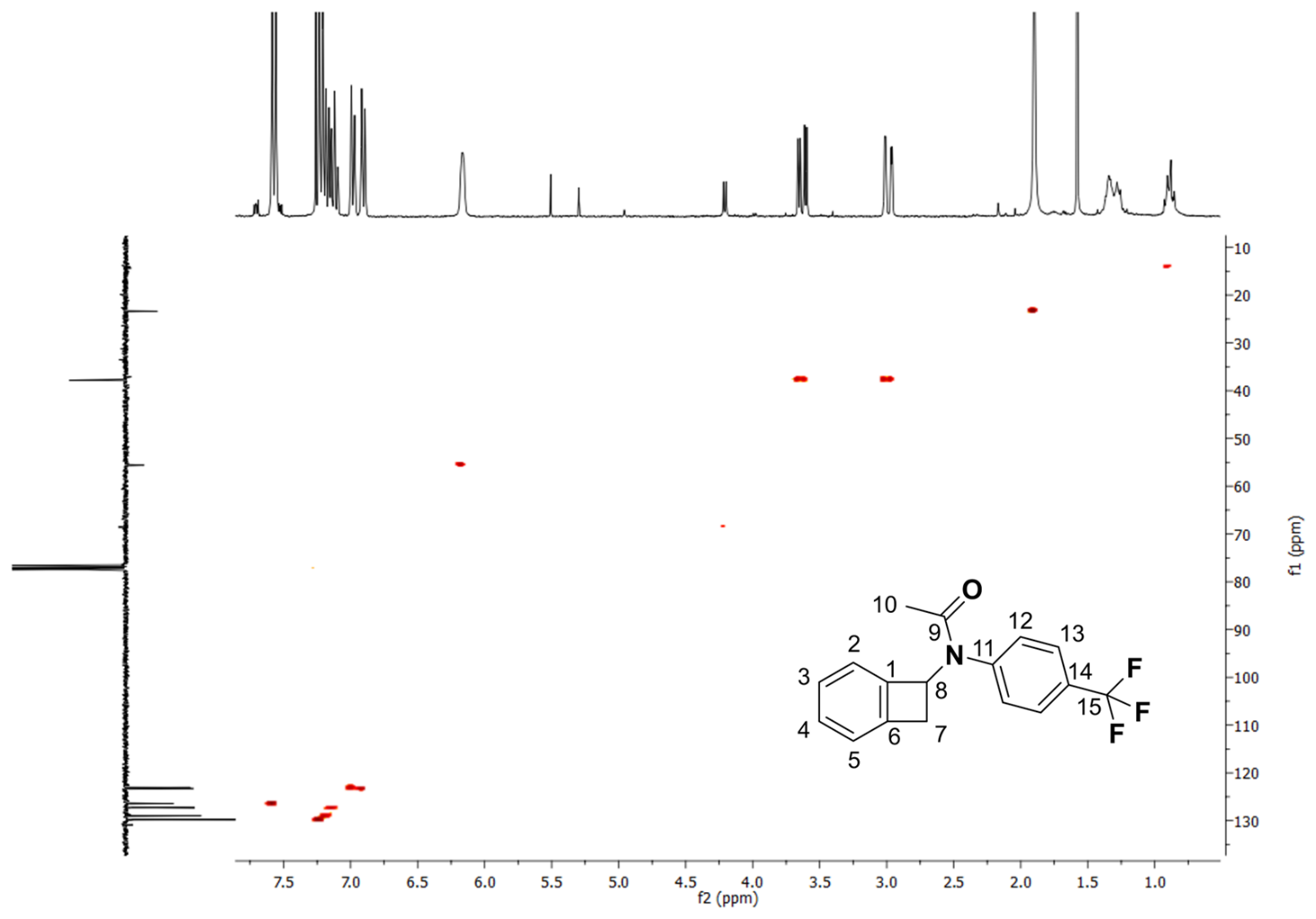


Figure S46. **HSQC NMR of BCB-N(COMe)PhCF_3_ 5a**

Figure S47. ***^13^C NMR (a) and ^13^C-APT NMR (b) spectra of BCB-N(COMe)PhCF_3_ 5a***


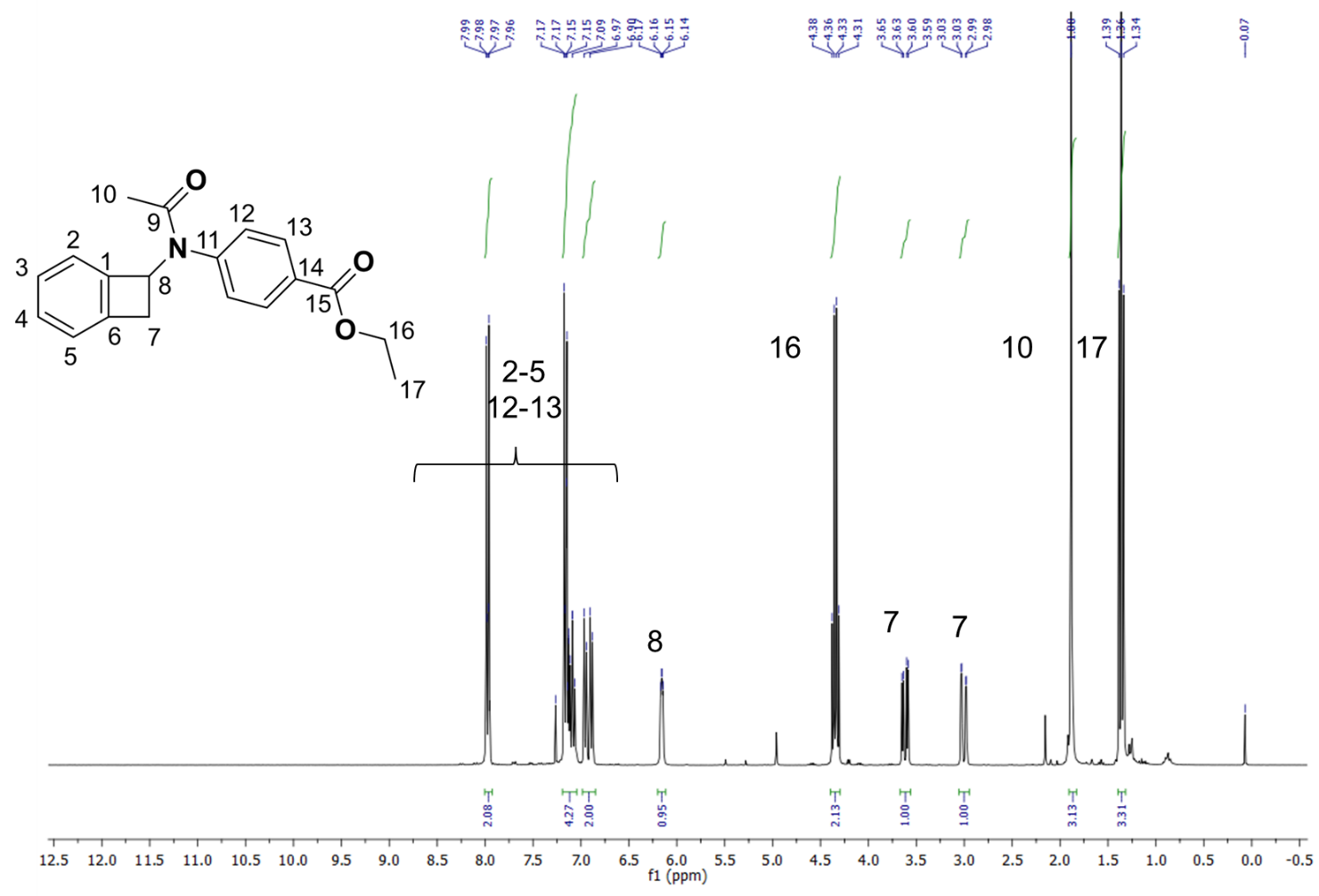


Figure S48. **^1^H NMR spectrum of BCB-N(COMe)PhCO_2_Et 5b**


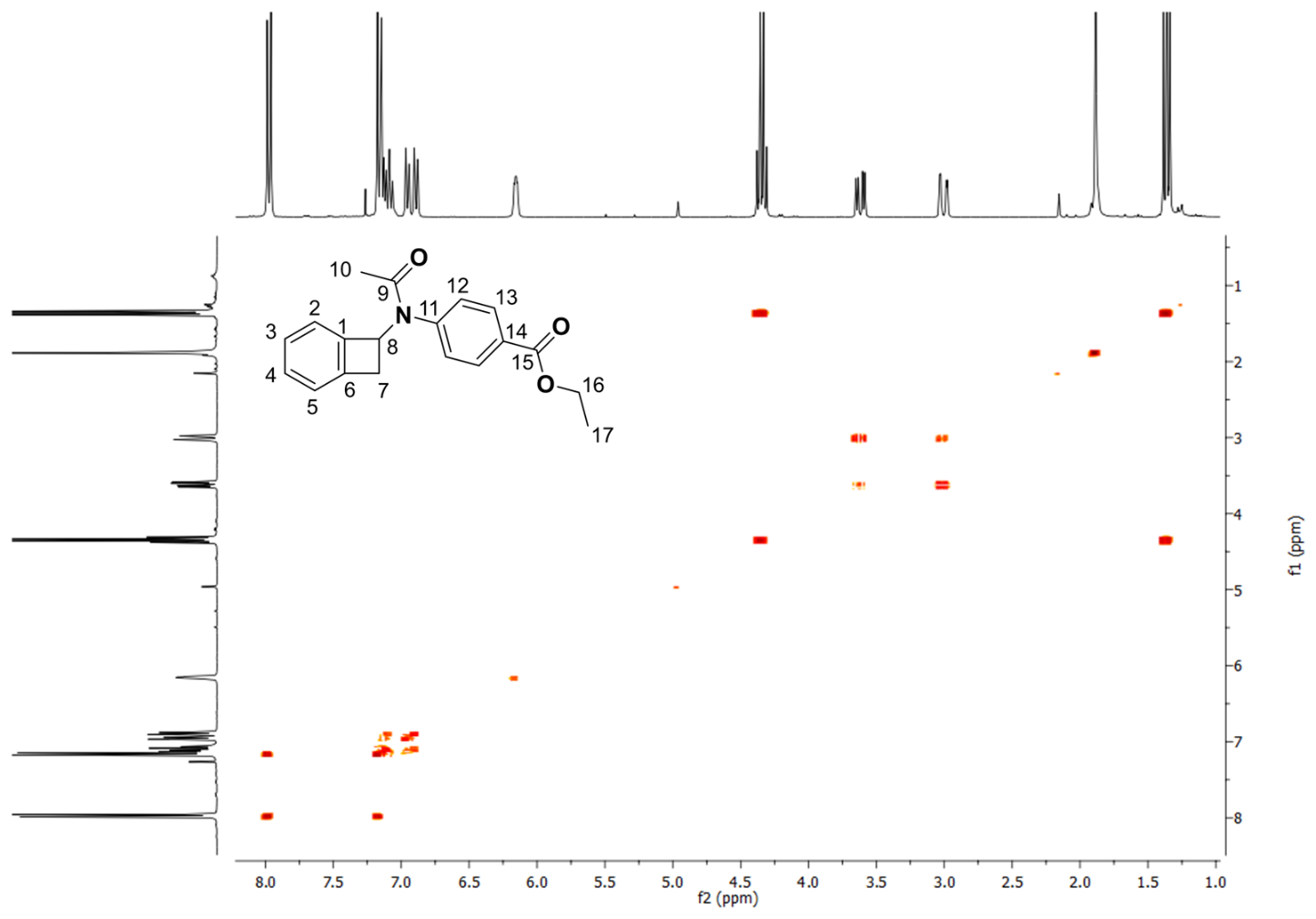


Figure S49. **COSY NMR of BCB-N(COMe)PhCO_2_Et 5b**

Figure S50. **^13^C-APT NMR spectrum of BCB-N(COMe)PhCO_2_Et 5b**

Figure S51. **HSQC NMR of BCB-N(COMe)PhCO_2_Et 5b**

Figure S52. **^1^H NMR spectrum of BCB-N(COMe)PhOMe 5c**

Figure S53. **COSY NMR spectrum of BCB-N(COMe)PhOMe 5c**

Figure S54. **^13^C-APT NMR spectrum of BCB-N(COMe)PhOMe 5c**

Figure S55. **(a) HSQC and (b) HSBC NMR spectra of BCB-N(COMe)PhOMe 5c**

Figure S56. **^1^H NMR spectrum of BCB-N(COMe)Ph 5d**


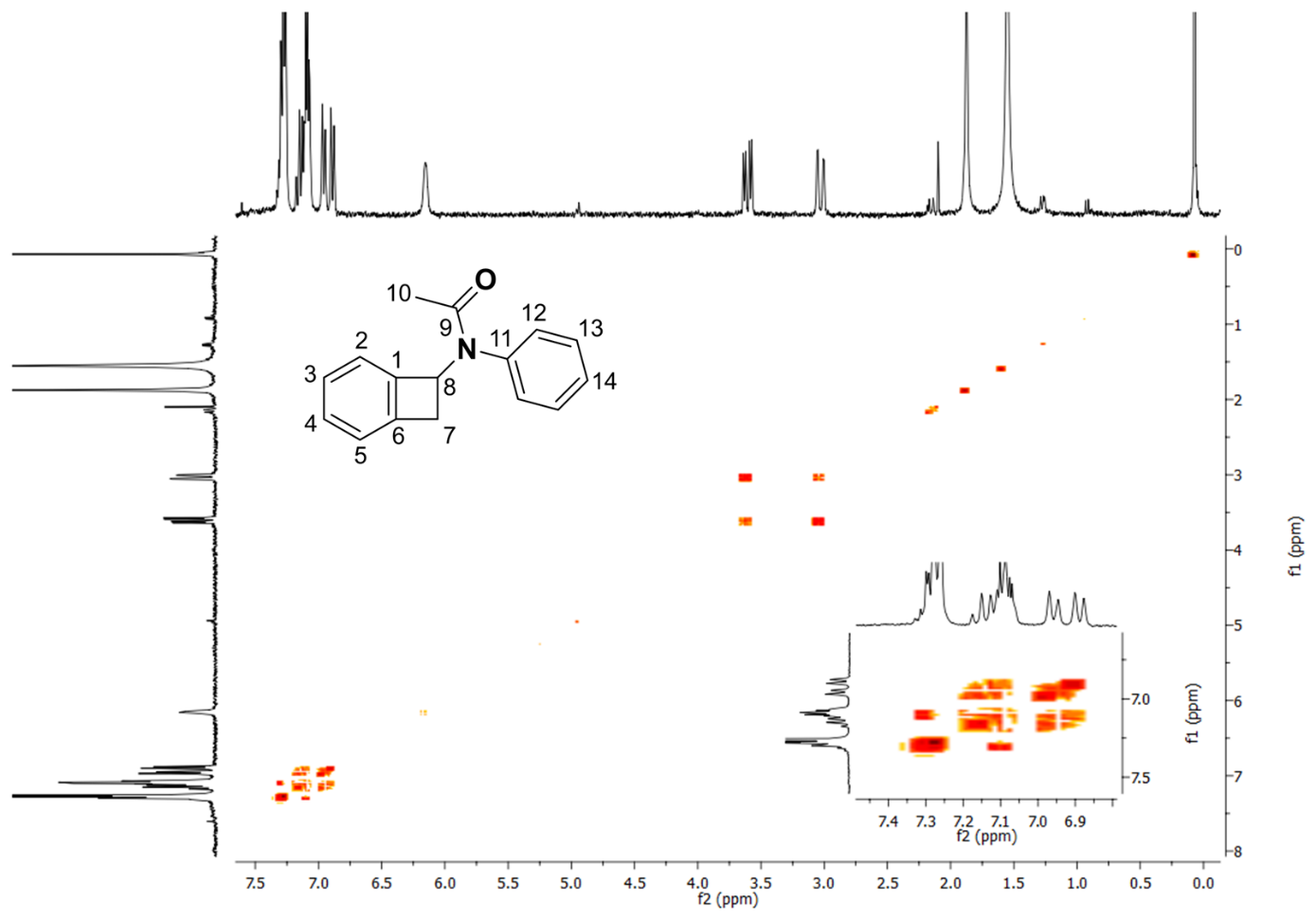


Figure S57. **COSY NMR spectrum of BCB-N(COMe)Ph 5d**


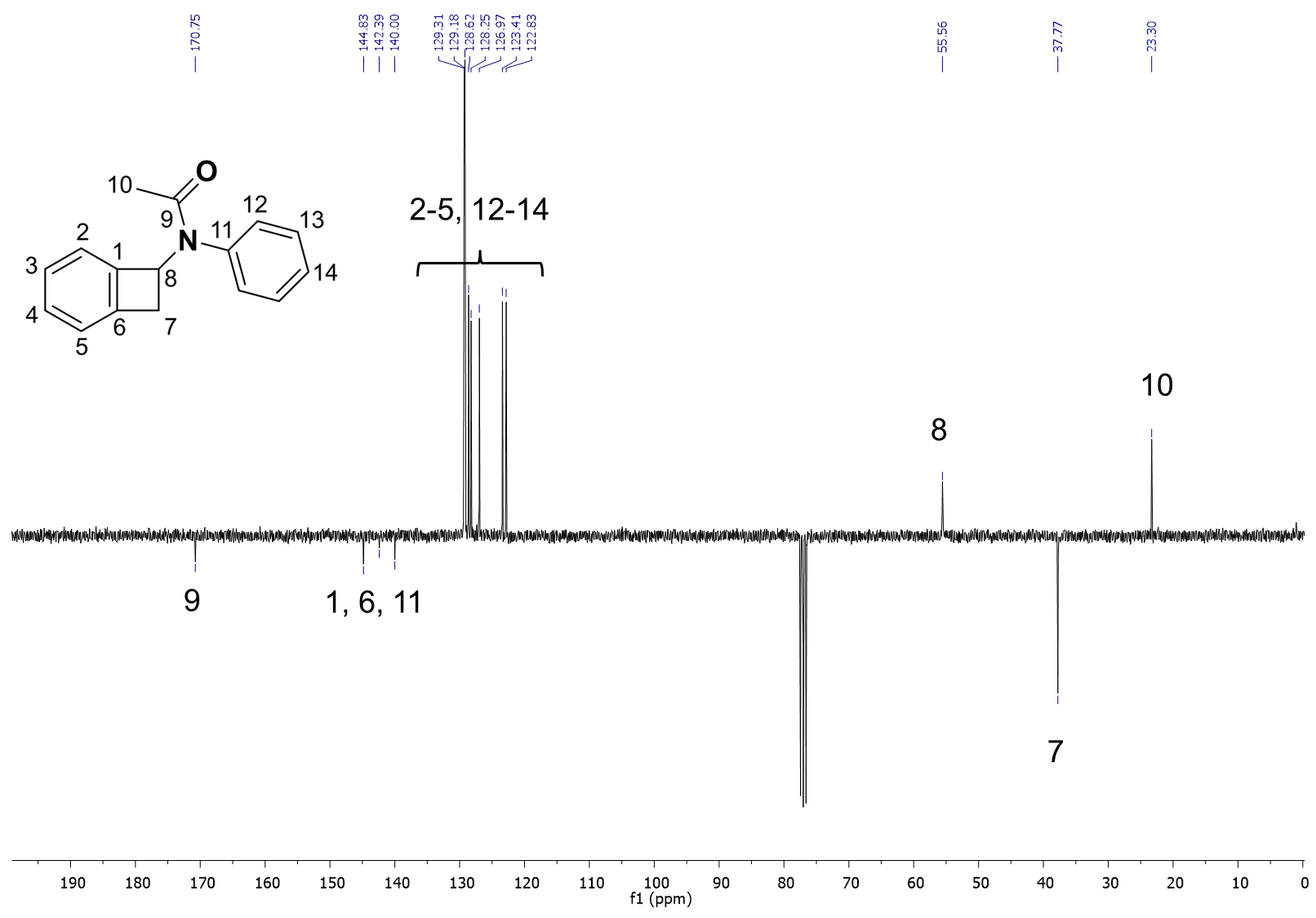


Figure S58. **^13^C-APT NMR spectrum of BCB-N(COMe)Ph 5d**


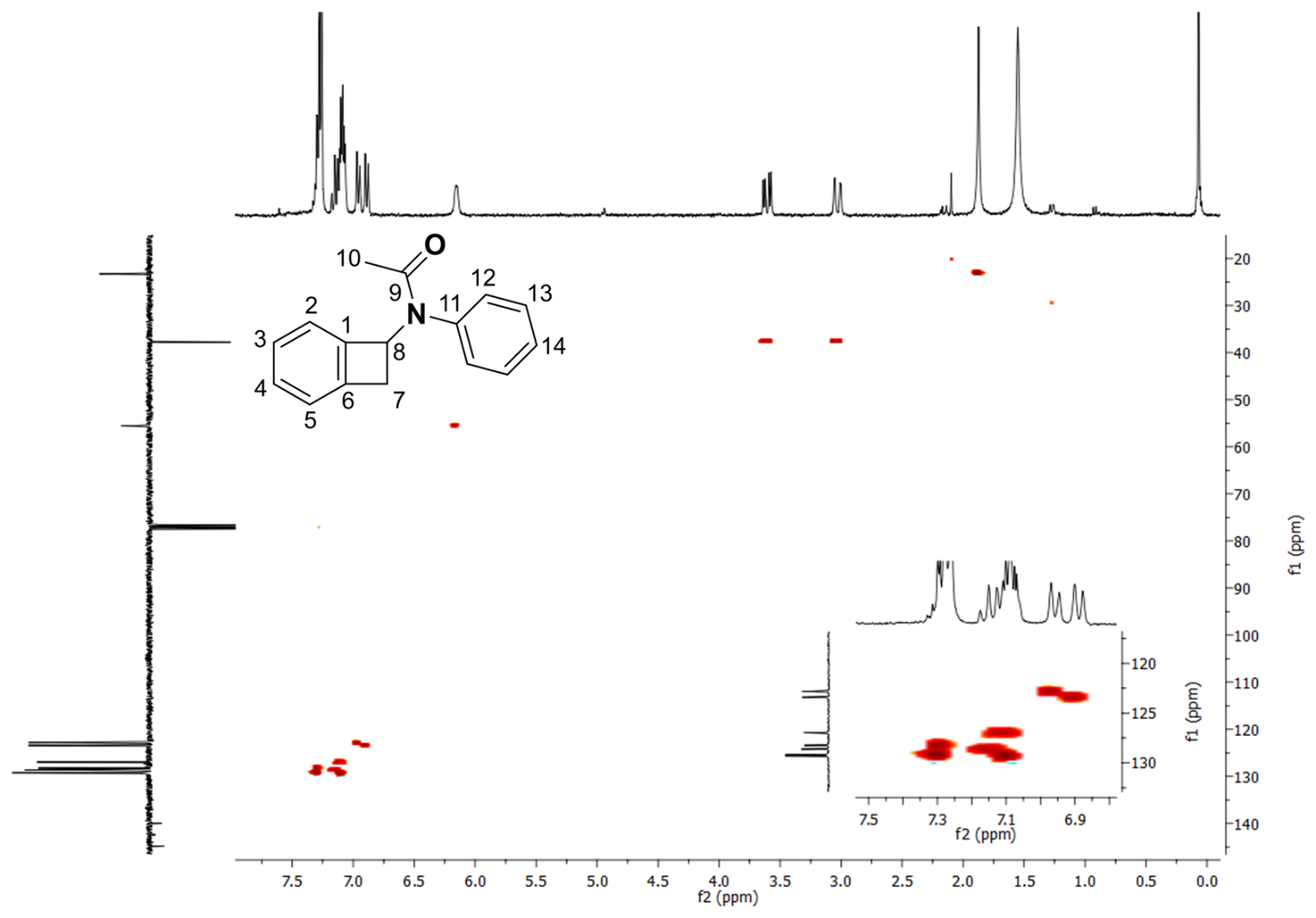


Figure S59. **HSQC NMR spectrum of BCB-N(COMe)Ph 5d**

Figure S60. **^1^H NMR spectrum of BCB-N(COOMe)PhCO_2_Et 6**


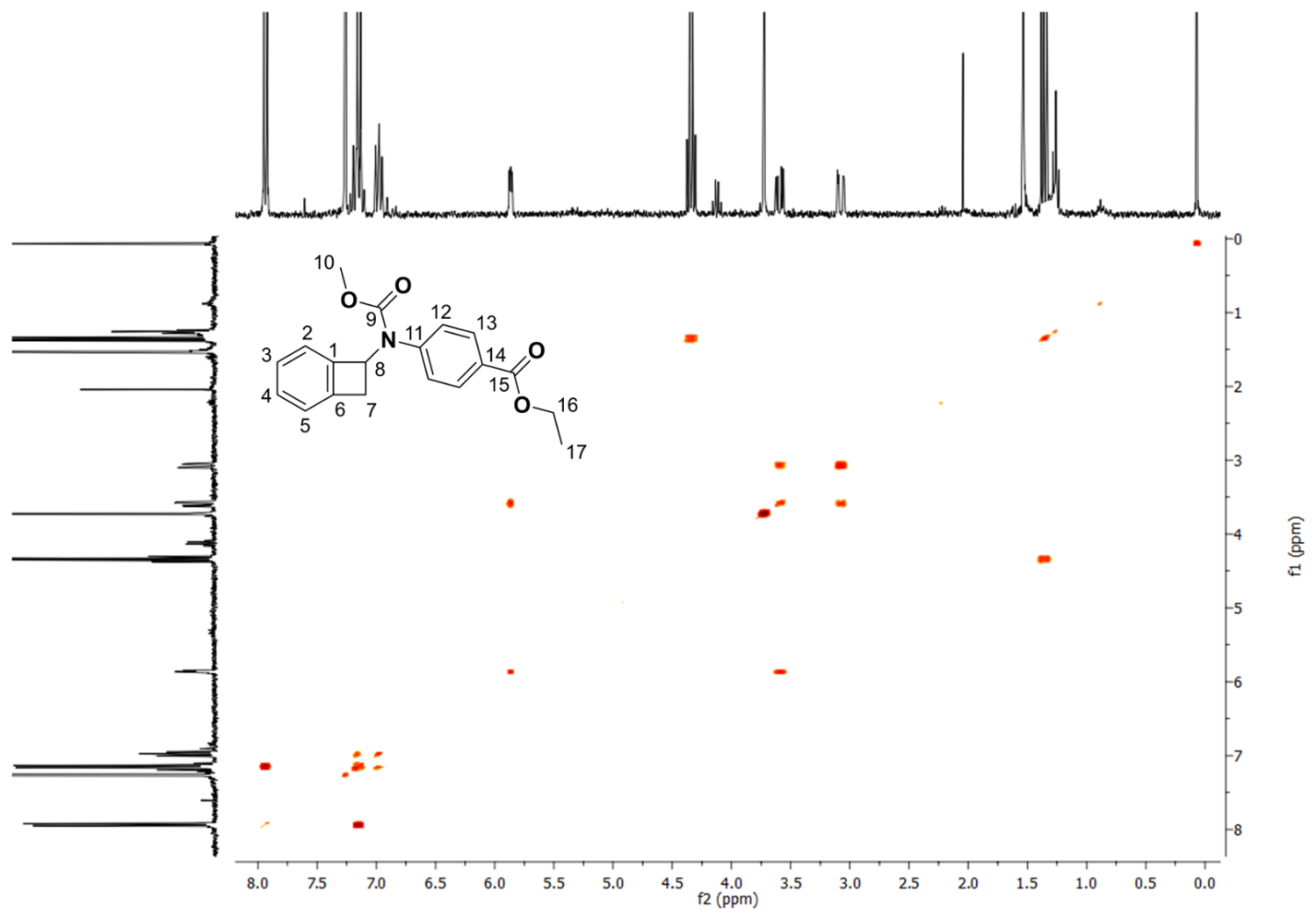


Figure S61. **COSY NMR spectrum of BCB-N(COOMe)PhCO_2_Et 6**

Figure S62. **^13^C-NMR (a) and  ^13^C-APT NMR (b) spectra of BCB-N(COOMe)PhCO_2_Et 6**

Figure S63. **HMQC NMR spectrum of BCB-N(COOMe)PhCO_2_Et 6**

**^
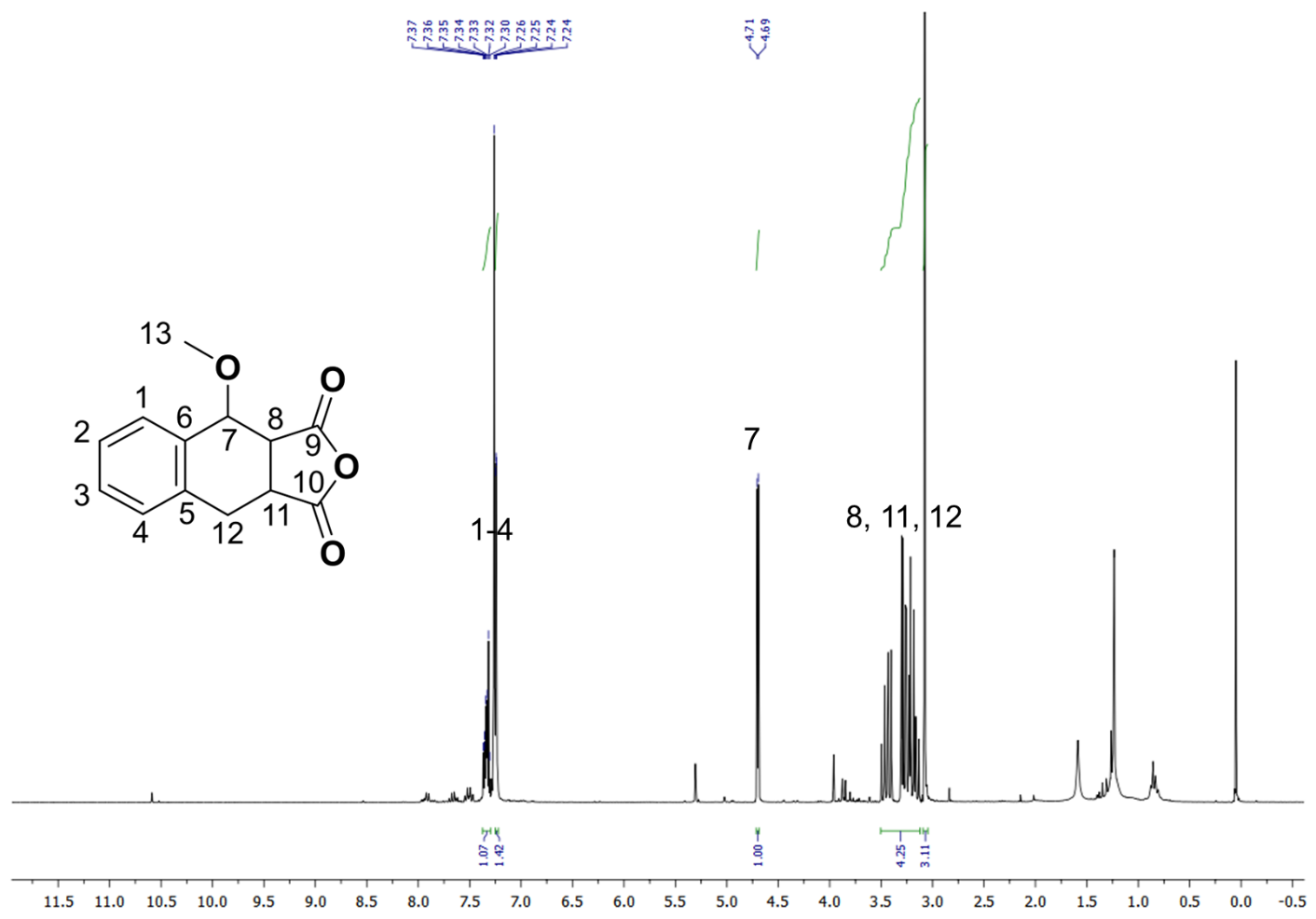
^**

Figure S64. **^1^H NMR spectrum of 7a, Diels-Alder adduct of BCB-OMe**


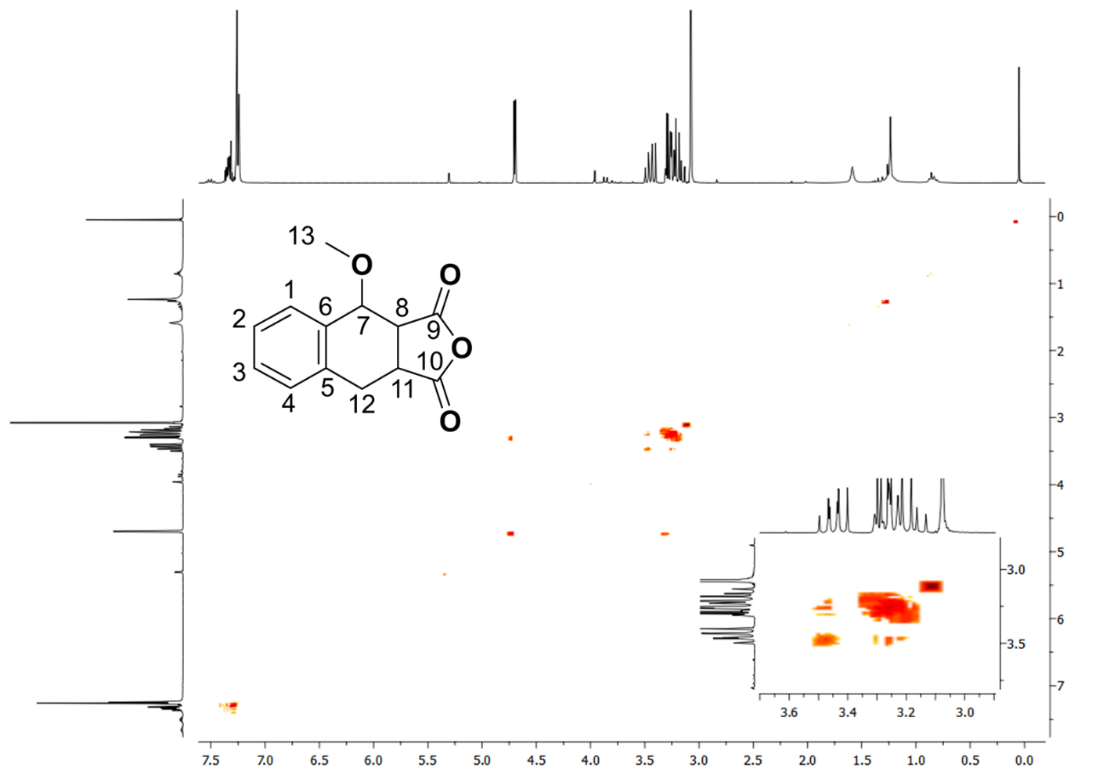


Figure S65. **COSY NMR spectrum of 7a, Diels-Alder adduct of BCB-OMe**

Figure S66. **^13^C-APT NMR spectrum of 7a, Diels-Alder adduct of BCB-OMe**


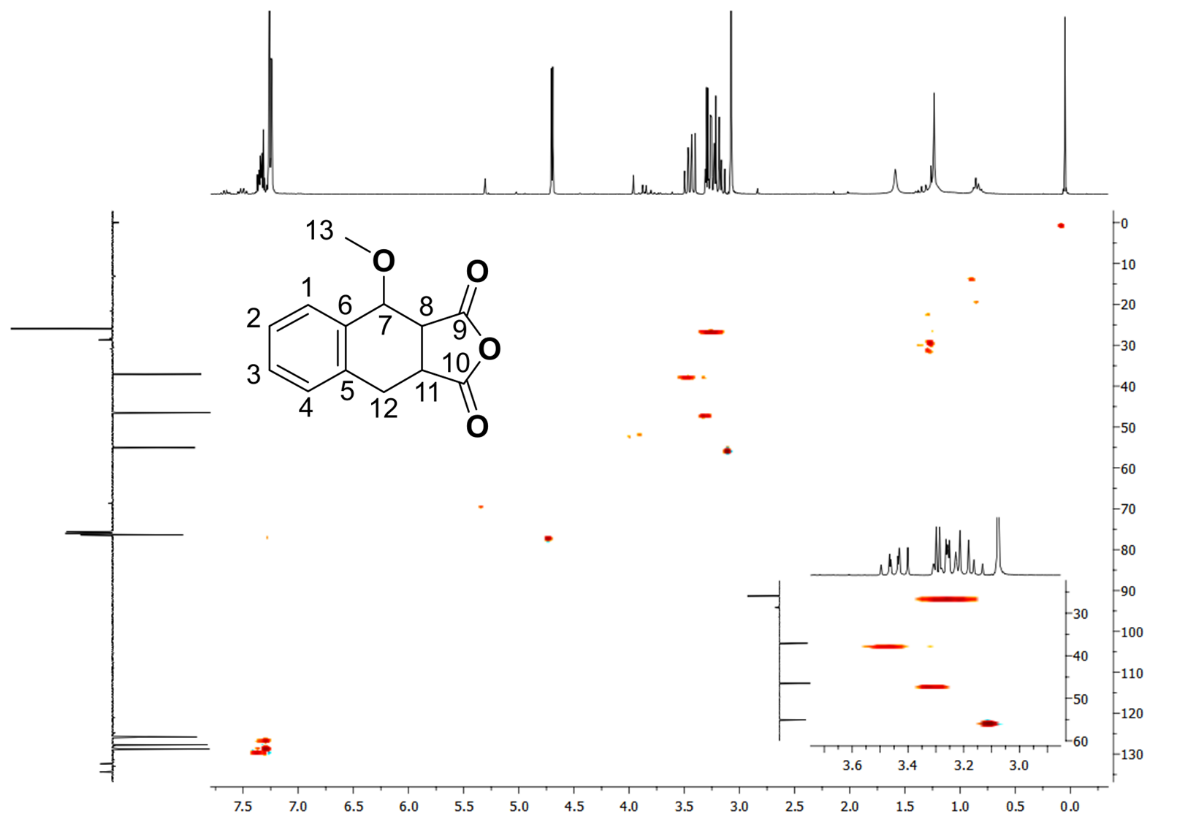


Figure S67. **HSQC NMR spectrum of 7a, Diels-Alder adduct of BCB-OMe**

###
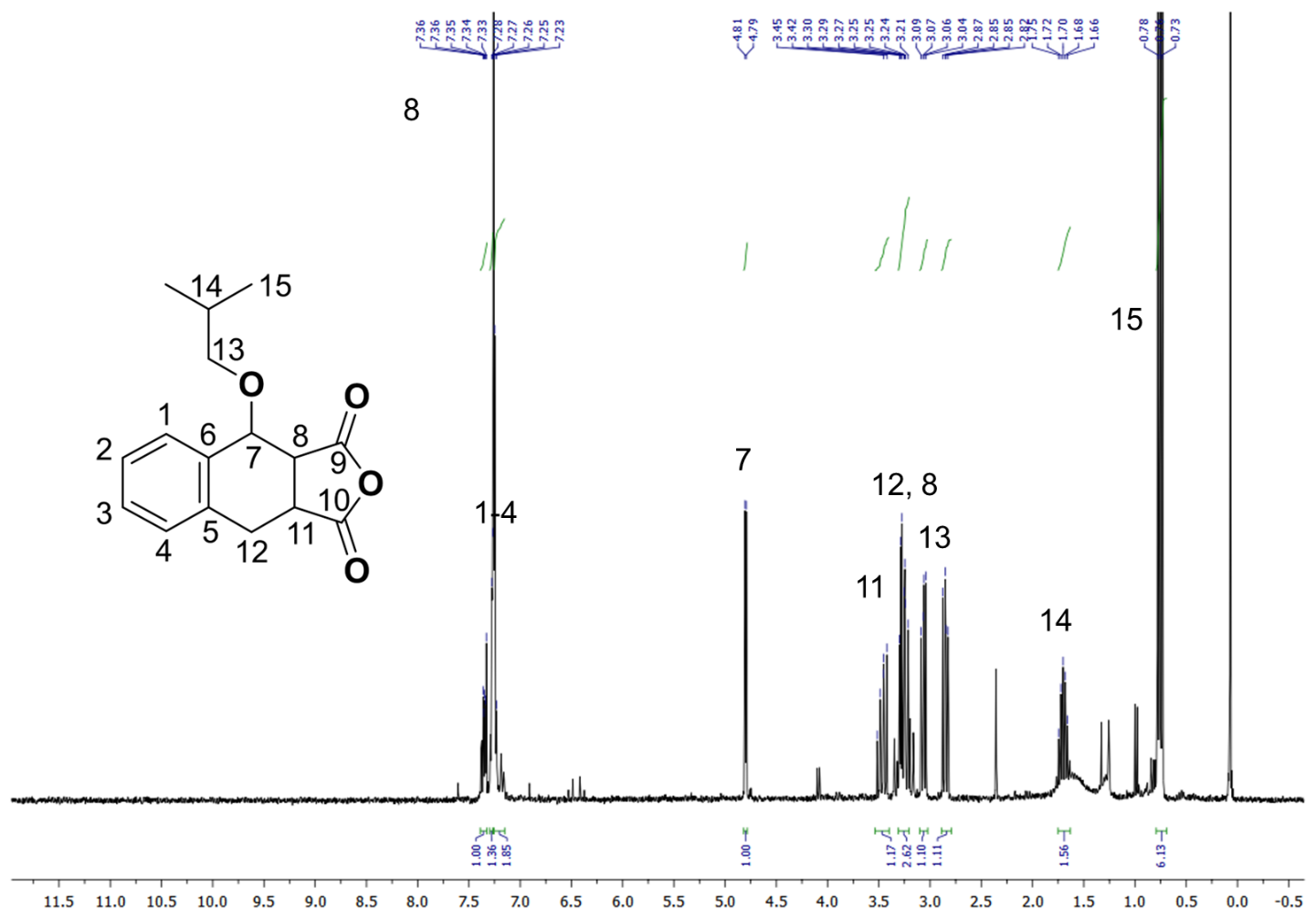


Figure S68. **^1^H NMR spectrum of 7b, Diels-Alder adduct of BCB-OiBu**

###
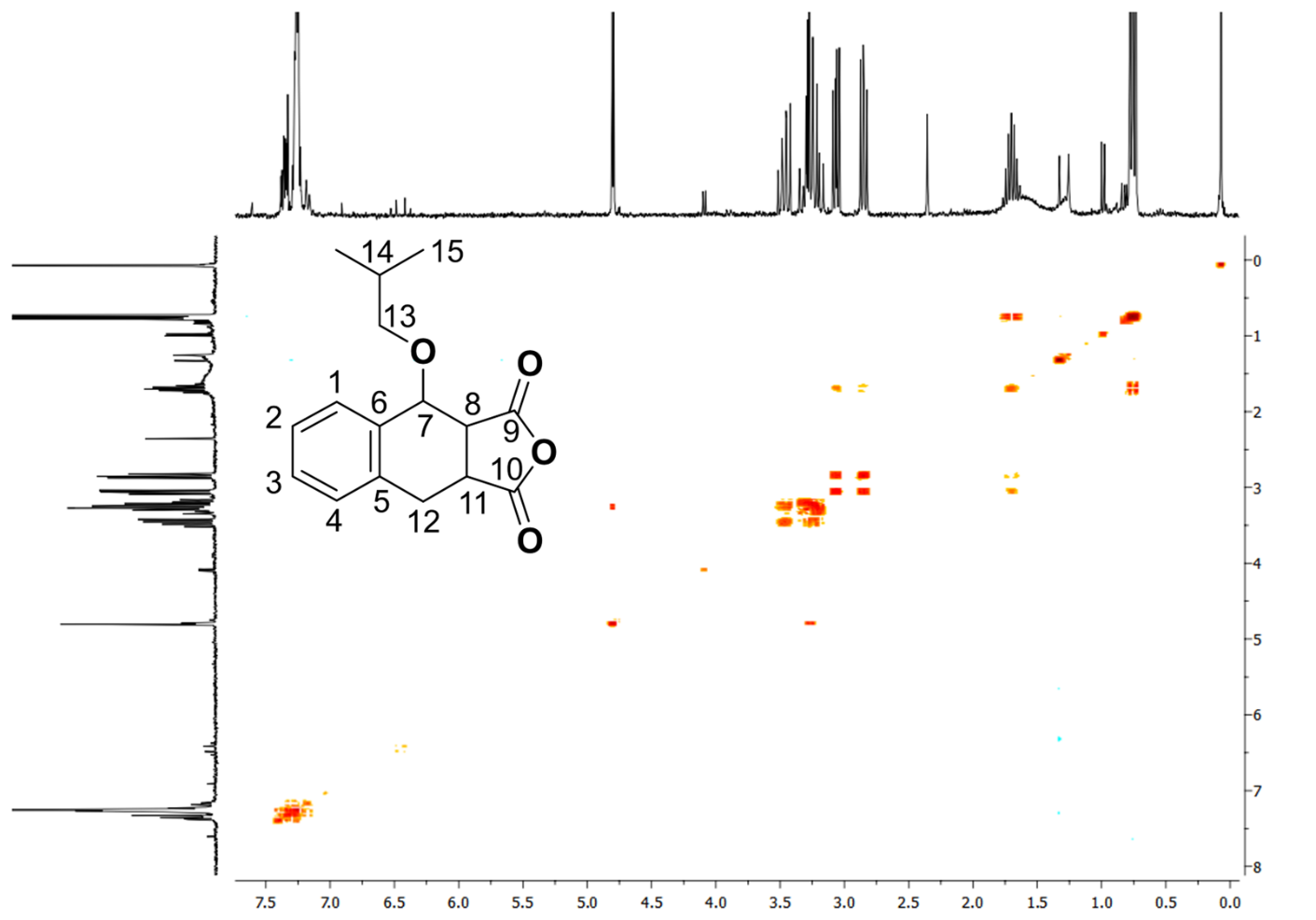


Figure S69. **COSY NMR spectrum of 7b, Diels-Alder adduct of BCB-OiBu**

Figure S70. **^13^C NMR spectrum of 7b, Diels-Alder adduct of BCB-OiBu**


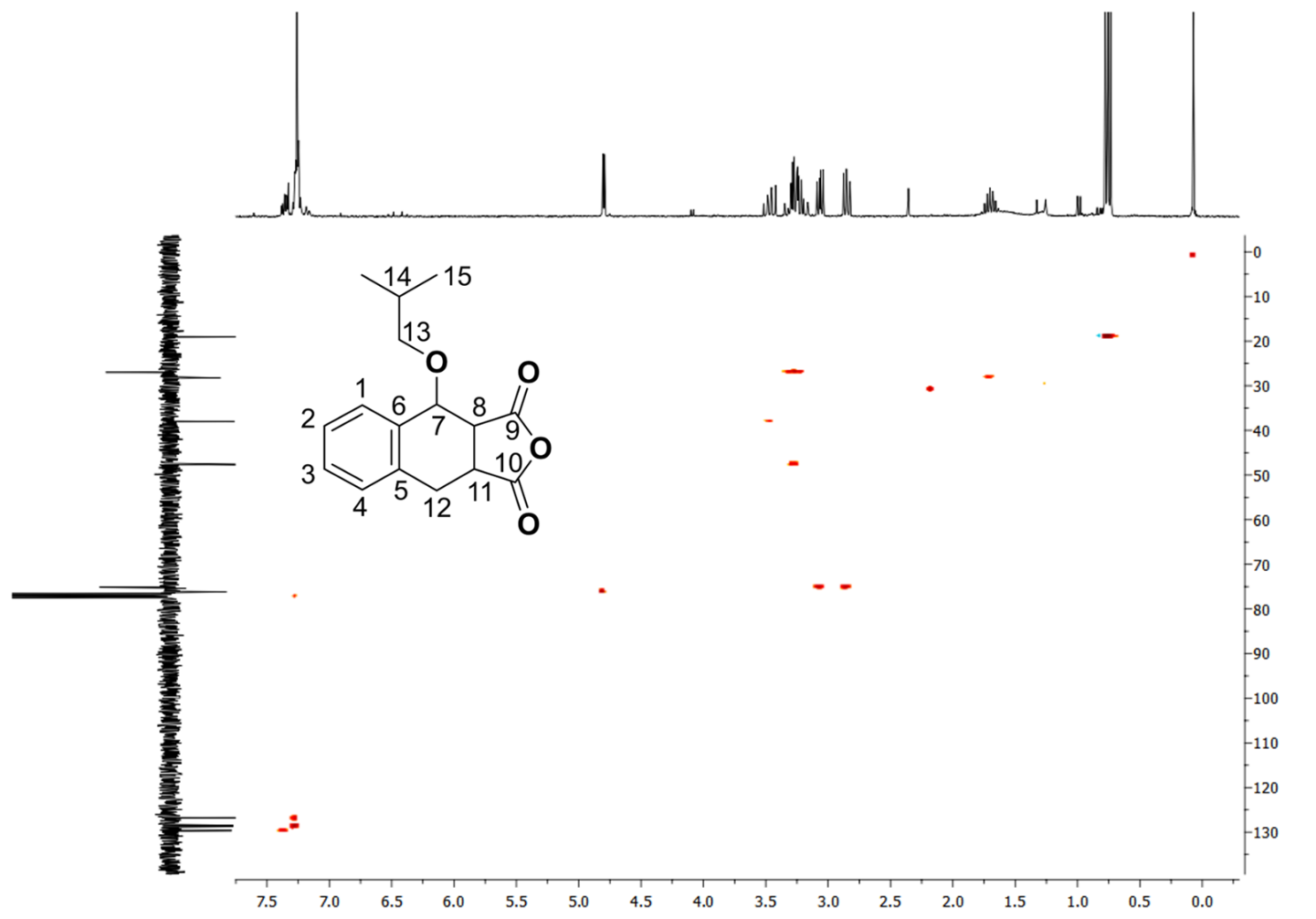


Figure S71. **HSQC NMR spectrum of 7b, Diels-Alder adduct of BCB-OiBu**

Figure S72. **(a)** **^1^H NMR spectrum, (b) COSY and DSC of 7c, Diels-Alder adduct of BCB-OTMS**

Figure S73. **(a) ^13^C-APT NMR spectrum, (b) HSQC and (c) HSBC of 7c,**

**Diels-Alder adduct of BCB-OTMS**

Figure S74. **^1^H NMR and ^19^F NMR spectra of 7d, Diels-Alder adduct of BCB-NHPhCF_3_**

Figure S75. **COSY NMR spectrum of 7d, Diels-Alder adduct of BCB-NHPhCF_3_**

Figure S76. **^13^C-NMR, ^13^C-APT NMR spectra of 7d, Diels-Alder adduct of BCB-NHPhCF_3_**

Figure S77. **HMQC NMR spectrum of 7d, Diels-Alder adduct of BCB-NHPhCF_3_**

Figure S78. **NOE NMR spectrum of 7d, Diels-Alder adduct of BCB-NHPhCF_3_**

Figure S79. **^1^H NMR (a) and COSY (b) spectra of 7e, Diels-Alder adduct of BCB-NHPhCO_2_Et**

Figure S80. **^13^C-APT NMR spectrum of 7e, Diels-Alder adduct of BCB-NHPhCO_2_Et**

Figure S81. **HMQC NMR spectrum of 7e, Diels-Alder adduct of BCB-NHPhCO_2_Et**

Figure S82. **HMBC NMR spectrum of 7e, Diels-Alder adduct of BCB-NHPhCO_2_Et**

Figure S83. **NOESY NMR spectrum of 7e, Diels-Alder adduct of BCB-NHPhCO_2_Et**

Figure S84. **^1^H NMR spectrum of 7f, Diels-Alder adduct of BCB-N(COOMe)PhCO_2_Et**

Figure S85. **COSY NMR spectrum of 7f, Diels-Alder adduct of BCB-N(COOMe)PhCO_2_Et**

Figure S86. **^13^C NMR spectrum of 7f, Diels-Alder adduct of BCB-N(COOMe)PhCO_2_Et**


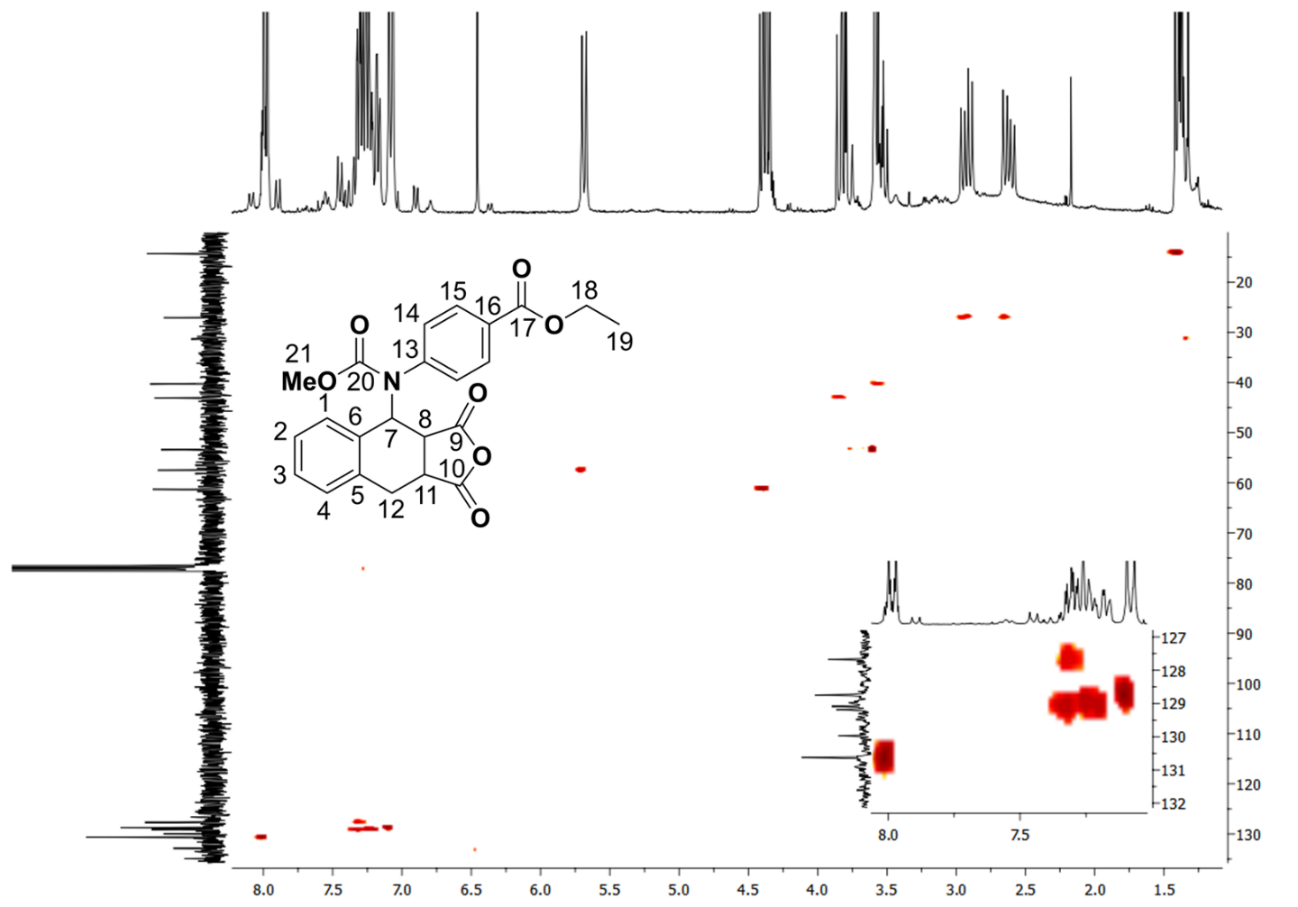


Figure S87. **HMBC NMR spectrum of 7f, Diels-Alder adduct of BCB-N(COOMe)PhCO_2_Et**

Figure S89. **^1^H NMR spectra of BCB-OMe 2a over time in the presence of TEMPO (conditions: 110 °C, *t*Bbenz, 3 equiv TEMPO, aliquot analysis).**


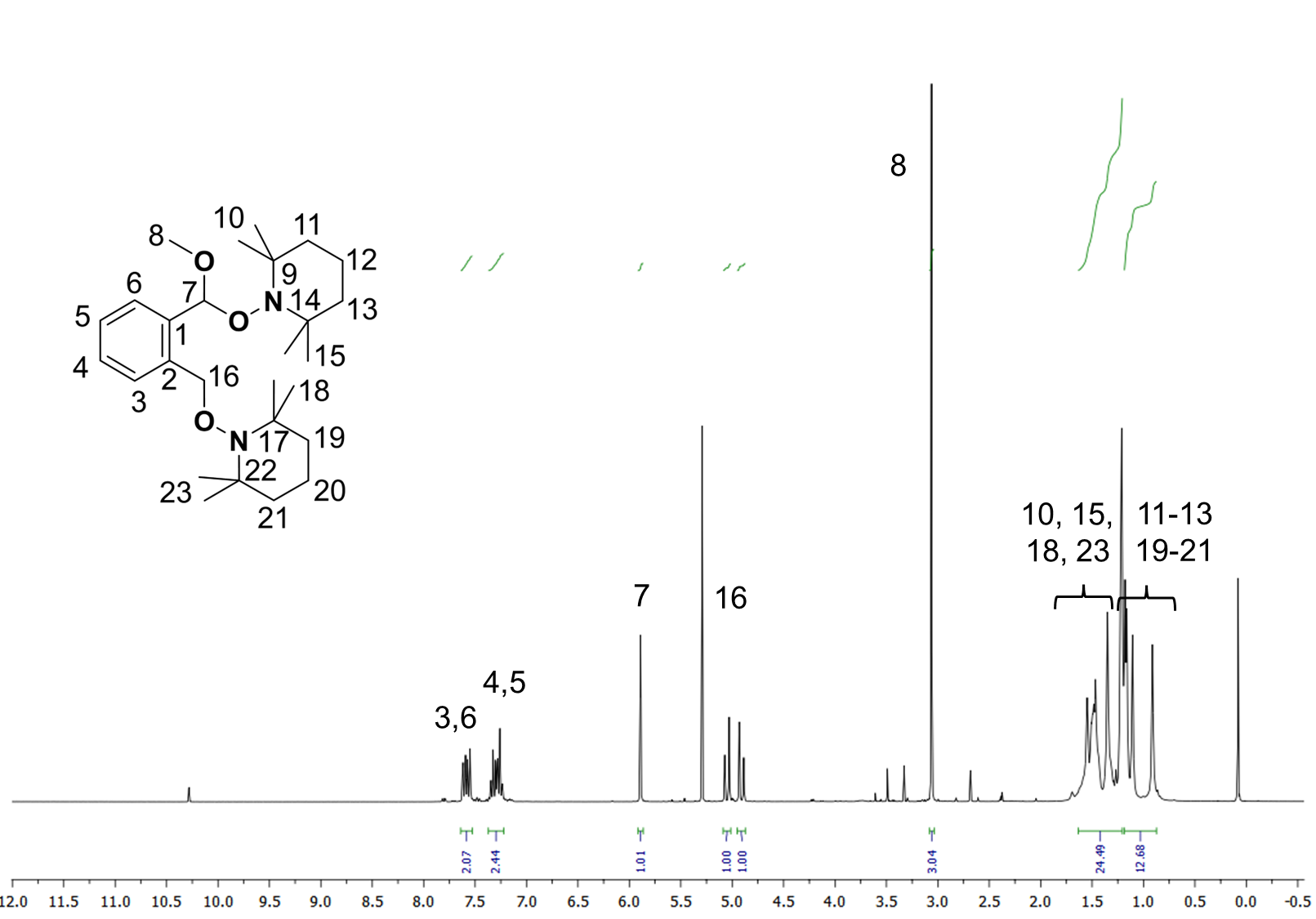


Figure S88. **^1^H NMR spectrum of 8a, TEMPO adduct of BCB-OMe**

Figure S90. **COSY NMR spectrum of 8a, TEMPO adduct of BCB-OMe**

Figure S91. **^13^C NMR spectrum of 8a, TEMPO adduct of BCB-OMe**

Figure S92. **HSQC NMR spectrum of 8a, TEMPO adduct of BCB-OMe**

Figure S93. **^1^H NMR (a) and COSY (b) spectra of 8b, TEMPO adduct of BCB-OiBu 2b**


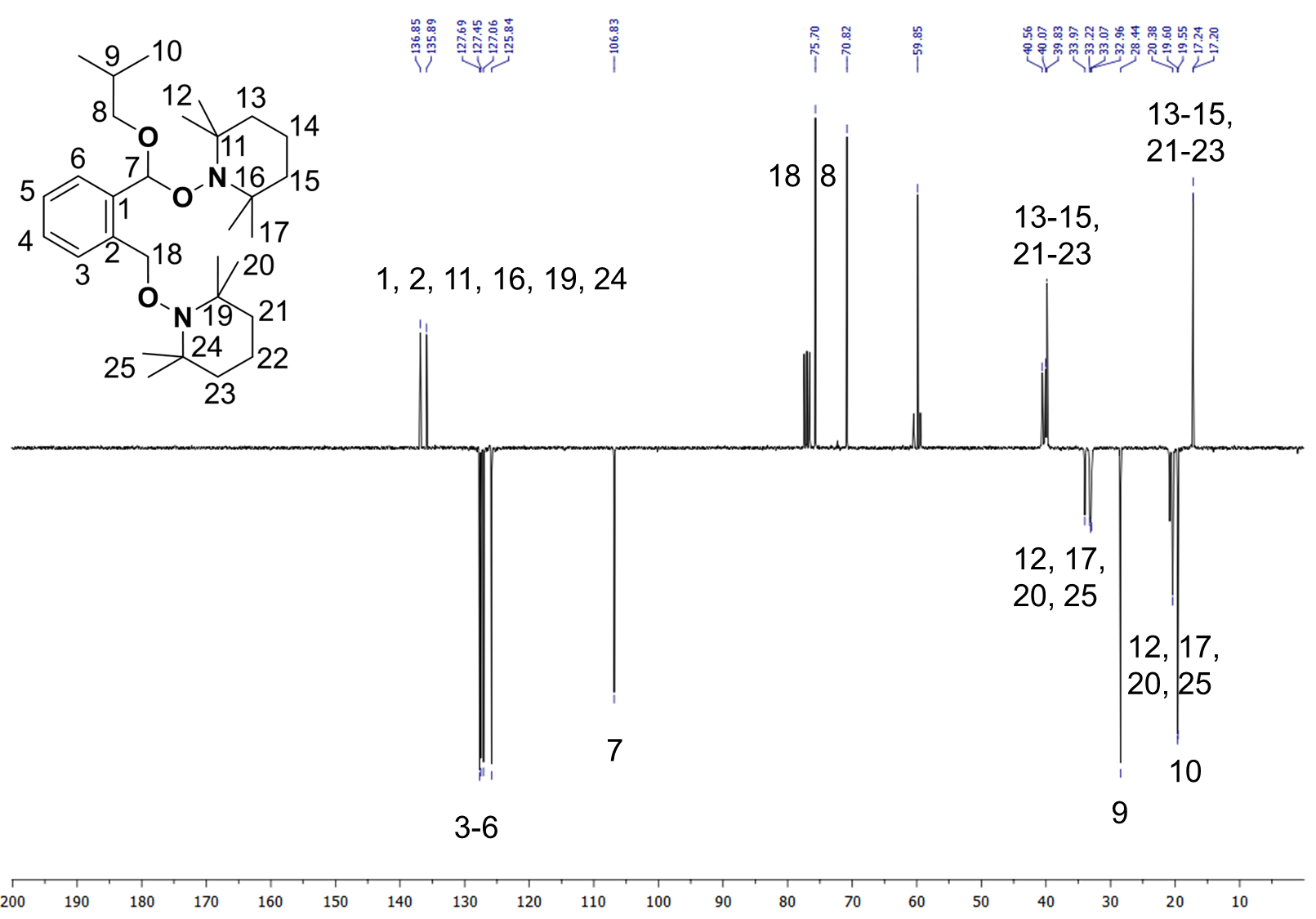


Figure S94. **^13^C-APT NMR spectrum of 8b, TEMPO adduct of BCB-OiBu**

Figure S95. **HMQC NMR spectrum of 8b, TEMPO adduct of BCB-OiBu**

Figure S96. **^1^H NMR spectrum of 8c, TEMPO adduct of BCB-N(COMe)PhCO_2_Et**

Figure S97. **COSY NMR spectrum of 8c, TEMPO adduct of BCB-N(COMe)PhCO_2_Et**

Figure S98. **^13^C-APT NMR spectrum of 8c, TEMPO adduct of BCB-N(COMe)PhCO_2_Et**

Figure S99. **HMQC NMR spectrum of 8c, TEMPO adduct of BCB-N(COMe)PhCO_2_Et**

Figure 100. ^1^H NMR spectra of BCB **6** with time in presence of TEMPO (conditions: 105 °C, solvent tBbenz, 3 equiv. of TEMPO, analysis of aliquots).

Figure S101. **^1^H NMR spectrum of 8d, TEMPO adduct of BCB-N(CO_2_Me)PhCO_2_Et**


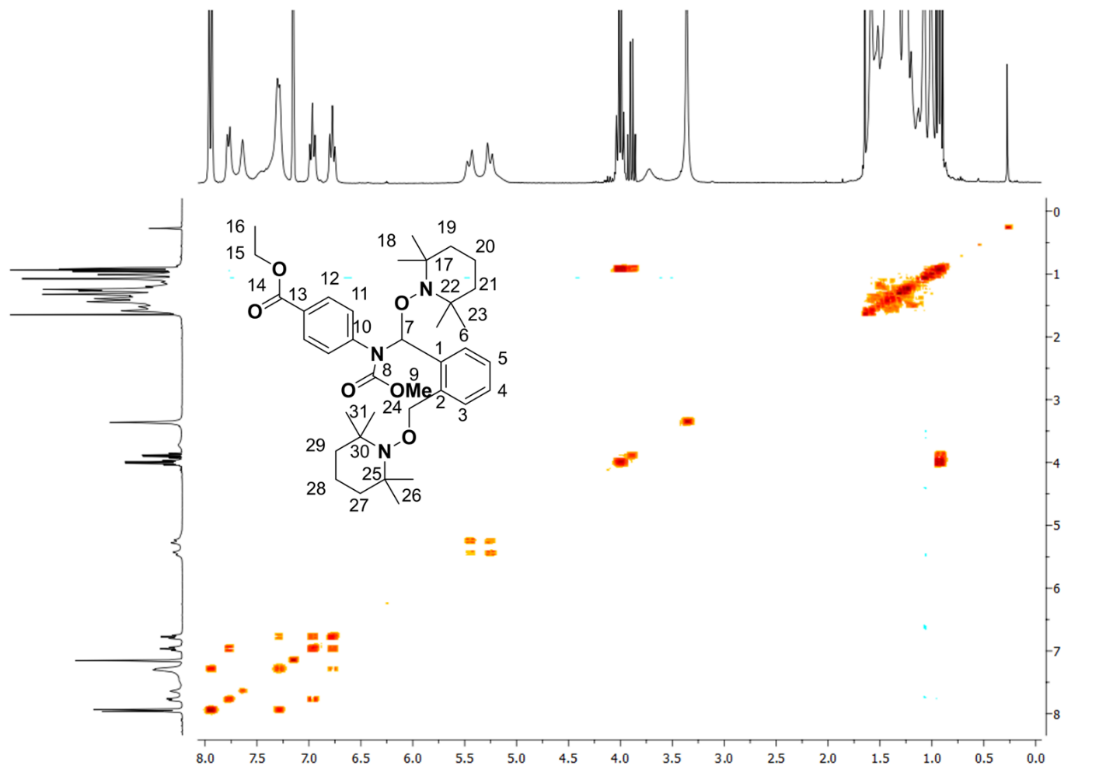


Figure S102. **COSY NMR spectrum of 8d, TEMPO adduct of BCB-N(CO_2_Me)PhCO2Et**


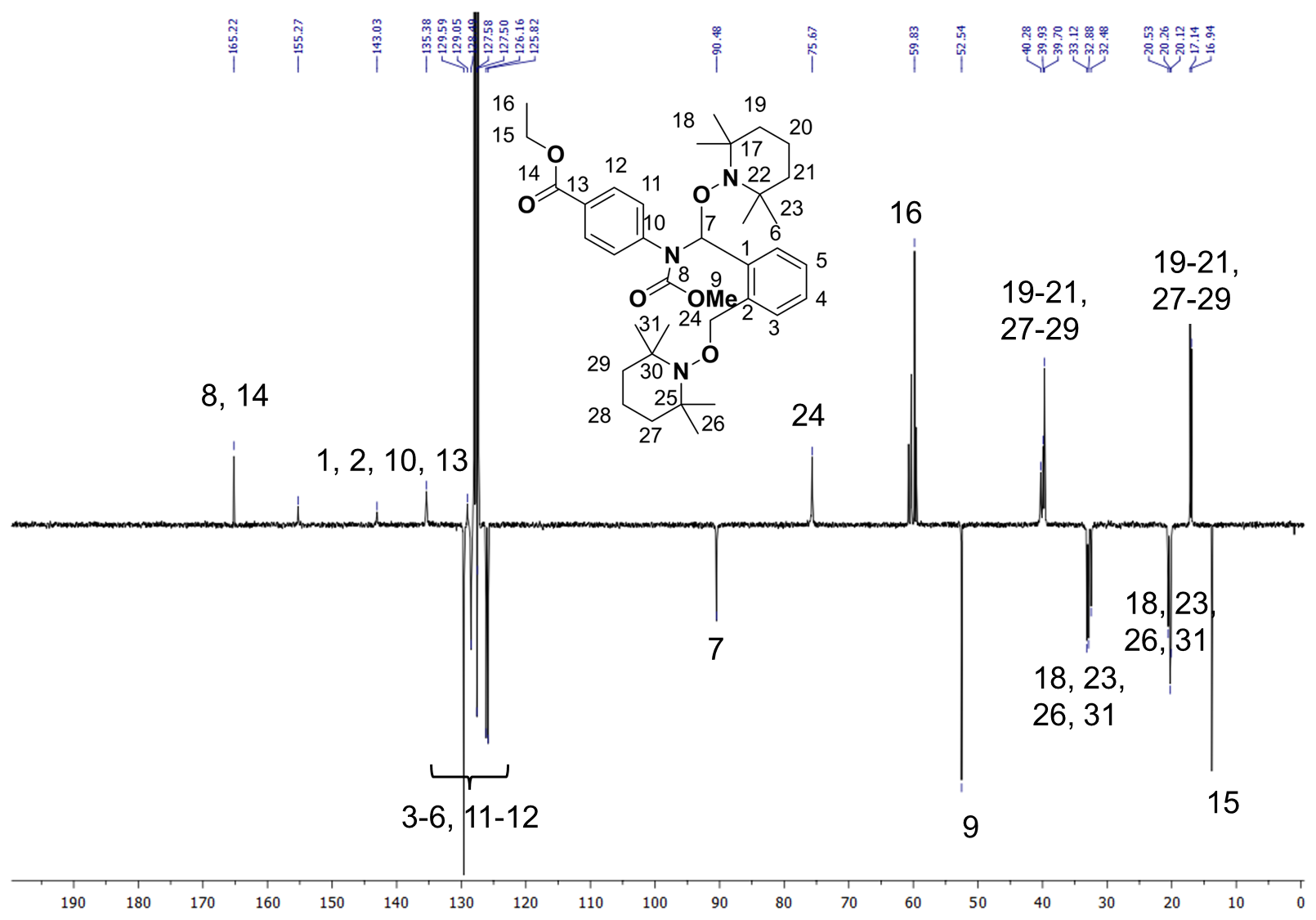


Figure S103. **^13^C-APT NMR spectrum of 8d, TEMPO adduct of BCB-N(CO_2_Me)PhCO2Et**


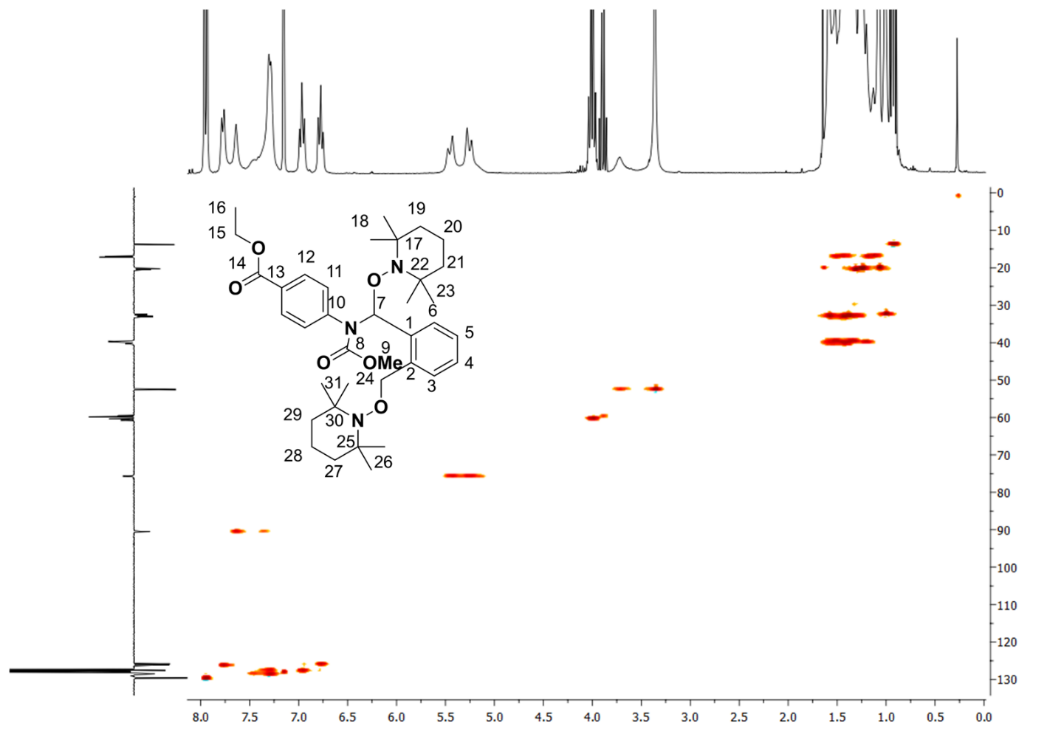


Figure S104. **HMQC NMR spectrum of 8d, TEMPO adduct of BCB-N(CO_2_Me)PhCO_2_Et**

Figure S105. **^1^H NMR spectrum of benzaldehyde-TEMPO alkoxyamine 9**

Figure S106. **^13^C-APT NMR spectrum of benzaldehyde-TEMPO alkoxyamine 9**

Figure S107. **COSY NMR spectrum of benzaldehyde-TEMPO alkoxyamine 9**

Figure S108. **HMQC NMR spectrum of benzaldehyde-TEMPO alkoxyamine 9**

Figure S109. **^1^H NMR and ^19^F NMR spectra of 10a, O_2_ adduct of BCB-NHPhCF_3_**

Figure S110. **Oxidation of BCB 4a to the O_2_ adduct 10a**(conditions: 70 mg 3 mL of dry CH_2_Cl_2_, O_2_ atmosphere, CH_2_Cl_2_, conversion determined by ^1^H NMR)

Figure S111. **COSY NMR spectrum of 10a, O_2_ adduct of BCB-NHPhCF_3_**

Figure S112. ^13^**C NMR spectrum of 10a, O_2_ adduct of BCB-NHPhCF_3_**

Figure S113. **HMQC NMR (a) and HMBC NMR (b) spectra of 10a, O_2_ adduct of BCB-NHPhCF_3_**

### Figure S114. **^1^H NMR spectrum of 10b, O_2_ adduct of BCB-NHPhCO_2_Et**

Figure S115. **Oxidation of BCB 4b to the O_2_ adduct 10b**(conditions: 70 mg 3 mL of dry CH_2_Cl_2_, O_2_ atmosphere, CH_2_Cl_2_, conversion determined by ^1^H NMR)

Figure S116. **COSY NMR spectrum of 10b, O_2_ adduct of BCB-NHPhCO_2_Et**

###

### Figure S117. **^13^C-NMR (a) and ^13^C-APT NMR (b) spectra of 10b, O_2_ adduct of BCB-NHPhCO_2_Et**

Figure S118. **HMQC NMR (a) and HMBC NMR (b) spectra of 10b, O_2_ adduct of BCB-NHPhCO_2_Et**


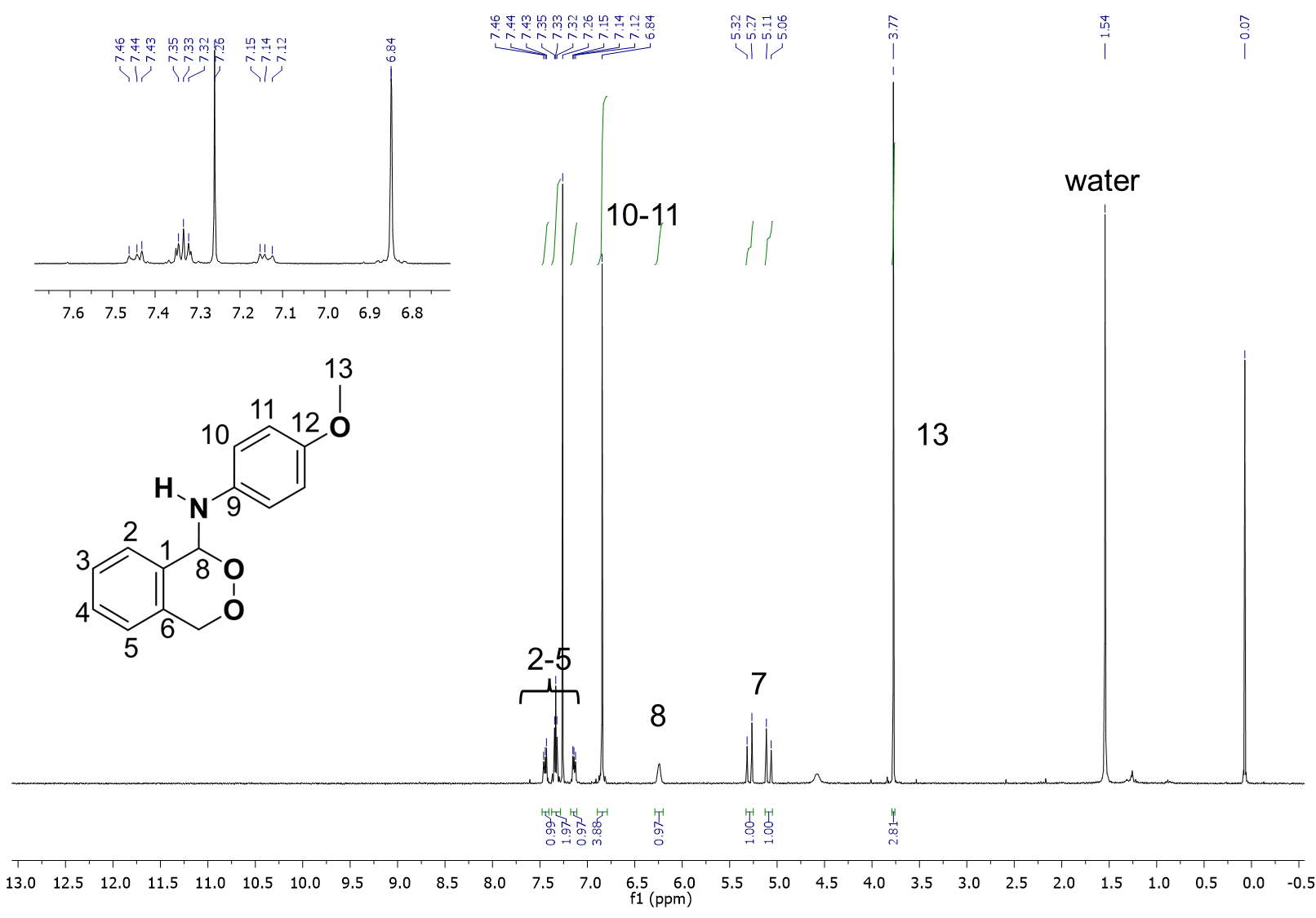


Figure S119. **^1^H NMR spectrum of 10c, O_2_ adduct of BCB-NHPhOMe**


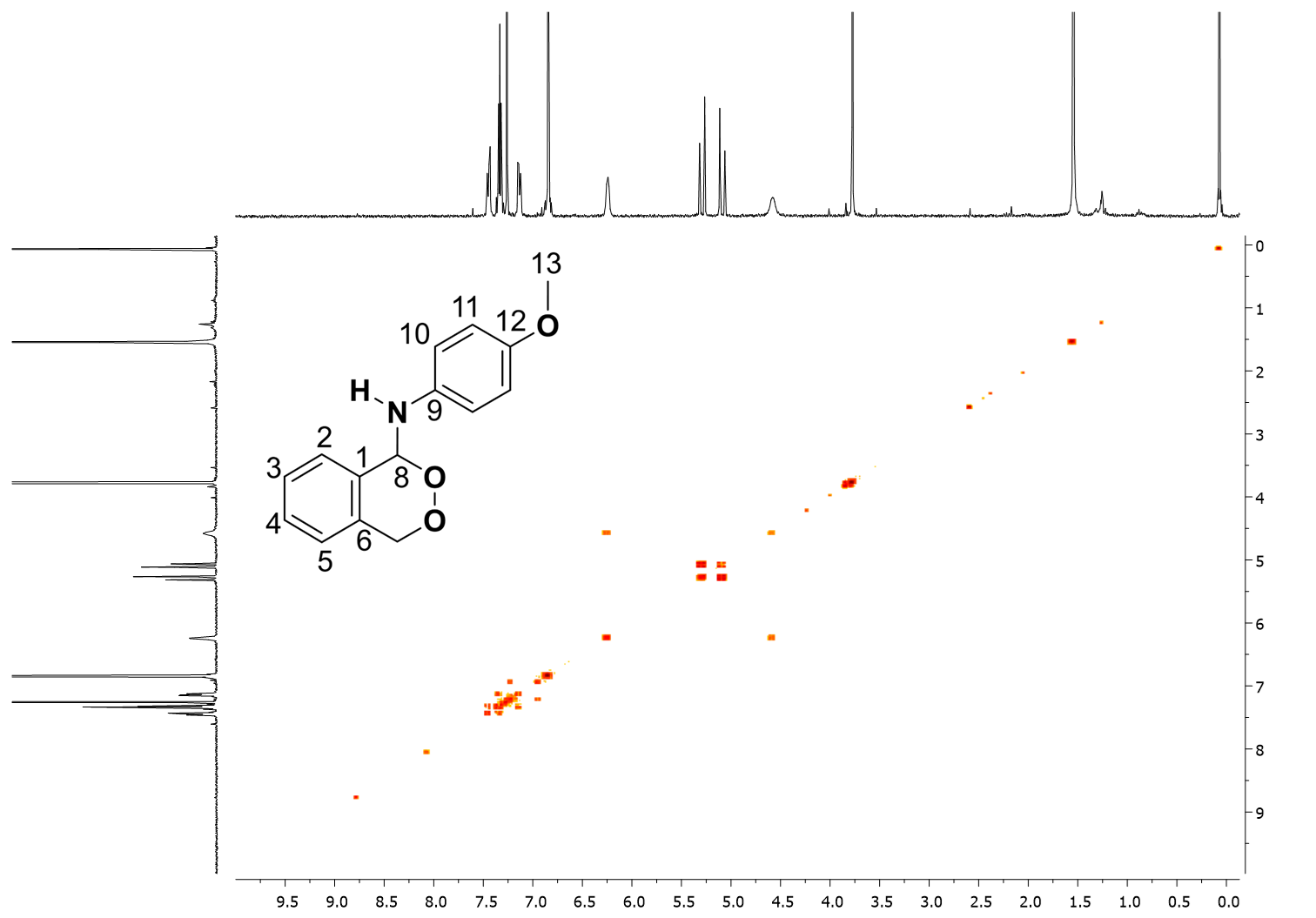


Figure S120. **COSY** **NMR spectrum of 10c, O_2_ adduct of BCB-NHPhOMe**


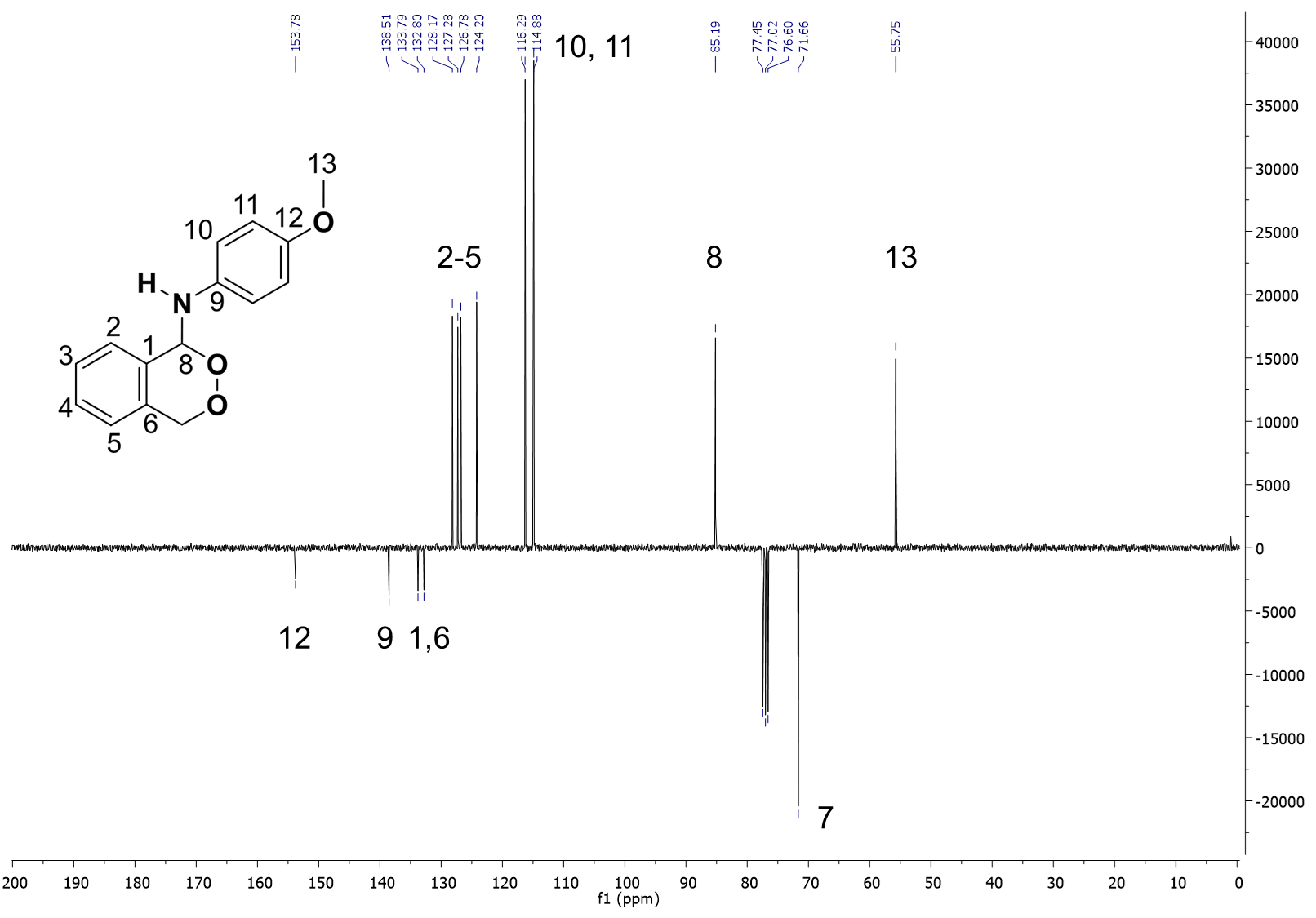


Figure S121. **^13^C-APT NMR spectrum of 10c, O_2_ adduct of BCB-NHPhOMe**


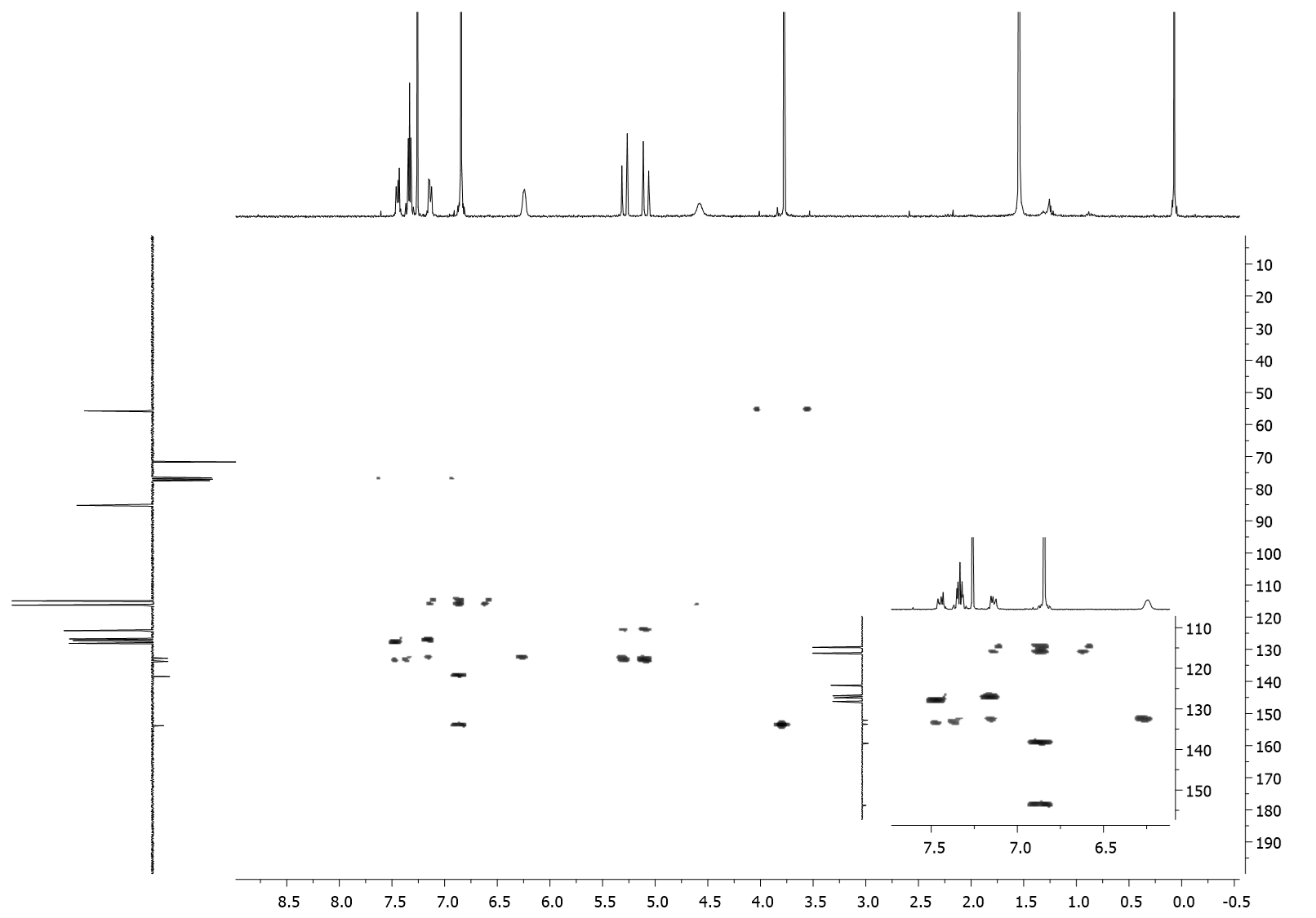


Figure S122. **HMQC NMR spectrum of 10c, O_2_ adduct of BCB-NHPhOMe**

Figure S123. **^1^H NMR spectrum of 10d, O_2_ adduct of BCB-NHPh**

Figure S124. **COSY NMR spectrum of 10d, O_2_ adduct of BCB-NHPh**

Figure S125. **^13^C-APT NMR spectrum of 10d, O_2_ adduct of BCB-NHPh**

Figure S126. **HMQC NMR spectrum of 10d, O_2_ adduct of BCB-NHPh**

***9/ Molecular structures of 10a and 10b***

Single crystals of **10a** were obtained after slow evaporation of a CDCl_3_ solution. CCDC 2492651 contains the supplementary crystallographic data for **10a**.

The BCB peroxide **10a** crystallized as a solid solution, comprising two conformers **10a-A** and **10a-B**. The unit cell of **10a** shows the co-crystallization of the two enantiomers R and S (Figure S127). The O-O bond lengths (Å) are showed in Table S1.

Table S1. O-O bond lengths (Å) in crystal structures of **10a** and **10b**

| O-O bond lengths (Å) | | | |
| --- | --- | --- | --- |
| Conformer **10a-A** | Conformer **10a-B** | Conformer **10b-A** | Conformer **10b-B** |
| O1-O2 = 1.438(3) | O3-O4 = 1.460(5) | O3-O5 = 1.479(10) | O6-O7 = 1.431(9) |

Figure S127. **Molecular structures of conformers 10a-A and 10a-B (enantiomers R), and unit cell of crystal structure of 10a (conformer A: O and F atoms in dark red and green; conformer B: O and F atoms in light red and green, respectively).**

Single crystals of **10b** were obtained after slow evaporation of a CDCl_3_ solution. CCDC 2494057 contains the supplementary crystallographic data for **10b**.

The BCB peroxide **10b** crystallized as an enantiomeric solid solution, comprising two conformers **10b-A** and **10b-B**. The molecular structures of **10b-A** and **10b-B** (enantiomers R) and their unit cells are presented in Figure S128. The O-O bond lengths (Å) are showed in Table S1.

Figure S128. **Molecular structures of conformers 10b-A and 10b-B (enantiomers R), and their respective unit cells.**

The two conformers of **10a** and **10b** present similar O-O bond distances between 1.431 and 1.479 Å (Table S1). These bond distances are in the same order of magnitude than ones in the molecular structures of BCB peroxide conformers reported by Wahl et al. (O-O distances of 1.4559(19) and 1.47(2) Å).^11^ Similarly Chiba et al. observed a O-O bond distance of 1.4648(9) Å from the molecular structure of the peroxide derivative obtained from the Cu-catalyzed oxidation of 2-cyclohexyl-benzonitrile in presence of p-TolMgBr as Grignard reactant.^12^

**10/ Computational part**

**Natural Bond Orbital NBO calculations**

NBO calculations were performed on the closed benzocyclobutene (BCB) structures. Geometry optimizations were carried out at the M06-2X/6-31G(d) level of theory, and single-point energy calculations were subsequently performed at the M06-2X/6-311++G(3df,3pd) level. For two orbitals i (donor orbital) and j (acceptor orbital), the interaction energy **E_int_** depends on the energy difference between these orbitals (ε_i_ – ε_j_), donor orbital population q_i_ and on their overlap integral F_(i,j)_. In case of BCB E_int_, we focus on the internal interaction energy E_int_ between the lone pair of the heteroatom and the antibonding σ* orbital (Table 1).

$$\boldsymbol{E}_{\boldsymbol{int}}\boldsymbol{=}\boldsymbol{q}_{\boldsymbol{i}} \frac{{\boldsymbol{F}_{\boldsymbol{(i,j)}}}^{\boldsymbol{2}}}{\boldsymbol{\varepsilon}_{\boldsymbol{i}}\boldsymbol{-}\boldsymbol{\varepsilon}_{\boldsymbol{j}}}$$

**Natural Orbital Occupation Numbers NOON**

The diradical character of the ring-opened intermediates was estimated using Natural Orbital Occupation Numbers with the GAMESS package.^13^ o-QDM, o-QDM-OMe, o-QDM-NHPhCO_2_Et and o-QDM-N(COMe)PhCO_2_Et compounds were fully optimized at CASSCF(8,8)/6-31G(d) level of theory (*Scheme S2*).

*Scheme S2* : **Representation of the natural orbitals in active space by CASSCF(8,8)/6-31G(d) for o-QDM, o-QDM-OMe, o-QDM-NH-PhCO_2_Et and o-QDM-N(COMe)-PhCO_2_Et and their respective occupation numbers (HONO and LUNO are in red).**

**
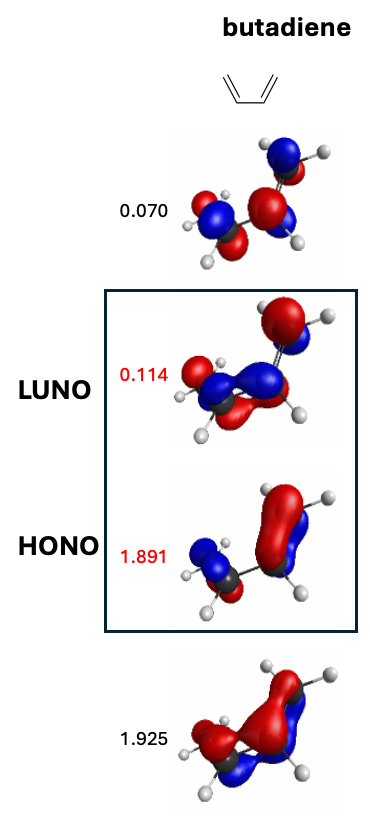
**

*Scheme S3* : **Representation of the natural orbitals in active space by CASSCF(4,4)/6-31G(d) for butadiene and its respective occupation numbers (HONO and LUNO are in red).**

Table S2. Determination of the diradical character **y** of o-QDM

| ***o*-QDM** | **HONO** | **LUNO** | **y_LUNO_^a^** | **y_1-(HONO-LUNO)/2_** |
| --- | --- | --- | --- | --- |
| ***o*-QDM-H** | **1.814** | **0.194** | **19.4%** | **19%** |
| ***o*-QDM-OMe** | **1.825** | **0.181** | **18.1%** | **17.8%** |
| ***o*-QDM-NHPhCO_2_Et** | **1.857** | **0.147** | **14.7%** | **14.5%** |
| ***o*-QDM-N(COMe)PhCO_2_Et** | **1.855** | **0.152** | **15.2%** | **14.9%** |
| ***cis*-butadiene** | **1.891** | **0.114** | **11.4%** | **11.2%** |
| ^a^ Determination of y based on LUNO value  b Determination of y based on equation y = $1-\left( HONO-LUNO \right)/2$ | | | | |

We employed a simple two-electron, two-orbital (2e,2o) model considering the HONO and LUNO within a singlet diradical framework in diradicaloid system. In this model, the diradical character $y$can be estimated from the occupation number of the LUNO. Alternatively, both HONO and LUNO occupation numbers can be used to evaluate the diradical character according to the relation $y=1-(n_{\text{HONO}}-n_{\text{LUNO}})/2$.

As shown in Table S2, the two approaches yield very similar values of $y$.

**11/ References**

1. F. Le Vaillant, M. Garreau, S. Nicolai, G. Gryn'ova, C. Corminboeuf and J. Waser, Fine-tuned organic photoredox catalysts for fragmentation-alkynylation cascades of cyclic oxime ethers, *Chem. Sci.*, 2018, **9**, 5883-5889.

2. O. V. Dolomanov, L. J. Bourhis, R. J. Gildea, J. A. K. Howard and H. Puschmann, OLEX2: a complete structure solution, refinement and analysis program, *J. Appl. Crystallogr.*, 2009, **42**, 339-341.

3. G. M. Sheldrick, SHELXT - Integrated space-group and crystal-structure determination, *Acta Crystallogr., Sect. A: Found. Adv.*, 2015, **71**, 3-8.

4. G. M. Sheldrick, Crystal structure refinement with SHELXL, *Acta Crystallogr., Sect. C: Struct. Chem.*, 2015, **71**, 3-8.

5. M. R. DeCamp and L. A. Viscogliosi, 1-Bromobenzocyclobutene: a convenient entry into the benzocyclobutene ring system, *J. Org. Chem.*, 1981, **46**, 3918-3920.

6. J. G. Park, J. Sun, C. D. Gilmore, J. Z. Zhang, P. D. Hustad, P. Trefonas, III and K. M. O'Connell, *Korea Pat.,* KR2015079486, 2015.

7. K. Chino, T. Takata and T. Endo, Polymerization of o-quinodimethanes bearing electron-donating groups in situ formed by thermal isomerization of benzocyclobutenes, *Macromolecules*, 1997, **30**, 6715-6720.

8. M. Azadi-Ardakani and T. W. Wallace, 3,6-Dimethoxybenzocyclobutenone: a reagent for quinone synthesis, *Tetrahedron*, 1988, **44**, 5939-5952.

9. Q. Wang and N. Zheng, A Photocatalyzed Synthesis of Naphthalenes by Using Aniline as a Traceless Directing Group in [4 + 2] Annulation of Amino-benzocyclobutenes with Alkynes, *ACS Catal.*, 2017, **7**, 4197-4201.

10. , *Article in preparation*.

11. H. Maag, M. Schmitz, A. Sandvoß, D. Mundil, A. Pedada, F. Glaser, C. Kerzig and J. M. Wahl, Photocyclization of Fluorinated Acetophenones Unlocks an Efficient Way to Solar Energy Storage, *J. Am. Chem. Soc.*, 2024, **146**, 32701-32707.

12. L. Zhang, G. Y. Ang and S. Chiba, Copper-Catalyzed Benzylic C−H Oxygenation under an Oxygen Atmosphere via N-H Imines as an Intramolecular Directing Group, *Org. Lett.*, 2011, **13**, 1622-1625.

13. M. W. Schmidt, K. K. Baldridge, J. A. Boatz, S. T. Elbert, M. S. Gordon, J. H. Jensen, S. Koseki, N. Matsunaga, K. A. Nguyen, S. Su, T. L. Windus, M. Dupuis and J. A. Montgomery Jr, General atomic and molecular electronic structure system, *J. Comput. Chem.*, 1993, **14**, 1347-1363.
